# Facile and Promising Method for Michael Addition of Indole and Pyrrole to Electron-Deficient *trans*-****β****-Nitroolefins Catalyzed by a Hydrogen Bond Donor Catalyst Feist's Acid and Preliminary Study of Antimicrobial Activity

**DOI:** 10.1155/2014/649197

**Published:** 2014-01-16

**Authors:** Abdullah M. A. Al Majid, Mohammad Shahidul Islam, Assem Barakat, Mohamed H. M. Al-Agamy, Mu. Naushad

**Affiliations:** ^1^Department of Chemistry, Faculty of Science, King Saud University, P.O. Box 2455, Riyadh 11451, Saudi Arabia; ^2^Department of Chemistry, Faculty of Science, Alexandria University, P.O. Box 426, Ibrahimia, Alexandria 21321, Egypt; ^3^Division of Microbiology, Pharmaceutics Department, College of Pharmacy, King Saud University, P.O. Box 2457, Riyadh 11451, Saudi Arabia

## Abstract

The importance of cooperative hydrogen-bonding effects has been demonstrated using novel 3-methylenecyclopropane-1,2-dicarboxylic acid (Feist's acid (FA)) as hydrogen bond donor catalysts for the addition of indole and pyrrole to *trans*-**β**-nitrostyrene derivatives. Because of the hydrogen bond donor (HBD) ability, Feist's acid (FA) has been introduced as a new class of hydrogen bond donor catalysts for the activation of nitroolefin towards nucleophilic substitution reaction. It has effectively catalyzed the Michael addition of indoles and pyrrole to **β**-nitroolefins under optimum reaction condition to furnish the corresponding Michael adducts in good to excellent yields (up to 98%). The method is general, atom-economical, convenient, and eco-friendly and could provide excellent yields and regioselectivities. Some newly synthesized compounds were for examined *in vitro* antimicrobial activity and their preliminary results are reported.

## 1. Introduction

In the field of advanced organic synthesis, Michael addition reaction is one of the most powerful tools for carbon-carbon bond construction reactions [1–6]. Nitroolefins are very attractive among the many Michael acceptors because of their strong electron-withdrawing nitro group which could be easily transformed into a wide variety of different functionalities [7–9] that may lead to synthesizing important biologically active building blocks and products [10]. Until now a numerous number of catalysts have been reported in the literature, mainly for the Michael addition reactions of heteroarenes to carbonyls [11, 12]. Moreover a remarkable number of catalysts have been used to catalyze the Michael addition [13–21] reaction of indoles and pyrrole to *trans*-**β**-nitroolefins; however, most of them were Lewis acids [22–25]. Recently some small organic molecules with anion recognition abilities have provided a great deal of inspiration for the development of hydrogen-bonding catalysis [26–28]; in this context, hydrogen bond donor (HBD) catalysis has received some achievement in exploiting the anion recognition abilities offered by urea, thiourea, and guanidinium functionalities [29–31], silanediols [32, 33], silica gel [34], 2-aminopyridinium ions [35–38], sulfamic acid (SA) [9], dipicolinic acid [26], which were found to be highly potent in catalyzing the Michael addition reaction through hydrogen bonding catalysis [26, 39–47]. Yet such small organic molecules with hydrogen bond donor (HBD) ability received little attention in the context of noncovalent catalysis [48]. Excited by the potential of such hydrogen bond donor small molecule, we have initiated a program in our laboratory dedicated toward pioneering the development of Feist's acid (FA) [49, 50] as a new class of catalysts that operate through hydrogen bonding interactions.

Indole and its analogs constitute the active class of compounds possessing wide spectrum of biological activities. A variety of indole derivatives have emerged that possess a range of bioactivities including potent anticancer, antiviral, anti-inflammatory, anti-hypertensive, anti-asthmatic and anti-tubercular properties. Some of these compounds are also known to possess anti-inflammatory and analgesic properties [51–53]. Pyrrole heterocyclic derivatives were reported as having important synthetic and biological activities such as COX-1/COX-2 inhibitors and cytotoxic activity against a variety of marine and human tumor models [54–59].

In this communication, we report initial successes with FA catalysis and its application toward the activation of nitroolefin via hydrogen-bonding mechanistic pathway, which effectively catalyze Michael addition of indoles and pyrrole to nitroolefins under optimum reaction condition to afford the corresponding Michael adducts in good to excellent yields. Some newly synthesized compounds were subjected to *in vitro *antimicrobial activity.

## 2. Experimental

### 2.1. General Information

Glassware was oven-dried overnight at 120°C before use. Reactions were performed under an inert atmosphere using an argon filled glove box and standard Schlenk-line techniques. All the reactions were monitored by TLC analysis using Merck Silica Gel 60 F-254 thin layer plates. Column chromatography was performed on silica gel 100–200 mesh.

#### 2.1.1. Materials

Petroleum ether (PE), hexane, and ethyl acetate for column chromatography were distilled prior to use. CH_2_Cl_2_ and EtOH were distilled from P_2_O_5_ and Mg, respectively, and stored on 4 Å molecular sieves. Tetrahydrofuran, benzene, and toluene were distilled from sodium benzophenone ketyl. Acetonitrile and dimethylformamide were dried by distillation over calcium hydride. Nitroolefins **2(a–i) **were prepared according to procedures reported in literature [60].

#### 2.1.2. Instrumentation

NMR spectra were recorded with a Jeol spectrometer at 400 MHz (^1^H-NMR) and 100 MHz (^13^C-NMR.). The chemical shifts (**δ** in ppm) were reported down field from tetramethylsilane (TMS, **δ** scale) with the deuterated solvent resonance referenced as internal standard. Elemental analyses were performed on a Perkin Elmer 2400 Elemental Analyzer. IR spectra were obtained using FTIR-800 Model. Mass spectrometric analysis was conducted by using ESI mode on AGILENT Technologies 6410-triple quad LC/MS instrument.

### 2.2. General Procedure for the Michael Addition Reaction of Indole with *β*-Nitroolefins Catalyzed by Feist's Acid (**GP1**)

Indole **4** (50 mg, 0.425 mmol), **β**-nitroolefins **2(a–i)** (0.425 mmol), and a catalytic amount of Feist's acid (**1**) (6 mg, 0.084 mmol, 10 mol%) in dry ethanol (3 mL) were charged into a Schlenk tube under an argon atmosphere. The reaction was then stirred at 50°C for 48–72 hours. The reaction mixture was monitored by TLC until starting material was completely consumed. Then the solvent was removed under vacuum. The crude products were isolated by column chromatography to afford pure Michael adducts (**7a–i**). The analytical data of the known compounds were in accordance with reported literature [58].

#### 2.2.1. 3-(2-Nitro-1-phenylethyl)-1H-indole (Table 2, Entry 1, **7a**)

Indole **4** (50 mg, 0.43 mmol) and **β**-nitrostyrene **2a** (64 mg, 0.43 mmol) in dry ethanol (3 mL) were reacted in the presence of Feist's acid (**1**) (6 mg, 0.084 mmol, 10 mol%) according to **GP1**. The product was purified by column chromatography on silica (EtOAc/hexane 1 : 9) yielded **7a **as yellow oil [58] (111 mg, 0.42 mmol, 97%). IR (KBr): 3417, 15461375, 742, 700, 587 cm^−1^; ^1^H-NMR (CDCl_3_, 400 MHz) **δ** 4.89–4.99 (m, 1H, CHC**H**_2(a)_), 5.02–5.12 (m, 1H, CHC**H**_2(b)_), 5.15–5.23 (m, 1H, C**H**CH_2_), 7.01–7.11 (m, 2H, Ar**H**), 7.17–7.24 (m, 1H, Ar**H**), 7.25 (s, 1H, NHC**H**), 7.26–7.36 (m, 5H, Ar**H**), 7.44 (d, *J* = 8.0 Hz, 1H. Ar**H**), 8.09 (s, 1H, N**H** of Indole); ^13^C-NMR (CDCl_3_, 100 MHz): **δ** 41.6, 79.9, 111.4, 114.5, 119.0, 120.0, 121.7, 122.8, 126.7, 127.7, 127.8, 129.0, 136.6, 139.2; [Anal. Calcd. for C_16_H_14_N_2_O_2_: C, 72.16; H, 5.30; N, 10.52; found: C, 72.37; H, 5.23; N, 10.41]; LC/MS (ESI): M^+^, found 266.08, C_16_H_14_N_2_O_2_ requires 266.11.

#### 2.2.2. 3-[1-(4-Methylphenyl)-2-nitroethyl]-1H-indole (Table 2, Entry 2, **7b**)

Indole **4** (50 mg, 0.43 mmol) and **β**-nitroolefin **2b** (70 mg, 0.43 mmol) in dry ethanol (3 mL) were reacted in the presence of Feist's acid (**1**) (6 mg, 0.084 mmol, 10 mol%) according to **GP1**. The product was purified by column chromatography on silica (EtOAc/hexane 1 : 9) yielded **7b **as yellow oil [58] (117 mg, 0.42 mmol, 97.2%). IR (KBr): 3418, 1547, 1377, 739 cm^−1^; ^1^H-NMR (CDCl_3_, 400 MHz) **δ** 2.31 (s, 1H, C**H**_3_), 4.86–4.98 (m, 1H, CHC**H**_2(a)_), 5.01–5.10 (m, 1H, CHC**H**_2(b)_), 5.10–5.20 (m, 1H, C**H**CH_2_), 6.98–7.02 (m, 2H, Ar**H**), 7.06–7.25 (m, 5H, Ar**H**), 7.34 (d, *J* = 8.0 Hz, 1H, Ar**H**), 7.46 (d, *J* = 8.0 Hz, 1H. Ar**H**), 8.06 (s, 1H, N**H** of Indole); ^13^C-NMR (CDCl_3_,_,_ 100 MHz): **δ** 21.1, 41.3, 80.0, 111.4, 114.7, 119.0, 120.0, 121.6, 122.7, 126.2, 127.7, 129.7, 136.2, 136.6, 137.3; [Anal. Calcd. for C_17_H_16_N_2_O_2_: C, 72.84; H, 5.75; N, 9.99; found: C, 73.03; H, 5.66; N, 10.11]; LC/MS (ESI): M^+^, found 280.16, C_17_H_16_N_2_O_2_ requires 280.12.

#### 2.2.3. 3-(1-(4-Methoxyphenyl)-2-nitroethyl)-1H-indole (Table 2, Entry 3, **7c**)

Indole **4** (50 mg, 0.43 mmol) and **β**-nitroolefin **2c** (77 mg, 0.43 mmol) in dry ethanol (3 mL) were reacted in the presence of Feist's acid (**1**) (6 mg, 0.084 mmol, 10 mol%) according to **GP1**. The product was purified by column chromatography on silica (EtOAc/hexane 1 : 9) yielded **7c **as white solid (107 mg, 0.36 mmol, 84%). m.p. 149−151°C [Lit [58] m.p. 148–150°C]; IR (KBr): 3373, 1545, 1509, 1458, 1420, 1374, 1241, 1179, 1027, 742, 608, 548, 525 cm^−1^; ^1^H-NMR (CDCl_3_, 400 MHz) **δ** 3.76 (s, 1H, OC**H**_3_), 4.84–4.93 (m, 1H, CHC**H**_2(a)_), 5.00–5.08 (C**H**CH_2_m, 1H, CHC**H**_2(b)_), 5.08–5.17 (m, 1H,), 6.84 (d, *J* = 5.2 Hz, 2H. Ar**H**), 7.01 (s, 1H, NHC**H**), 7.02–7.10 (m, 1H, Ar**H**), 7.19–7.28 (m, 3H, Ar**H**), 7.36 (d, *J* = 8.0 Hz, 1H. Ar**H**), 7.44 (d, *J* = 8.0 Hz, 2H. Ar**H**), 8.07 (s, 1H, N**H** of Indole); ^13^C-NMR (CDCl_3_, 100 MHz): **δ** 40.9, 55.3, 80.0, 111.4, 114.3, 119.1, 120.0, 121.5, 122.8, 126.1, 128.9, 131.2, 136.6, 139.3, 158.9; [Anal. Calcd. for C_17_H_16_N_2_O_3_: C, 68.91; H, 5.44; N, 9.45; found: C, 79.09; H, 5.53; N, 9.36]; LC/MS (ESI): M^+^, found 296.18, C_17_H_16_N_2_O_2_ requires 296.12.

#### 2.2.4. 3-(1-(4-Chlorophenyl)-2-nitroethyl)-1H-indole (Table 2, Entry 4, **7d**)

Indole **4** (50 mg, 0.43 mmol) and **β**-nitroolefin** 2d** (79 mg, 0.43 mmol) in dry ethanol (3 mL) were reacted in the presence of Feist's acid (**1**) (6 mg, 0.084 mmol, 10 mol%) according to **GP1**. The product was purified by column chromatography on silica (EtOAc/hexane 1 : 9) yielded **7d **as oily liquid (124 mg, 0.42 mmol, 97.4%). IR (KBr): 3417, 1548, 1376, 1091, 736, 423 cm^−1^; ^1^H-NMR (CDCl_3_, 400 MHz) **δ** 4.83–4.96 (m, 1H, CHC**H**_2(a)_), 5.02–5.10 (m, 1H, CHC**H**_2(b)_), 5.10–5.22 (m, 1H, C**H**CH_2_), 6.96–7.03 (m, 1H. Ar**H**), 7.04–7.12 (m, 1H, Ar**H**), 7.15–7.34 (m, 4H, Ar**H**), 7.34–7.46 (m, 3H, Ar**H**), 8.12 (s, 1H, N**H** of Indole); ^13^C-NMR (CDCl_3,_ 100 MHz): **δ** 41.0, 79.4, 111.6, 114.0, 118.1, 118.9, 120.2, 121.6, 123.0, 126.0, 129.2, 133.5, 136.6, 137.8; [Anal. Calcd. for C_16_H_13_ClN_2_O_2_: C, 63.90; H, 4.36; N, 9.31; found: C, 64.11; H, 4.05; N, 9.57]; LC/MS (ESI): M^+^ & [M+2]^+^, found 300.01 & 302.05, C_16_H_13_ClN_2_O_2_ requires 300.07.

#### 2.2.5. 3-(1-(4-Bromophenyl)-2-nitroethyl)-1H-indole (Table 2, Entry 6, **7e**)

Indole **4** (50 mg, 0.43 mmol) and **β**-nitroolefin **2e** (98 mg, 0.43 mmol) in dry ethanol (3 mL) were reacted in the presence of Feist's acid (**1**) (12 mg, 0.168 mmol, 20 mol%) according to **GP1**. The product was purified by column chromatography on silica (EtOAc/hexane 1 : 9) yielded **7e **as yellow oil (130 mg, 0.38 mmol, 88%). IR (KBr): 3405, 1539, 1380, 1006, 743, 594, 533, 424 cm^−1^; ^1^H-NMR (CDCl_3_, 400 MHz) **δ** 4.86–4.98 (m, 1H, CHC**H**_2(a)_), 4.99–5.10 (m, 1H, CHC**H**_2(b)_), 5.10–5.18 (m, 1H, C**H**CH_2_), 6.95–7.02 (m, 1H. Ar**H**), 7.02–7.14 (m, 1H, Ar**H**), 7.15–7.27 (m, 4H, Ar**H**), 7.29–7.48 (m, 3H, Ar**H**), 8.11 (s, 1H, N**H** of Indole); ^13^C-NMR (CDCl_3_, 100 MHz): **δ** 41.1, 79.3, 111.8, 113.9, 118.9, 120.2, 121.6, 123.0, 126.0, 129.6, 132.2, 136.6, 138.3, 140.0; [Anal. Calcd. for C_16_H_13_BrN_2_O_2_: C, 55.67; H, 3.80; N, 8.12; found: C, 55.27; H, 3.73; N, 7.98]; LC/MS (ESI): M^+^ & [M+2]^+^, found 344.10 & 346.13, C_16_H_13_BrN_2_O_2_ requires 344.02.

#### 2.2.6. 3-(1-(4-Nitrophenyl)-2-nitroethyl)-1H-indole (Table 2, Entry 8, **7f**)

Indole **4** (50 mg, 0.43 mmol) and **β**-nitroolefin** 2f** (83 mg, 0.43 mmol) in dry ethanol (3 mL) were reacted in the presence of Feist's acid (**1**) (12 mg, 0.168 mmol, 20 mol%) according to **GP1**. The product was purified by column chromatography on silica (EtOAc/hexane 1 : 9) yielded **7f **as yellow oil (94 mg, 0.30 mmol, 70%). IR (KBr): 3418, 1549, 1520, 1341, 713, 424 cm^−1^; ^1^H-NMR (CDCl_3_, 400 MHz) **δ** 5.00–5.19 (m, 2H, CHC**H**_2_), 5.79–5.96 (m, 1H, C**H**CH_2_), 6.96–7.08 (m, 1H. Ar**H**), 7.08–7.61 (m, 7H. Ar**H**), 7.90 (d, *J* = 8.0 Hz, 1H. Ar**H**), 8.16 (s, 1H, N**H** of Indole); ^13^C-NMR (CDCl_3,_ 100 MHz): **δ** 36.5, 80.1, 111.5, 112.9, 118.6, 120.4, 122.1, 123.1, 125.2, 126.0, 128.7, 130.0, 133.3, 136.4; [Anal. Calcd. for C_16_H_13_N_3_O_4_: C, 61.73; H, 4.21; N, 13.50; found: C, 68.05; H, 4.42; N, 13.69]; LC/MS (ESI): M^+^, found 344.08, C_16_H_13_N_3_O_4_ requires 311.09.

#### 2.2.7. 3-(1-(2,4-Dichlorophenyl)-2-nitroethyl)-1H-indole (Table 2, Entry 10, **7g**)

Indole **4** (50 mg, 0.43 mmol) and *β*-nitroolefin **2g** (93 mg, 0.43 mmol) in dry ethanol (3 mL) were reacted in the presence of Feist's acid (**1**) (12 mg, 0.168 mmol, 20 mol%) according to **GP1**. The product was purified by column chromatography on silica (EtOAc/hexane 1 : 9) yielded **7g **as yellow oil (140 mg, 0.42 mmol, 97.5%). IR (KBr): 3417, 1549, 1464, 1376, 1101, 820, 738 cm^−1^; ^1^H-NMR (CDCl_3_, 400 MHz) **δ** 4.88–5.01 (m, 2H, CHC**H**_2_), 5.62–5.75 (m, 1H, CHC**H**_2_), 7.03–7.16 (m, 4H. Ar**H**), 7.18–7.27 (m, 1H, Ar**H**), 7.32–7.42 (m, 2H, Ar**H**), 7.45 (s, 1H, Ar**H**), 8.16 (s, 1H, N**H** of Indole); ^13^C-NMR (CDCl_3_, 100 MHz): **δ** 37.7, 80.0, 111.5, 112.9, 118.9, 120.3, 122.0, 123.1, 126.0, 127.7, 129.9, 132.2, 134.0, 134.8, 135.3, 136.6; [Anal. Calcd. for C_16_H_12_Cl_2_N_2_O_2_: C, 57.33; H, 3.61; N, 8.36; found: C, 57.08; H, 3.43; N, 8.19]; LC/MS (ESI): M^+^ & [M+2]^+^, found 334.12 & 336.09, C_16_H_12_Cl_2_N_2_O_2_ requires 334.03.

#### 2.2.8. 3-(1-(2,6-Dichlorophenyl)-2-nitroethyl)-1H-indole (Table 2, Entry 12, **7h**)

Indole **4** (50 mg, 0.43 mmol) and *β*-nitroolefin **2h** (93 mg, 0.43 mmol) in dry ethanol (3 mL) were reacted in the presence of Feist's acid (**1**) (12 mg, 0.168 mmol, 20 mol%) according to **GP1**. The product was purified by column chromatography on silica (EtOAc/hexane 1 : 9) yielded **7h **yellow oily (108 mg, 0.32 mmol, 75.2%). IR (KBr): 3417, 1549, 1464, 1376, 1101, 820, 738 cm^−1^; ^1^H-NMR (CDCl_3_, 400 MHz) **δ** 5.28–5.52 (m, 2H, CHC**H**_2_), 6.12–6.34 (m, 1H, C**H**CH_2_), 6.95–7.10 (m, 1H. Ar**H**), 7.10–7.24 (m, 3H. Ar**H**), 7.24–7.65 (m, 4H. Ar**H**), 8.14 (s, 1H, N**H** of Indole); ^13^C-NMR (CDCl_3_, 100 MHz): **δ** 38.0, 78.9, 111.4, 114.0, 115.1, 117.8, 119.0, 120.1, 121.6, 122.5, 126.4, 129.4, 134.2, 136.1; [Anal. Calcd. for C_16_H_12_Cl_2_N_2_O_2_: C, 57.33; H, 3.61; N, 8.36; found: C, 57.08; H, 3.43; N, 8.19]; LC/MS (ESI): M^+^ & [M+2]^+^; found 334.09 & 336.07, C_16_H_12_Cl_2_N_2_O_2_ requires 334.03.

#### 2.2.9. 3-(1-Ferrocenyl)-2-nitroethyl)-1H-indole (Table 1, Entry 12, **7i**)

Indole **4** (50 mg, 0.43 mmol) and *β*-nitro styrene **2i** (118 mg, 0.43 mmol) in dry ethanol (5 mL) were reacted in the presence of Feist's acid (**1**) (12 mg, 0.168 mmol, 20 mol%) according to **GP1**. The product was purified by column chromatography on silica (EtOAc/hexane 1 : 9) yielded **7i **as oily liquid (78 mg, 0.20 mmol, 46.4%). IR (KBr): 3417, 1549, 1464, 1376, 1101, 820, 738 cm^−1^; ^1^H-NMR (CDCl_3_, 400 MHz) **δ** 4.09 (s, 5H, protons of C*p*), 4.11–4.25 (m, 4H, protons of C*p*), 4.83–5.89 (m, 1H, C*p*C**H**CH_2_), 4.93–5.65 (m, 2H, C*p*CHC**H**_2_), 7.01 (s, 1H. Ar**H**), 7.11 (t, *J* = 5.2 Hz, 1H. Ar**H**), 7.18 (t, *J* = 5.2 Hz, 1H. Ar**H**), 7.34 (d, *J* = 8.0 Hz, 1H. Ar**H**), 7.57 (d, *J* = 8.0 Hz, 1H. Ar**H**), 8.06 (s, 1H, N**H** of Indole); ^13^C-NMR (CDCL_3_, 100 MHz): **δ** 36.7, 66.8, 67.7, 68.2, 69.0, 80.6, 111.5, 113.9, 119.1, 119.9, 121.8, 122.5, 126, 129.3; [Anal. Calcd. for C_20_H_18_FeN_2_O_2_: C, 64.19; H, 4.84; N, 7.48; found: C, 63.97; H, 4.71; N, 7.59]; LC/MS (ESI): M^+^, found 374.22, C_20_H_18_FeN_2_O_2_ requires 374.19.

### 2.3. General Procedure for the Michael Addition Reaction of Indole with Pyrrole Catalyzed by Feist's Acid (**GP2**)

Pyrrole **5** (100 mg, 0.86 mmol), *β*-nitrostyrene **2(a–i)** (0.86 mmol), and a catalytic amount of Feist's acid (**1**) (12 mg, 0.168 mmol, 10 mol%) in dry isopropanol (7 mL) were charged into a Schlenk tube under an argon atmosphere. The reaction was then stirred at 50°C for 24–48 hours. The reaction mixture was monitored by TLC until starting material was completely consumed. Then the solvent was removed under vacuum. The crude products were isolated by column chromatography to afford pure Michael adducts **8(a–i**) as major region-isomer, **9(a–d)** and **9h** as minor region-isomer.

#### 2.3.1. 2-(2-Nitro-1-phenylethyl)-1H-pyrrole (Table 3, Entry 1, **8a**) and 2,5-Bis(2-nitro-1-phenylethyl)-1H-pyrrole (Table 3, Entry 1, **9a**)

Pyrrole **5** (100 mg, 0.86 mmol) and **β**-nitrostyrene **2a** (128 mg, 0.86 mmol) in isopropanol (7 mL) were reacted in the presence of Feist's acid (**1**) (12 mg, 0.168 mmol, 10 mol%) according to **GP2**. The products were isolated by column chromatography on silica (EtOAc/hexane 0.5 : 9.5) yielded as yellow solid **8a **(major region-isomer) (165 mg, 0.74 mmol, 86.0%) and **9a **(minor region-isomer) as yellow oil (40 mg, 0.11 mmol, 12.7%). (*Major region-isomer* **8a**): IR (KBr): 3423, 1548, 1376, 723, 703, 524 (cm^−1^); ^1^H-NMR (CDCl_3_, 400 MHz) **δ** 4.69–4.85 (m, 1H, CHC**H**_2(a)_), 4.85–4.93 (m, 1H, CHC**H**_2(b)_), 4.93–5.02 (m, 1H, C**H**CH_2_), 6.03–6.10 (m, 1H, Ar**H**), 6.14–6.17 (m, 1H, Ar**H**), 6.64–6.72 (m, 1H. Ar**H**), 7.17–7.26 (m, 2H, Ar**H**), 7.26–7.40 (m, 3H, Ar**H**), 7.84 (s, 1H,N**H** of pyrrole); ^13^C-NMR (CDCl_3_, 100 MHz): **δ** 43.0 (**C**HCH_2_), 79.6 (CH**C**H_2_), 105.9 (Py**C**_**2**_), 108.8 (Py**C**_**3**_), 118.3 (Py**C**_**4**_), 128.0 (2C, Ph**C**_**2**_), 128.24 (Ph**C**_**4**_), 129.0 (Py**C**_**1**_), 129.3 (2C, Ph**C**_**3**_), 138.0 (Ph**C**_**1**_); [Anal. Calcd. for C_12_H_12_N_2_O_2_: C, 66.65; H, 5.59; N, 12.96; found: C, 66.45; H, 5.51; N, 13.11]; LC/MS (ESI): M^+^, found 216.11, C_12_H_12_N_2_O_2_ requires 216.09; (*minor region-isomer* **9a**): IR (KBr): 3407, 1548, 1376, 703, 524 cm^−1^; ^1^H-NMR (CDCl_3_, 400 MHz) **δ** 4.68–4.86 (m, 4H, CHC**H**_2_), 4.86–4.98 (m, 2H, C**H**CH_2_), 5.98 (d, *J* = 2.2 Hz, 2H, pyrrole), 7.05–7.18 (m, 4H, Ar**H**), 7.18–7.39 (m, 6H, Ar**H**), 7.54 (s, 1H, N**H** of pyrrole); ^13^C-NMR (CDCl_3,_ 100 MHz): **δ** 42.9 (**C**HCH_2_), 79.2 (CH**C**H_2_), 106.2 (Py**C**_**2**_), 106.6 (Py**C**_**2**_), 127.85 (Ph**C**_**2**_), 127.92 (Ph**C**_**4**_), 128.24 (Ph**C**_**3**_), 129.30 (Py**C**_**1**_), 138.1 (Ph**C**_**1**_); [Anal. Calcd. for C_20_H_19_N_3_O_4_: C, 65.74; H, 5.24; N, 11.50; found: C, 66.03; H, 5.37; N, 11.69]; LC/MS (ESI): M^+^, found 365.13, C_12_H_12_N_2_O_2_ requires 365.14.

#### 2.3.2. 2-(2-Nitro-1-(p-tolyl)ethyl)-1H-pyrrole (Table 3, Entry 2, **8b**) and 2,5-Bis(2-nitro-1-(p-tolyl)ethyl)-1H-pyrrole (Table 3, Entry 2, **9b**)

Pyrrole **5** (100 mg, 0.86 mmol) and **β**-nitroolefin **2b** (140 mg, 0.86 mmol) in isopropanol (7 mL) were reacted in the presence of Feist's acid (**1**) (12 mg, 0.168 mmol, 10 mol%) according to **GP2**. The products were isolated by column chromatography on silica (EtOAc/hexane 0.5 : 9.5) yielded as oily liquid **8b** (major region-isomer) (100 mg, 0.43 mmol, 51%) and **9b **(minor region-isomer) as yellow oil (70 mg, 0.18 mmol, 21%). (*Minor region-isomer* **8b**): IR (KBr): 3388, 1542, 1378, 716, 584 cm^−1^; ^1^H-NMR (CDCl_3_, 400 MHz) **δ** 2.25 (s, 1H, C**H**_3_), 4.67–4.73 (m, 1H, CHC**H**_2(a)_), 4.73–4.81 (m, 1H, CHC**H**_2(b)_), 4.82–4.93 (m, 1H, C**H**CH_2_), 5.99 (s, 1H, Ar**H**), 6.07–6.13 (m, 1H, Ar**H**), 6.58–6.59 (m, 1H. Ar**H**), 7.02–7.05 (m, 2H, Ar**H**), 7.05–7.08 (m, 2H, Ar**H**), 7.73 (s, 1H, N**H** of pyrrole); ^13^C-NMR (CDCl_3,_ 100 MHz): **δ** 21.2 (**C**H_3_), 42.7 (**C**HCH_2_), 79.4 (CH**C**H_2_), 105.7 (Py**C**_**2**_), 108.7 (Py**C**_**3**_), 118.2 (Py**C**_**4**_), 127.9 (2C, Ph**C**_**2**_), 129.2 (Py**C**_**1**_), 130 (2C, Ph**C**_**3**_), 134.9 (Ph**C**_**4**_), 138.0 (Ph**C**_**1**_); [Anal. Calcd. for C_13_H_14_N_2_O_2_: C, 67.81; H, 6.13; N, 12.17; found: C, 68.07; H, 5.95; N, 11.91]; LC/MS (ESI): M^+^, found 230.06, C_13_H_14_N_2_O_2_ requires 230.11; (*minor region-isomer* **9b**): IR (KBr): 3388, 1514, 1377, 717, 519 cm^−1^; ^1^H-NMR (CDCl_3_, 400 MHz) **δ** 2.25 (s, 1H, C**H**_3_), 4.54–4.79 (m, 4H, CHC**H**_2_), 4.79–4.82 (m, 2H, C**H**CH_2_), 5.86–5.94 (m, 2H, Ar**H**), 6.89–6.98 (m, 4H, Ar**H**), 6.98–7.09 (m, 4H, Ar**H**), 7.45 (s, 1H,N**H** of pyrrole); ^13^C-NMR (CDCl_3_, 100 MHz): **δ** 21.1 (**C**H_3_), 42.6 (**C**HCH_2_), 79.3 (CH**C**H_2_), 106.0 (Py**C**_**2**_), 106.4 (Py**C**_**2**_), 127.7 (Ph**C**_**2**_), 129.9 (Py**C**_**1**_), 129.9 (Ph**C**_**3**_), 134.7 (Ph**C**_**4**_), 137.9 (Ph**C**_**1**_); [Anal. Calcd. for C_22_H_23_N_3_O_4_: C, 67.16; H, 5.89; N, 10.68; found: C, 66.86; H, 6.13; N, 10.94]; LC/MS (ESI): M^+^, found 393.19, C_22_H_23_N_3_O_4_ requires 393.17.

#### 2.3.3. 2-(1-(4-Methoxyphenyl)-2-nitroethyl)-1H-pyrrole (Table 3, Entry 3, **8c**) and 2,5-Bis(1-(4-methoxy-phenyl)-2-nitroethyl)-1H-pyrrole (Table 3, Entry 3, **9c**)

Pyrrole **5** (100 mg, 0.86 mmol) and **β**-nitroolefin **2c** (154 mg, 0.86 mmol) in isopropanol (7 mL) were reacted in the presence of Feist's acid (**1**) (12 mg, 0.168 mmol, 10 mol%) according to **GP2**. The products were isolated by column chromatography on silica (EtOAc/hexane 0.5 : 9.5) yielded as oily liquid **8c **(major region-isomer) (177 mg, 0.72 mmol, 84%) and **9c** (minor region-isomer) as yellow oil (50 mg, 0.12 mmol, 14%). *Major region-isomer* **8c**: IR (KBr): 3388, 1542, 1378, 716, 584 cm^−1^; ^1^H-NMR (CDCl_3_, 400 MHz) **δ** 3.78 (s, 1H, OC**H**_3_), 4.69–4.80 (m, 1H, CHC**H**_2(a)_), 4.80–4.89 (m, 1H, CHC**H**_2(b)_), 4.90–5.01 (m, 1H, C**H**CH_2_), 6.06 (s, 1H, Ar**H**), 6.15–6.22 (m, 1H, Ar**H**), 6.67 (s, 1H. Ar**H**), 6.86 (d, 2H, *J* = 8.8 Hz, Ar**H**), 7.13 (d, 2H, *J* = 8.0 Hz, Ar**H**), 7.87 (s, 1H,N**H** of pyrrole); ^13^C-NMR (CDCl_3_, 100 MHz): **δ** 42.3 (**C**HCH_2_), 55.4 (O**C**H_3_), 79.5 (CH**C**H_2_), 105.6 (Py**C**_**2**_), 108.7 (Py**C**_**3**_), 114.6 (2C, Ph**C**_**3**_), 118.2 (Py**C**_**4**_), 129.1 (2C, Ph**C**_**2**_), 129.4 (Py**C**_**1**_), 130 (Ph**C**_**4**_), 159.4 (Ph**C**_**1**_); [Anal. Calcd. for C_13_H_14_N_2_O_3_: C, 63.40; H, 5.73; N, 11.38; found: C, 63.62; H, 5.58; N, 11.19]; LC/MS (ESI): M^+^, found 246.14, C_13_H_14_N_2_O_3_ requires 246.10; *minor region-isomer* **9c**: IR (KBr): 3388, 1514, 1377, 717, 519 cm^−1^; ^1^H-NMR (CDCl_3_, 400 MHz) **δ** 3.76 (s, 1H, C**H**_3_), 4.60–4.78 (m, 4H, CHC**H**_2_), 4.78–4.93 (m, 2H, C**H**CH_2_), 5.92–6.01 (m, 2H, Ar**H**), 6.80–6.84 (m, 4H, Ar**H**), 6.01–7.12 (m, 4H, Ar**H**), 7.54 (s, 1H,N**H** of pyrrole); ^13^C-NMR (CDCl_3_, 100 MHz): **δ** 41.2 (**C**HCH_2_), 55.4 (**C**H_3_), 79.4 (CH**C**H_2_), 105.9 (Py**C**_**2**_), 106.3 (Py**C**_**2**_), 114.6 (Ph**C**_**3**_), 128.96 (Ph**C**_**2**_), 129.02 (Py**C**_**1**_), 129.8 (Ph**C**_**1**_), 159.4 (Ph**C**_**4**_); [Anal. Calcd. for C_22_H_23_N_3_O_6_: C, 62.11; H, 5.45; N, 9.88; found: C, 62.27; H, 5.39; N, 10.13]; LC/MS (ESI): M^+^, found 425.13, C_22_H_23_N_3_O_6_ requires 425.16.

#### 2.3.4. 2-(1-(4-Chlorophenyl)-2-nitroethyl)-1H-pyrrole (Table 3, Entry 4, **8d**) and 2,5-Bis(1-(4-chlorophenyl)-2-nitroethyl)-1H-pyrrole (Table 3, Entry 4, **9d**)

Pyrrole **5** (100 mg, 0.86 mmol) and **β**-nitroolefin **2d** (157 mg, 0.86 mmol) in dry ethanol (7 mL) were reacted in the presence of Feist's acid (**1**) (12 mg, 0.168 mmol, 10 mol%) according to **GP2**. The products were isolated by column chromatography on silica (EtOAc/hexane 0.5 : 9.5) yielded white solid **8d **(major region-isomer) (140 mg, 0.56 mmol, 65%) and **9d** (minor region-isomer) as yellow oil (40 mg, 0.09 mmol, 11%). *Major region-isomer* **8d**: IR (KBr): 3379, 1740, 1545, 1377, 1092, 725, 512, 452 cm^−1^; ^1^H-NMR (CDCl_3_, 400 MHz) **δ** 4.66–4.83 (m, 2H, CHC**H**_2(a)_), 4.83–4.91 (m, 2H, CHC**H**_2(b)_), 4.91–5.06 (m, 1H, C**H**CH_2_), 6.07 (s, 1H, Ar**H**), 6.12–6.21 (m, 1H, Ar**H**), 6.70 (s, 1H. Ar**H**), 7.17 (d, 2H, *J* = 6.84 Hz, Ar**H**), 7.33 (d, 2H, *J* = 6.84, Hz, Ar**H**), 7.84 (s, 1H,N**H** of pyrrole); ^13^C-NMR (CDCl_3_, 100 MHz): **δ** 42.4 (**C**HCH_2_), 79.0 (CH**C**H_2_), 106.1 (Py**C**_**2**_), 108.9 (Py**C**_**3**_), 118.6 (Py**C**_**4**_), 128.4 (Ph**C**_**4**_), 129.4 (2C, Ph**C**_**2**_), 129.5 (2C, Ph**C**_**3**_), 134.2 (Py**C**_**1**_), 136.6 (Ph**C**_**1**_); [Anal. Calcd. for C_12_H_11_ClN_2_O_2_: C, 57.49; H, 4.42; N, 11.17; found: C, 57.36; H, 4.53; N, 11.01]; LC/MS (ESI): M^+^ & [M+2]^+^, found 250.08 & 252.03, C_12_H_11_ClN_2_O_2_ requires 250.05; *minor region-isomer* **9d**: IR (KBr): 3413, 1549, 1490, 1375, 1091, 726, 512 cm^−1^; ^1^H-NMR (CDCl_3_, 400 MHz) **δ** 4.61–4.82 (m, 4H, CHC**H**_2_), 4.82–5.03 (m, 2H, C**H**CH_2_), 5.93–6.02 (m, 2H, Ar**H**), 7.01–7.20 (m, 4H, Ar**H**), 7.20–7.39 (m, 4H, Ar**H**), 7.53 (s, 1H, N**H** of pyrrole); ^13^C-NMR (CDCl_3_, 100 MHz): **δ** 42.2 (**C**HCH_2_), 78.9 (CH**C**H_2_), 106.5 (Py**C**_**2**_), 106.9 (Py**C**_**2**_), 129.2 (Ph**C**_**3**_), 129.3 (Ph**C**_**2**_), 129.52 (Py**C**_**1**_), 134.3 (Ph**C**_**4**_), 136.2 (Ph**C**_**1**_); [Anal. Calcd. for C_20_H_17_Cl_2_N_3_O_4_: C, 55.31; H, 3.95; N, 9.68; found: C, 55.19; H, 4.14; N, 9.43]; LC/MS (ESI): M^+^ & [M+2]^+^, found 433.11 & 435.15, C_20_H_17_Cl_2_N_3_O_4_ requires 433.05.

#### 2.3.5. 2-(1-(4-Bromophenyl)-2-nitroethyl)-1H-pyrrole (Table 3, Entry 5, **8e**)

Pyrrole **5** (100 mg, 0.86 mmol) and **β**-nitroolefin **2e** (195 mg, 0.86 mmol) in isopropanol (7 mL) were reacted in the presence of Feist's acid (**1**) (12 mg, 0.168 mmol, 10 mol%) according to **GP2**. The product was isolated by column chromatography on silica (EtOAc/hexane 0.5 : 9.5) yielded as white solid **8e **(120 mg, 0.41 mmol, 47%). IR (KBr): 3376, 1543, 1377, 1007, 725, 510 cm^−1^; ^1^H-NMR (CDCl_3_, 400 MHz) **δ** 4.71–4.81 (m, 1H, CHC**H**_2(a)_), 4.81–4.90 (m, 1H, CHC**H**_2(b)_), 4.90–5.02 (m, 2H, C**H**CH_2_), 6.07 (s, 1H, Ar**H**), 6.12–6.22 (m, 1H, Ar**H**), 6.70 (s, 1H. Ar**H**), 7.11 (d, 2H, *J* = 8.8 Hz, Ar**H**), 7.48 (d, 2H, *J* = 8.8, Hz, Ar**H**), 7.86 (s, 1H, N**H** of pyrrole); ^13^C-NMR (CDCl_3_, 100 MHz): **δ** 42.5 (**C**HCH_2_), 79.0 (CH**C**H_2_), 106.1 (Py**C**_**2**_), 108.9 (Py**C**_**3**_), 118.6 (Py**C**_**4**_), 122.3 (Ph**C**_**4**_), 128.3 (Py**C**_**1**_), 129.7 (2C, Ph**C**_**2**_), 132.4 (Ph**C**_**3**_), 137.1 (Ph**C**_**1**_); [Anal. Calcd. for C_12_H_11_BrN_2_O_2_: C, 48.84; H, 3.76; N, 9.49; found: C, 49.07; H, 3.89; N, 9.35]; LC/MS (ESI): M^+^ & [M+2]^+^, found 294.01 & 296.05, C_12_H_11_BrN_2_O_2_ requires 294.0.

#### 2.3.6. 2-(2-Nitro-1-(4-nitrophenyl) ethyl)-1H-pyrrole (Table 3, Entry 6, **8f**)

Pyrrole **5** (100 mg, 0.8 mmol) and **β**-nitroolefin **2f** (167 mg, 0.86 mmol) in isopropanol (7 mL) were reacted in the presence of Feist's acid (**1**) (12 mg, 0.168 mmol, 10 mol%) according to **GP2**. The product was isolated by column chromatography on silica (EtOAc/hexane 0.5 : 9.5) yielded as oily liquid **8f** (224 mg, 0.86 mmol, 99.8%). IR (KBr): 3421, 1551, 1520, 1373, 1349, 784, 717 cm^−1^; ^1^H-NMR (CDCl_3_, 400 MHz) **δ** 4.93–5.05 (m, 2H, CHC**H**_2_), 5.51–5.57 (m, 1H, C**H**CH_2_), 6.12–6.19 (m, 2H, Ar**H**), 6.73 (s, 1H. Ar**H**), 7.31–7.48 (m, 2H, Ar**H**), 7.48–7.61 (m, 1H, Ar**H**), 7.81–7.93 (m, 1H, Ar**H**), 8.45 (s, 1H, N**H** of pyrrole); ^13^C-NMR (CDCl_3_, 100 MHz): **δ** 36.9 (**C**HCH_2_), 79.8 (CH**C**H_2_), 105.6 (Py**C**_**2**_), 108.8 (Py**C**_**3**_), 118.9 (Py**C**_**4**_), 127.8 (Py**C**_**3**_), 128.8 (2C, Ph**C**_**2**_), 129.4 (Py**C**_**1**_), 133.7 (Ph**C**_**4**_), 149.6 (Ph**C**_**1**_); [Anal. Calcd. for C_12_H_11_N_3_O_4_: C, 55.17; H, 4.24; N, 16.09; found: C, 55.36; H, 4.19; N, 15.93]; LC/MS (ESI): M^+^, found 261.15, C_12_H_11_N_3_O_4_ requires 261.07.

#### 2.3.7. 2-(1-(2,4-Dichlorophenyl)-2-nitroethyl)-1H-pyrrole (Table 3, Entry 7, **8g**)

Pyrrole **5** (100 mg, 0.86 mmol) and **β**-nitroolefin **2g** (187 mg, 0.86 mmol) in isopropanol (7 mL) were reacted in the presence of Feist's acid (**1**) (12 mg, 0.168 mmol, 10 mol%) according to **GP2**. The product was isolated by column chromatography on silica (EtOAc/hexane 1 : 9) yielded as oily liquid **8g** (245 mg, 0.86 mmol, 99.9%). IR (KBr): 3417, 1550, 1469, 1376, 1099, 797, 726 cm^−1^; ^1^H-NMR (CDCl_3_, 400 MHz) **δ** 4.71–4.98 (m, 2H, CHC**H**_2_), 5.32–5.49 (m, 1H, C**H**CH_2_), 6.06–6.23 (m, 2H, Ar**H**), 6.71 (s, 1H, Ar**H**), 6.94–7.10 (m, 1H, Ar**H**), 7.10–7.29 (m, 1H, Ar**H**), 7.31–7.58 (m, 1H, Ar**H**), 8.02 (s, 1H, N**H** of pyrrole); ^13^C-NMR (CDCl_3_, 100 MHz): **δ** 39.0 (**C**HCH_2_), 79.9 (CH**C**H_2_), 106.6 (Py**C**_**2**_), 108.9 (Py**C**_**3**_), 118.7 (Py**C**_**4**_), 127.4 (Py**C**_**1**_), 128.0 (Ph**C**_**6**_), 130.0 (Ph**C**_**5**_), 130.1 (Ph**C**_**3**_), 134.3–134.6 (Ph**C**_**1**_, Ph**C**_**2**_ & Ph**C**_**4**_); [Anal. Calcd. for C_12_H_10_Cl_2_N_2_O_2_: C, 50.55; H, 3.54; N, 9.82; found: C, 50.33; H, 3.69; N, 10.07]; LC/MS (ESI): M^+^, found 284.05, C_12_H_11_N_3_O_4_ requires 284.01.

#### 2.3.8. 2-(1-(2,6-Dichlorophenyl)-2-nitroethyl)-1H-pyrrole (Table 3, Entry 8, **8h**) and 2,5-Bis(1-(2,6-dichloro-phenyl)-2-nitroethyl)-1H-pyrrole (Table 3, Entry 8, **9h**)

Pyrrole **5** (100 mg, 0.86 mmol) and **β**-nitrostyrene **2h** (187 mg, 0.86 mmol) in isopropanol (7 mL) were reacted in the presence of Feist's acid (**1**) (12 mg, 0.168 mmol, 10 mol%) according to **GP2**. The products were isolated by column chromatography on silica (EtOAc/hexane 0.5 : 9.5) yielded yellow oil **8h **(major region-isomer) (220 mg, 0.77 mmol, 90%) and **9h** (minor region-isomer) as yellow oil (40 mg, 0.09 mmol, 9%). *Major region-isomer* **8h**: IR (KBr): 3439, 1550, 1430, 1374, 775, 720, 536 cm^−1^; ^1^H-NMR (CDCl_3_, 400 MHz) **δ** 5.21–5.30 (m, 1H, CHC**H**_2(a)_), 5.30–4.42 (m, 1H, CHC**H**_2(b)_), 5.90–6.01 (m, 1H, C**H**CH_2_), 6.09 (s, 1H, Ar**H**), 6.12–6.28 (m, 1H, Ar**H**), 6.66–6.75 (m, 1H. Ar**H**), 7.12–7.24 (m, 1H, Ar**H**), 7.28–7.48 (m, 2H, Ar**H**), 8.06 (s, 1H, N**H** of pyrrole); ^13^C-NMR (CDCl_3_, 100 MHz): **δ** 39.0 (**C**HCH_2_), 79.5 (CH**C**H_2_), 106.8 (Py**C**_**2**_), 108.7 (Py**C**_**3**_), 118.1 (Py**C**_**4**_), 124.4 (2C, Ph**C**_**3**_), 126.8 (Ph**C**_**4**_), 129.8 (2C, Ph**C**_**2**_), 133.8 (Py**C**_**1**_), 142.8 (Ph**C**_**1**_); [Anal. Calcd. for C_12_H_10_Cl_2_N_2_O_2_: C, 50.55; H, 3.54; N, 9.82; found: C, 50.45; H, 3.43; N, 9.74]; LC/MS (ESI): M^+^ & [M+2]^+^, found 284.05 & 286.11, C_12_H_10_Cl_2_N_2_O_2_ requires 284.01; *minor region-isomer* **9h**: IR (KBr): 3437, 1741, 1551, 1431, 1372, 1210, 773, 532 cm^−1^; ^1^H-NMR (CDCl_3_, 400 MHz) **δ** 5.14–5.32 (m, 4H, CHC**H**_2_), 5.83 (t, *J* = 6.6 Hz, 2H, C**H**CH_2_), 5.93–6.02 (m, 2H, Ar**H**), 7.12–7.23 (m, 2 h, Ar**H**), 7.26–7.42 (m, 4 h, Ar**H**), 8.16 (s, 1H, N**H** of pyrrole); ^13^C-NMR (CDCl_3_,100 MHz): **δ** 42.2 (**C**HCH_2_), 78.9 (CH**C**H_2_), 106.5 (Py**C**_**2**_), 106.9 (Py**C**_**2**_), 129.2 (Ph**C**_**3**_), 129.3 (Ph**C**_**2**_), 129.52 (Py**C**_**1**_), 134.3 (Ph**C**_**4**_), 136.2 (Ph**C**_**1**_ **)**; [Anal. Calcd. for C_20_H_15_Cl_4_N_3_O_4_: C, 47.74; H, 3.0; N, 8.35; found: C, 48.09; H, 3.11; N, 8.51]; LC/MS (ESI): M^+^ & [M+2]^+^, found 501.03 and 503.06, C_20_H_15_Cl_4_N_3_O_4_ requires 500.98.

#### 2.3.9. 2-(1-Ferrocenyl)-2-nitroethyl)-1H-pyrrole (Table 3, Entry 9, **8i**)

Pyrrole **5** (100 mg, 0.86 mmol) and **β**-nitroolefin **2g** (167 mg, 0.86 mmol) in dry ethanol (7 mL) were reacted in the presence of Feist's acid (**1**) (12 mg, 0.168 mmol, 10 mol%) according to **GP2**. The product was isolated by column chromatography on silica (EtOAc/hexane 0.5 : 9.5) dark red solid **8g** (51 mg, 18%). IR (KBr): 3358, 1740, 1551, 1410, 1251, 1219, 1044, 728, 488 cm^−1^; ^1^H-NMR (CDCl_3_, 400 MHz) **δ** 4.13 (s, 1H, protons of C*p*), 4.23 (s, 5H, protons of C*p*), 4.30–4.39 (m, 2H, protons of C*p*), 4.64 (s, 2H, m, 2H, protons of C*p*), 5.10–5.21 (m, 1H, C**H**CH_2_), 5.21–5.40 (m, 2H, CHC**H**_2_), 6.26–6.36 (m, 1H, Ar**H**), 6.81–6.90 (m, 1H, Ar**H**), 7.18 (m, 1H, Ar**H**), 7.79 (s, 1H, N**H** of pyrrole); ^13^C-NMR (CDCl_3_, 100 MHz): **δ** 29.8, 69.1, 70.0, 70.6, 72.3, 84.0, 111.6, 123.0, 125.2, 126.6; [Anal. Calcd. for C_16_H_16_FeN_2_O_2_: C, 59.28; H, 4.98; N, 8.64; found: C, 59.17; H, 5.07; N, 8.49]; LC/MS (ESI): M^+^, found 324.12, C_16_H_16_FeN_2_O_2_ requires 324.16.

## 3. Result and Discussion

**Scheme 1:**
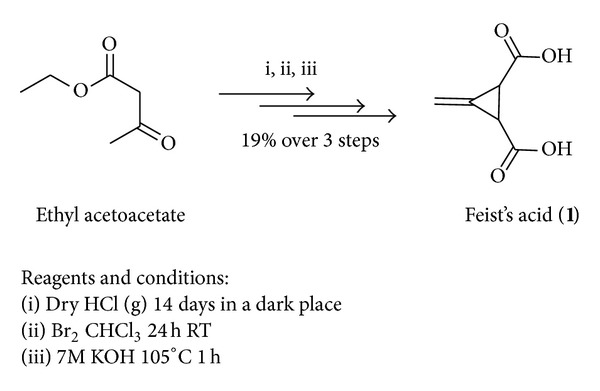
Feist's acid synthesis.

At the outset, synthetic strategies adopted in our work, for the preparation of Feist's acid (FA) (**1**) as hydrogen bond donor catalyst, has been outlined in Scheme 1. Feist's acid is commercially available but highly expensive therefore, it was prepared in our laboratory from a very cheap and readily available material ethyl acetoacetate with an overall yield of 19% in three steps, using the well-known method reported by Al-Majid et al. [50].

Initially, to evaluate the catalytic activity of Feist's acid for the Michel addition reaction, indole **4** and **β**-nitrostyrene **2a** have been chosen as model substrates. 5–20 mol% of FA (**1**) were used, aiming at screening the optimal conditions, and the results are summarized in Table 1.

By screening different solvents such as methanol, ethanol, isopropanol (^i^PrOH), toluene, benzene, xylene, acetonitrile, tetrahydrofuran (THF), and dichloromethane (CH_2_Cl_2_), using 10 mol% of Feist's acid, we found that most of the solvents produced good to excellent yields (86–96%) for the Michael addition reaction of indole **4** to **β**-nitrostyrene **2a**, affording the corresponding product **7a **at 50–60°C temperature within 24–42 h, (Table 1, entries 5–10, 14), while in case of ACN and MeOH, moderate yields were observed (49% and 62%), respectively (Table 1, entries 1, 4). But in case of other solvents like CH_2_Cl_2_ and THF, no products were formed (Table 1, entries 2, 3). Without using Feist's acid as catalyst, the reaction was carried out in ethanol at room temperature as well as at 60°C for 72 h; no product formation was observed at all (Table 1, entries 11, 12). But with the use 5 mol% catalyst in ethanol at 50°C for 60 h, 59% yield was observed (Table 1, entry 13). The yields of product were improved remarkably from 59% to 98% by increasing the loading of catalyst from 5 to 20 mol% in ethanol (Table 1, entries 13, 14). It is noteworthy to mention that solvents like DMF and AcOH with 10% catalyst produced very good results in 42 h and 18 h accordingly (Table 1, entries 5, 7). But due to the work-up problem these solvents cause they have been discarded from further reaction optimization. Xylene, toluene, and benzene also have been discarded for their high boiling point as compared to ethanol. The optimized procedure for Michael addition of indole to nitroolefin was found to be as follows: the mixture of indole **4** (0.43 mmol), **β**-nitrostyrene **2a** (0.43 mmol), and FA (Feist's acid, 10 mol%) was heated at 50°C in ethanol for 42 h according to general procedure **GP1**.

To illustrate the generality of this Michael addition reaction of indole **4**, with various nitroolefins **2(a–i)**, sixteen examples were carried out catalyzed by Feist's acid with 10 mol% and 20 mol% in ethanol at 50°C temperature and, the results are shown in Table 2.

The exclusive 3-substituted indole derivatives **7a–i** were obtained in good to excellent yields (46–97%) with the use of 10 mol% of catalyst at 50°C temperature in 48 hours (Table 2, entries 1–13). The reaction of indole (**4**) with nitroolefins **2(e–h)** bearing 4-bromophenyl, 2,4-dichlorophenyl, 2,6-dichlorophenyl, and 4-nitrophenyl produced the corresponding Michael adducts **7(e–h)** with poor to moderate yield (49%, 55%, 63%, and 62%), respectively (Table 2, entries 5, 7, 9, and 11). The poor reactivity of these nitroolefins could be inferred due to their less solubility and bulkier environment. But the chemical yields were increased dramatically to 87%, 70%, 97%, and 75%, respectively, when the loading of catalyst were increased from10–20 mol% and the reactions were run for 72 hours (Table 2, entries 6^c^, 8^c^, 10^c^, 12^c^). In contrast, the nitroolefin bearing phenyl, tolyl, and 4-chlorophenyl, afforded the corresponding Michael products in excellent yield (97%) (Table 2, entries 1, 2 and 4), while in the case of nitroolefin with 4-methoxyphenyl, the yield was slightly lower (84%) (Table 2, entry 3), which was probably attributed to the steric effect of the 4-methoxyphenyl group.

On the basis of the above results obtained for Michael addition reaction of indole with the derivatives of **β**-nitrostyrenes, the reaction was extended to pyrrole, and it was found that Feist's acid (FA) can also efficiently catalyze the reaction of pyrrole with different **β**-nitroolefins in isopropanol, affording 2-substituted pyrrole and in some cases both 2-substituted and 2,4-disubstituted pyrrole derivatives in good to excellent yields; the results are summarized in Table 3.

The Michael addition of pyrrole **5** to nitroolefins **2(a–i)** catalyzed by FA (10 mol%) in isopropanol at 50°C for 20–50 h afforded 2-substituted pyrrole **8(a–i)** as major region-isomer (Table 3, entries 1–9), with some 2,4-disubstituted pyrrole derivatives **9(a–d)** and **9h** (Table 3, entries 1–4, 8), which also showed good regioselectivity of pyrrole at the 2-position. The reaction of pyrrole with nitroolefins bearing phenyl, tolyl, 4-methoxyphenyl, 4-chloroophenyl and, 24-dichlorophenyl **2(a–d)** and **2h** afforded major region-isomer 2-substituted pyrrole **8(a–d)** and **8h** (81%, 50%, 84%, 65%, and 90%) with some minor region-isomer 2,6-disubstituted pyrrole **9(a–d)** and **9h** (13%, 21%, 13%, 11%, and 9%) in 50 h, 24 h, 44 h, 20 h, and 44 h, respectively (Table 3, entries 1–4, 8). But 2-substituted Michael adducts **8(e–g)** and **8i** were formed exclusively with 48%, 99.8%, 99.9%, and 18%, when nitroolefins bearing 4-bromophenyl, 4-nitrophenyl, 2,4-dichlorophenyl, and ferrocene groups reacted with pyrrole in 20 h and 44 h, accordingly (Table 3, entries 5–7, 9). Obviously the best results were found in the case of nitroolefin **2f** and **2g** producing their corresponding adducts **8f** and **8g** with excellent yields 99.8 and 99.9%, respectively (Table 3, entries 6, 7). On the other hand, poor yields (**8e** and **8i **with 48% and 18%) were observed corresponding to the nitroolefins **2e** and **2i**. The reason for this low yield could be attributed to the poor solubility of **2e** and **2i**.

**Figure 1:**
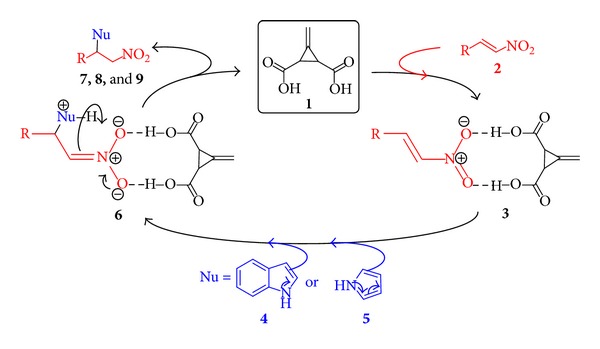
Activation of nitrostyrene via hydrogen bonding mechanism by Feist's acid catalysis.

We reasoned that, the catalytic cycle would begin with activation of an electrophile, such as **β**-nitrostyrenes (**2a–i**), with Feist's acid (**1**) to afford intermediate **3** (Figure 1). Addition of nucleophiles (**4** and **5**), to the intermediate **3** give rise to another intermediate **6** followed by proton transfer and release of the catalyst (**1**), would complete the cycle and generate the desired products **7a–i**, **8a–i**, **9a–d** and **9h** corresponding to the substrate **4** and **5 **(Tables 2 and 3). All the analytical data are available in the Supplementary Material available online at http//dox.doi.org/10.1155/2014/649197.

## 4. General Procedure for Antimicrobial Activity 

Chemical compounds were individually tested against a panel of Gram-positive and Gram-negative bacterial pathogens. Antimicrobial tests were carried out by the agar well diffusion method using 100 mL of suspension containing 1 × 10^8^ CFU/mL of pathological tested bacteria, 1 × 10^6^ CFU/mL of yeast and 1 × 10^4^ spore/mL of fungi spread on nutrient agar (NA), Sabouraud dextrose agar (SDA), and potato dextrose agar (PDA) medium, respectively. After the media had cooled and solidified, wells (10 mm in diameter) were made in the solidified agar and loaded with 100 mL of tested compound solution prepared by dissolving 100 mg of the chemical compound in one mL of dimethyl sulfoxide (DMSO). The inculcated plates were then incubated for 24 h at 37°C for bacteria and 48 h at 28°C for fungi. Negative controls were prepared using DMSO employed for dissolving the tested compound. Ciprofloxacin (50 mg/mL) and Ketoconazole (50 mg/mL) were used as standard for antibacterial and antifungal activity, respectively. After incubation time, antimicrobial activity was evaluated by measuring the zone of inhibition against the test organisms and compared with that of the standard. The observed zone of inhibition is presented in Table 1. Antimicrobial activities were expressed as inhibition diameter zones in millimeters (mm) as follows: N.A. (no activity) ≤ 4 mm; + (weak) = 5–9 mm; ++ (moderate) = 10–15 mm; +++ (strong) = 16–20 mm, and ++++ (very strong) ≥ 21 mm. The experiment was carried out in triplicate and the average zone of inhibition was calculated.

### 4.1. Antimicrobial Activity

A sample of some synthesized compounds (**7b**, **7d**, **7e**, **7f** and **8e**, **8f**, **8g**) has been subjected to antimicrobial activity studies including Gram-positive bacteria (*Bacillus subtilis* and *Staphylococcus aureus*), Gram-negative bacteria (*Pseudomonas Aeruginosa* and *Escherichia coli*), and fungi (*Candida albicans*). Antimicrobial tests were carried out by the agar well diffusion method. When compared to the standard drug Ciprofloxacin, it was seen that compounds **7b**, **7d**, **7e**, and **7f** with frame structure of indole moiety showed an inhibition effect against *Bacillus subtilis* ATCC 10400 (Table 4). On the other hand, **8e**, **8g** with frame structure of pyrrole moiety showed effect against *Bacillus subtilis* ATCC 10400. Not worthy to mention that **8f** showed potent inhibition against the same Gram-positive bacteria *Bacillus subtilis* ATCC 10400 compared with standard Ciprofloxacin (Table 4). Interestingly, **8e**, **8g** with frame structure of pyrrole moiety showed antifungal activity against *Candida albicans *ATTCC-10231, while **8f **showed potent inhibition against the same fungi *Candida albicans* ATTCC-1023 compared to standard drug Ketoconazole. Nevertheless, **7b**, **7d**, **7e**, **7f**, **8e**, **8f**, and **8g** were not active against *S. aureus* ATCC 29213, *E. coli* ATCC-35218, and *P. aeruginosa* ATCC 29336. The results obtained are summarized in Table 4 [59].

## 5. Conclusion

In summary, Feist's acid has been introduced as a new class of hydrogen bond donor catalysts for the activation of nitroalkene in conjugate addition reactions. This study includes the original report of Feist's acid catalysis of Michel addition of indole and pyrrole to a variety of nitroolefins. All the synthesized indole and pyrrole derivatives have be screened for antimicrobial activity (MIC determination) in our laboratory and the results are reported here in. Investigations surrounding the potential associated with Feist's acid catalysis, including the development of enantioselective variants, as a new tool for organic synthesis are under progress in our laboratory.

## Supplementary Material

Scan copies of ^1^H and ^13^C spectra of the Michael adducts **7a-i, 8a-I, 9a-d, & 9h**

## Figures and Tables

**Table 1:** Condition optimization of Feist's acid catalysis in the addition of indole^a^ (**4**) to **β**-nitro-styrene (**2a**).

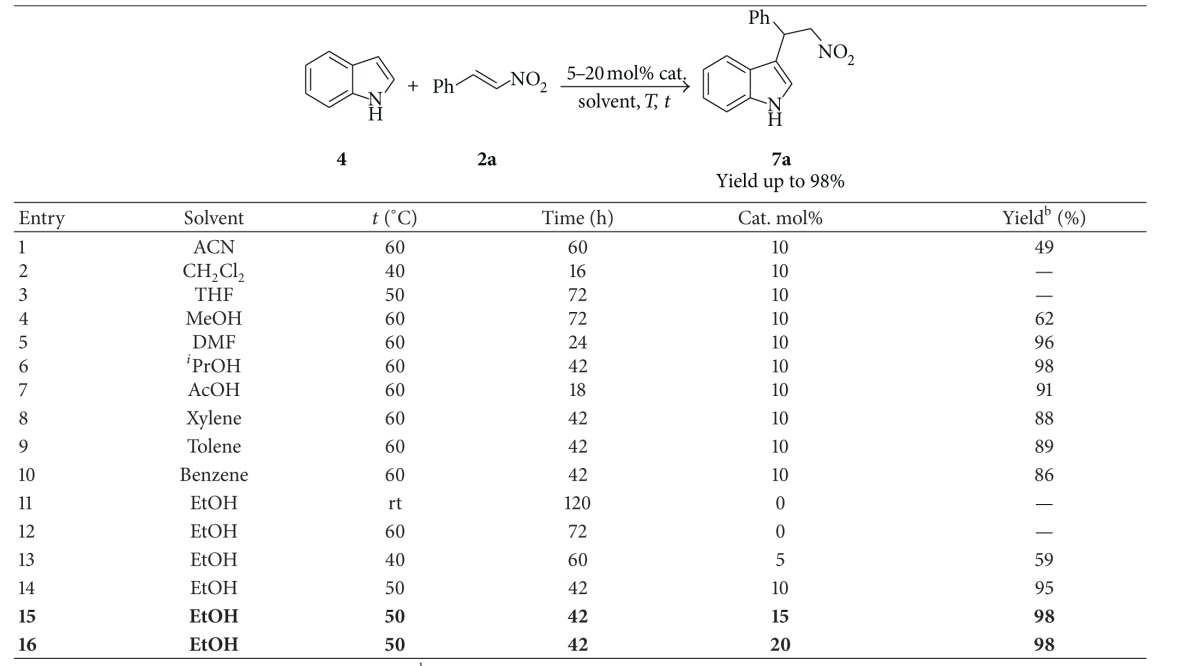

^a^The reactions were performed on an 0.425 mmol scale; ^b^the isolated yield after column purification.

**Table 2:** Michael addition of indole (**4**) to nitroolefins **2(a–i)** catalyzed by Feist's acid.

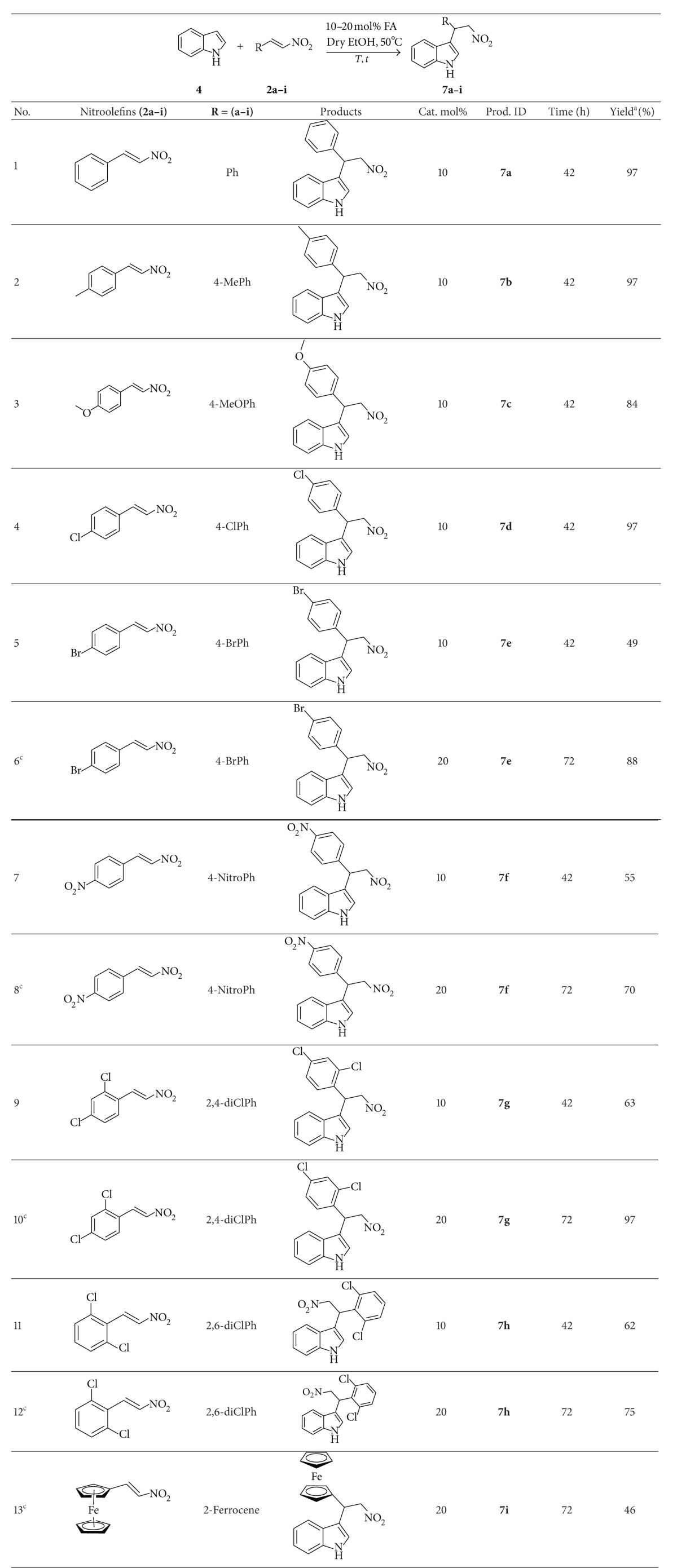

^a^The reactions were performed on 0.425 mmol scale; ^b^the isolated yield after column purification; ^c^20 mol% catalyst was used and reactions were run for 72 hours.

**Table 3:** Feist's acid catalysis substrate scope^a^, pyrrole **5**.

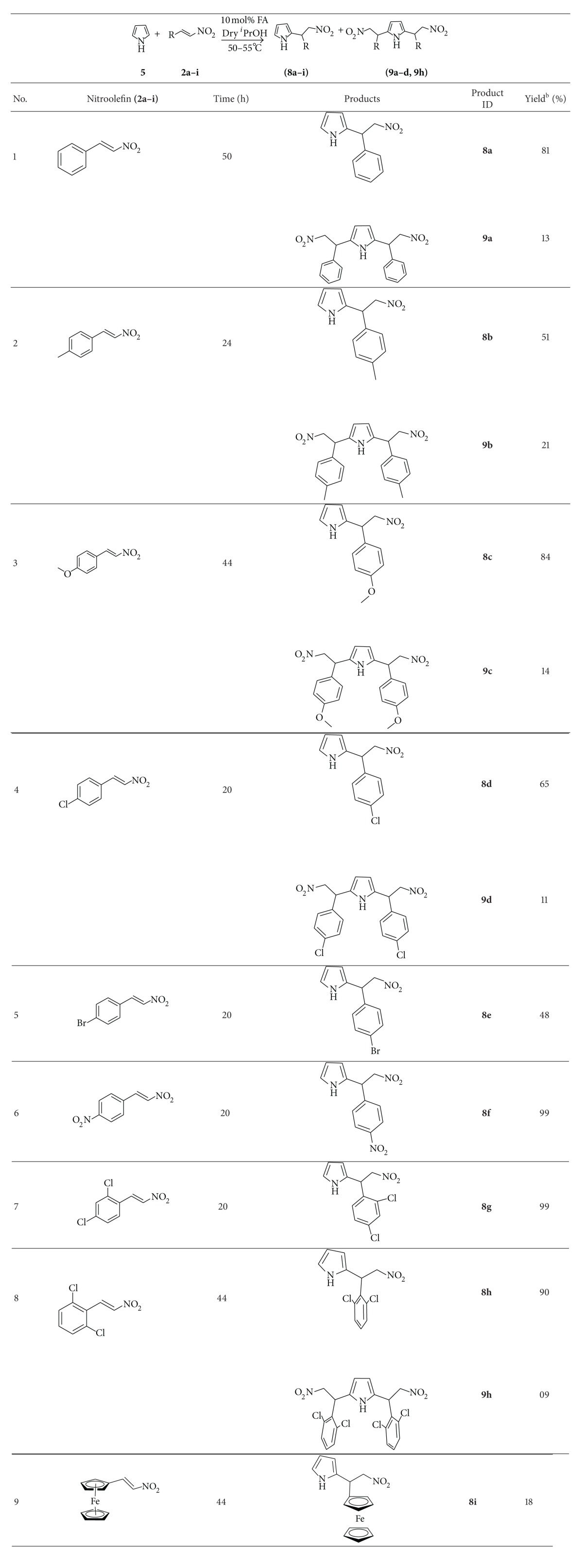

^a^The reactions were performed on 0.86 mmol scale; ^b^the isolated yield after column purification.

**Table 4:** Antimicrobial activity of the newly synthesized compounds against the pathological strains based on well diffusion assay^a^.

Comp. no.	Gram-positive bacteria		Gram-negative bacteria		Fungi
	*Staphylococcus aureus* ATTCC-29213	*Bacilils subtilis *ATTCC-10400	*Escherichia coli *ATTCC-35218	*Pseudomonas aeruginosa* ATTCC-29336	*Candida albicans *ATTCC-10231
**7b**	N.A.	++	N.A.	N.A.	N.A.
**7d**	N.A.	++	N.A.	N.A.	N.A.
**7e**	N.A.	++	N.A.	N.A.	N.A.
**7f**	N.A.	++	N.A.	N.A.	N.A.
**7g**	N.A.	++	N.A.	N.A.	N.A.
**8e**	N.A.	++	N.A.	N.A.	+++
**8f**	N.A.	++++	N.A.	N.A.	++++
**8g**	N.A.	++	N.A.	N.A.	++
**Ciprofloxacin**	+++	++++	++++	++++	N.A.
**Ketoconazole**	N.A.	N.A.	N.A.	N.A.	++++

^a^Antimicrobial activities were expressed as inhibition diameter zones in millimeters (mm) as follows: N.A. (no activity) ≤ 4 mm; + (weak) = 5–9 mm; ++ (moderate) = 10–15 mm; +++ (strong) = 16–20 mm; and ++++ (very strong) ≥ 21 mm. The experiment was carried out in triplicate and the average zone of inhibition was calculated.
