# Supplementary material for: Facile and Promising Method for Michael Addition of Indole and Pyrrole to Electron-Deficient trans-β-Nitroolefins Catalyzed by a Hydrogen Bond Donor Catalyst Feist's Acid and Preliminary Study of Antimicrobial Activity
Source: ScientificWorldJournal. 2014 Jan 16;2014:649197. doi: 10.1155/2014/649197 (PMC3915498; doi:10.1155/2014/649197)

Facile and promising method for Michael addition of indole and pyrrole to electron deficient trans-β-nitroolefins catalyzed by a hydrogen bond donor catalyst Feist's acid; and Preliminary study of anti-microbial activity

A. M. A. Al Majid, M. S. Islam*, A. Barakat, M. H.M. Al-Agamy, Mu.Naushad

Department of Chemistry, Faculty of Science, King Saud University, P.O. Box 2455, Riyadh 11451, Saudi Arabia

List of content:

Experimentals: General, Materials, Instrumentation……………………………………………………………………………...........*1*

Scan copies of ^{1}*H and* ^{13}*C spectra of the Michael adducts* ***7a-i, 8a-I, 9a-d, & 9h*** ………..*2 - 47*

Generals:

Glassware was oven-dried overnight at 120 ºC before use. Reactions were performed under an inert atmosphere using an argon filled glove box and standard Schlenk-line techniques. All the reactions were monitored by TLC analysis using Merck Silica Gel 60 F-254 thin layer plates. Column chromatography was performed on silica gel 100-200 mesh. **Materials:** Petroleum ether (PE), hexane and ethyl acetate for column chromatography were distilled prior to use. CH_2Cl_2, EtOH were distilled from P_2O_5 and Mg respectively and stored on 4Å molecular sieves. Tetrahydrofuran, benzene, toluene were distilled from sodium benzophenone ketyl. Acetonitrile and dimethyformamide were dried by distillation over calcium hydride. Nitroolefins **2a** – **i** were prepared according to procedures reported in literature.[1] **Instrumentation:** NMR spectra were recorded with a Jeol spectrometer at 400 MHz (^{1}H-NMR) and 100 MHz (^{13}C-NMR.). The chemical shifts (δ in ppm) were reported down field from tetramethylsilane (TMS, δ scale) with the deuterated solvent resonance referenced as internal standard. Elemental analyses were performed on a Perkin Elmer 2400 Elemental Analyzer. IR spectra were obtained using FTIR-800 Model. Mass spectrometric analysis was conducted by using ESI mode on AGILENT Technologies 6410-triple quad LC/MS instrument.

Reference:

1. N. A. Lange andW. E. Hambourger, "Condensation of aromatic aldehydes with nitromethane in the presence of alcoholic sodium hydroxide," *Journal of the American Chemical Society*, vol. 53, no. 10, pp. 3865–3867, 1931.

Compound 7a: ^{1}H-NMR

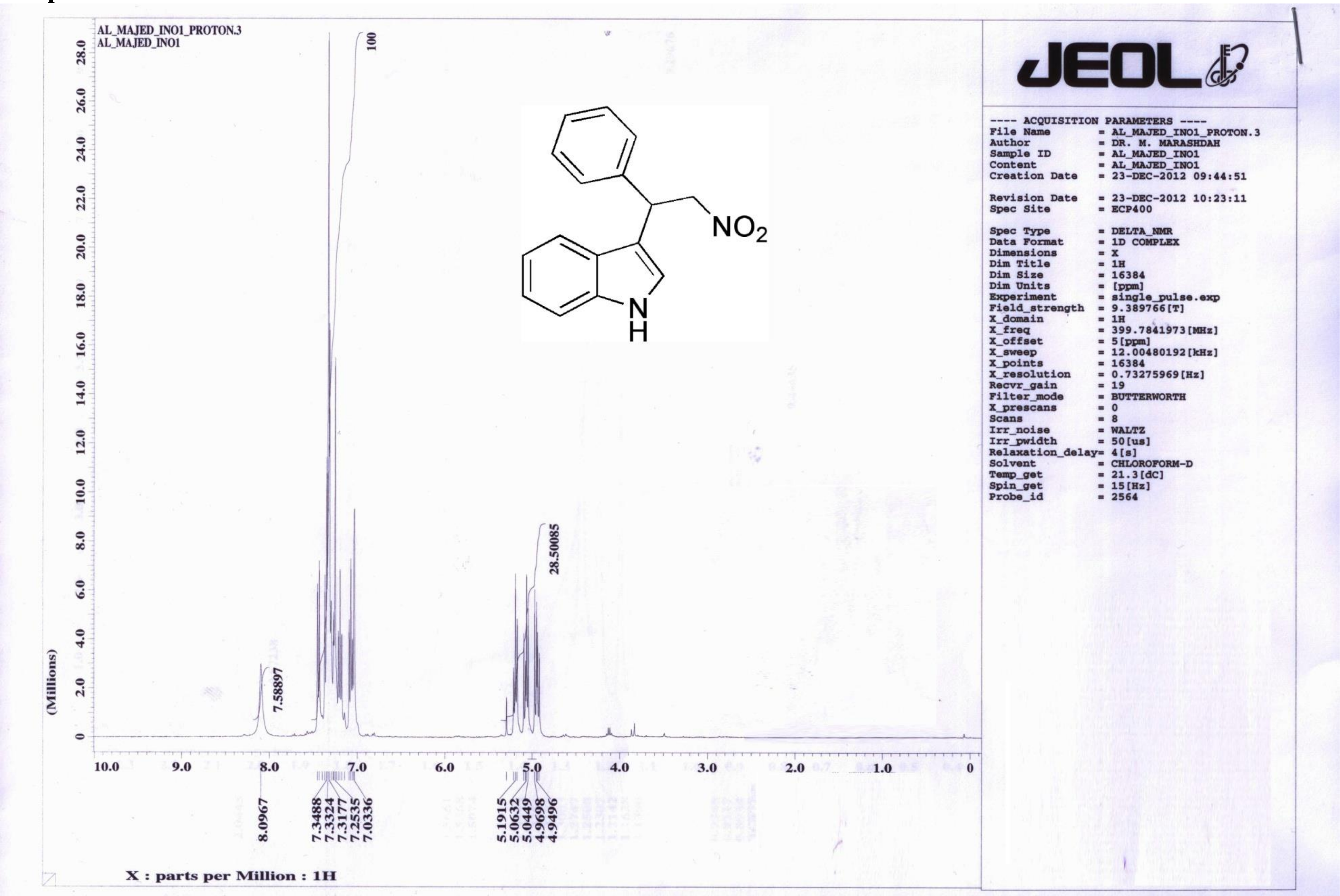

Compound 7a: ^{13}C-NMR

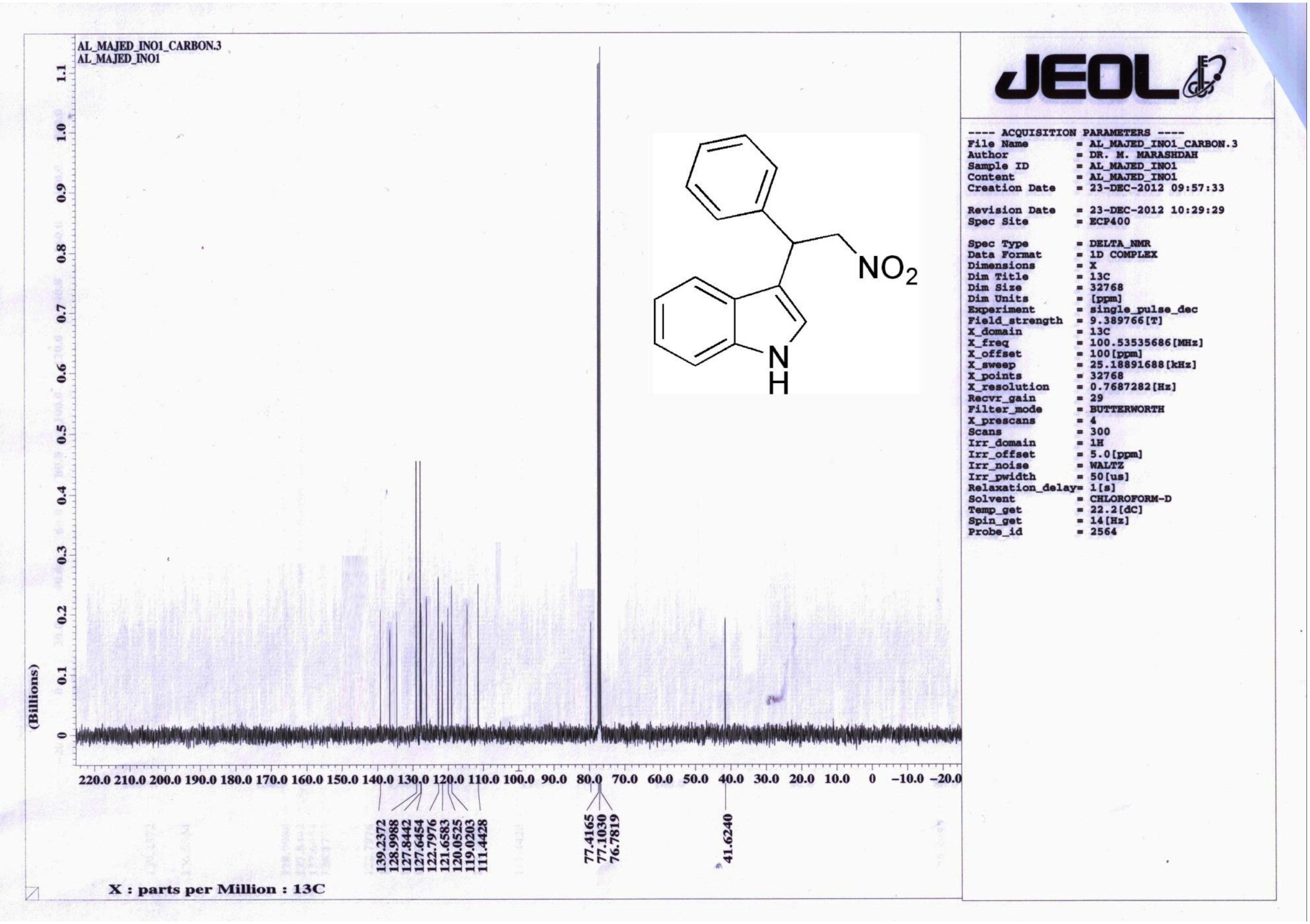

Compound 7b: ^{1}H-NMR

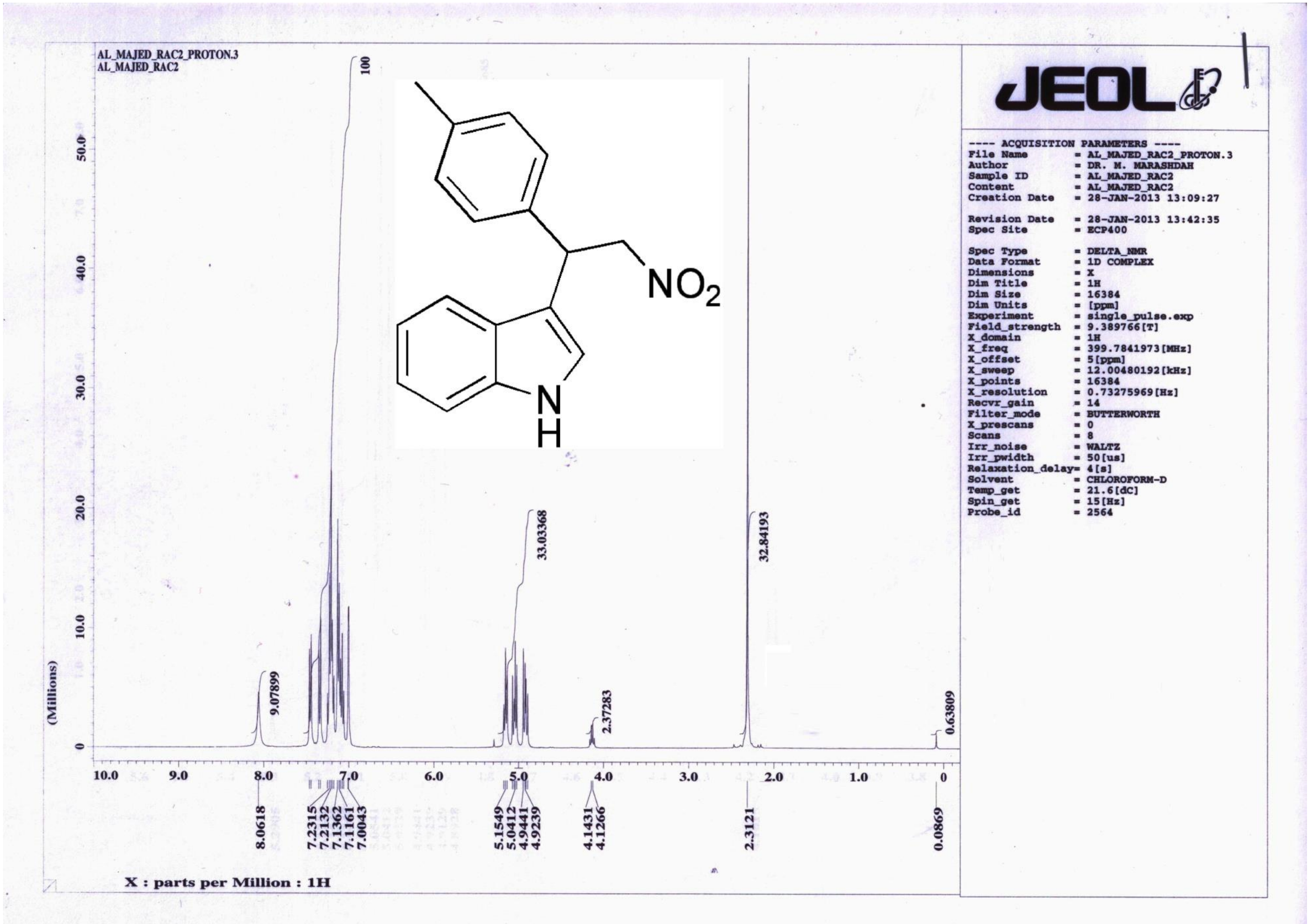

Compound 7b: ^{13}C-NMR

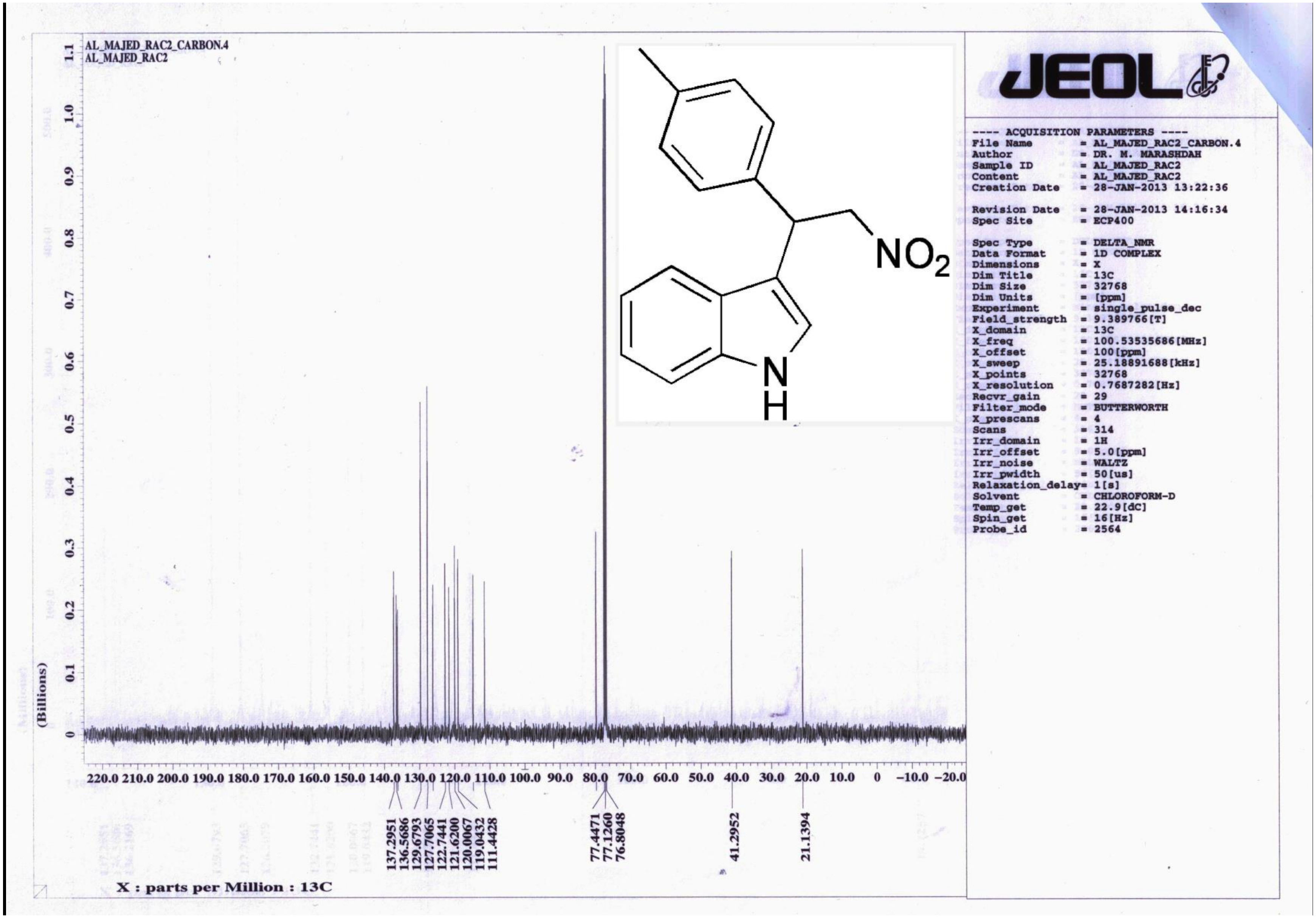

Compound 7c: ^{1}H-NMR

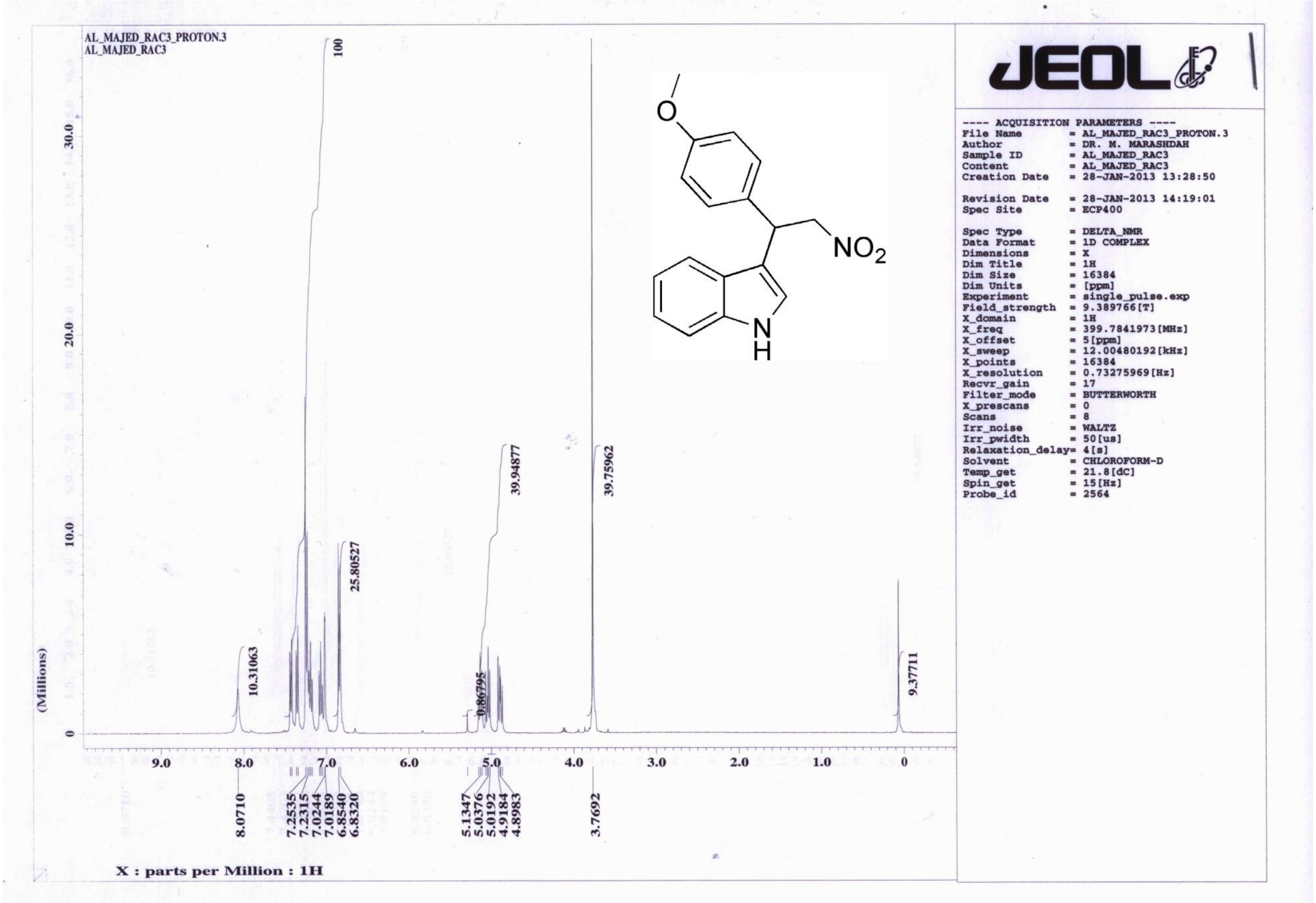

Compound 7c: ^{13}C-NMR

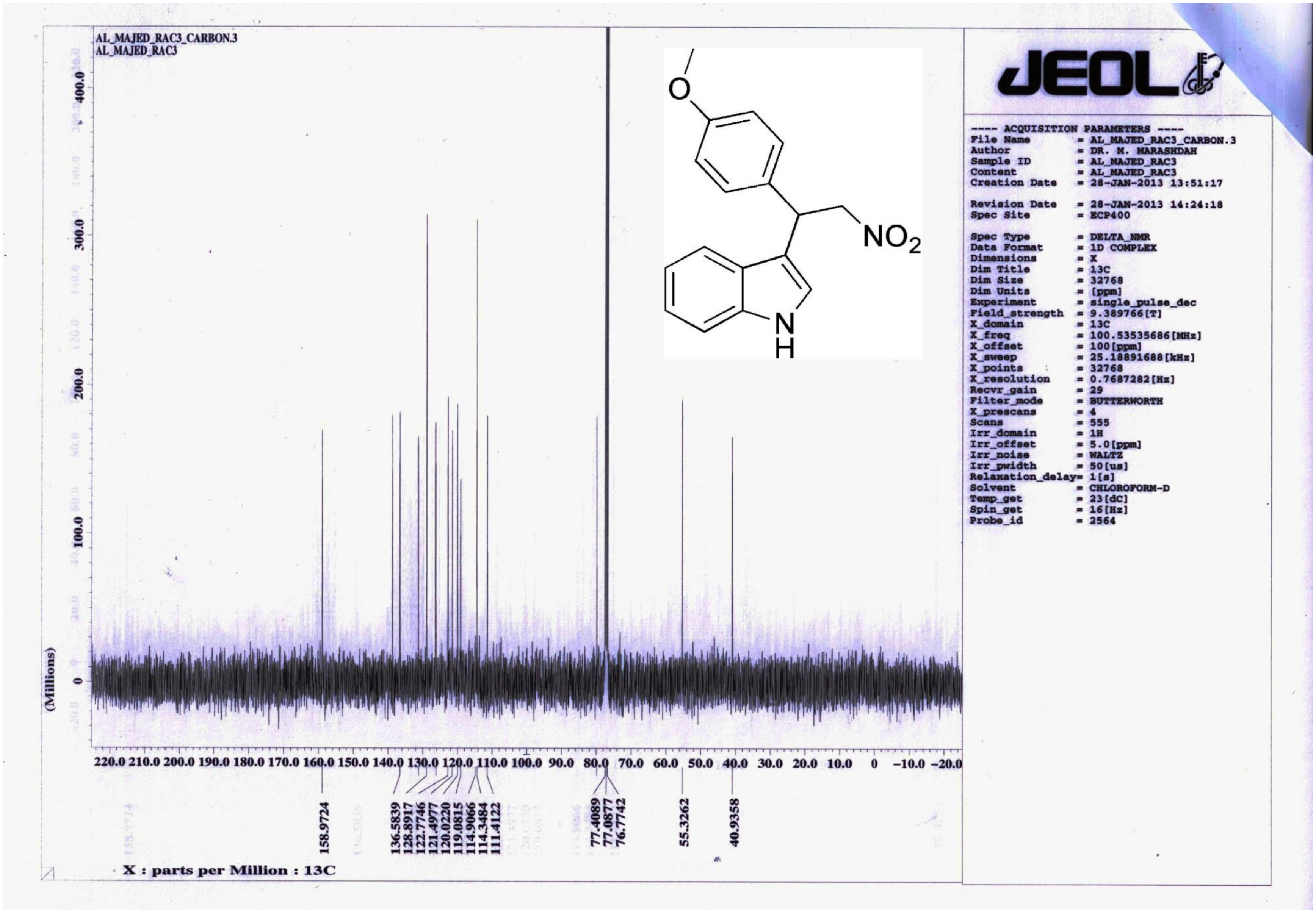

Compound 7d: ^{1}H-NMR

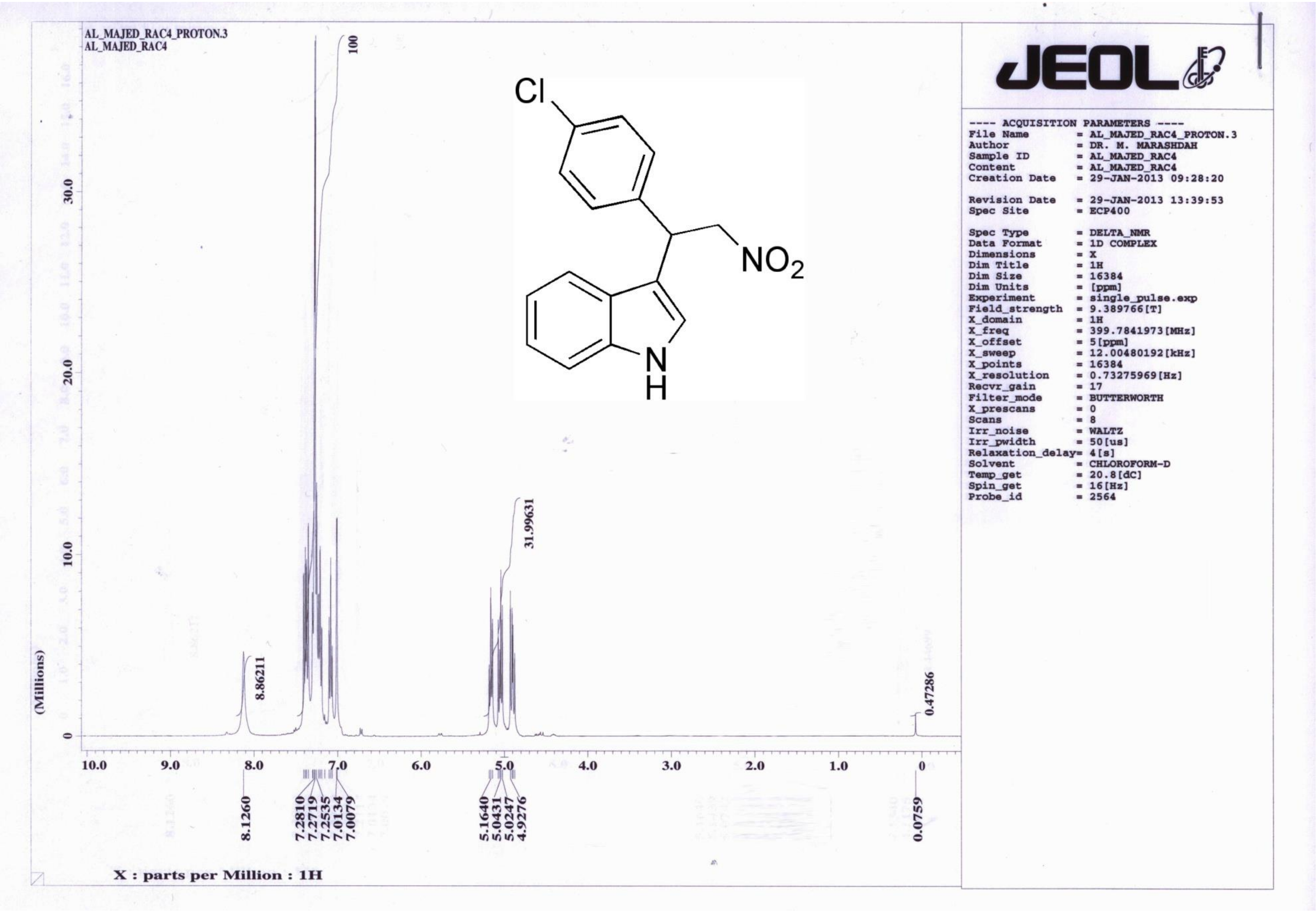

Compound 7d: ^{13}C-NMR

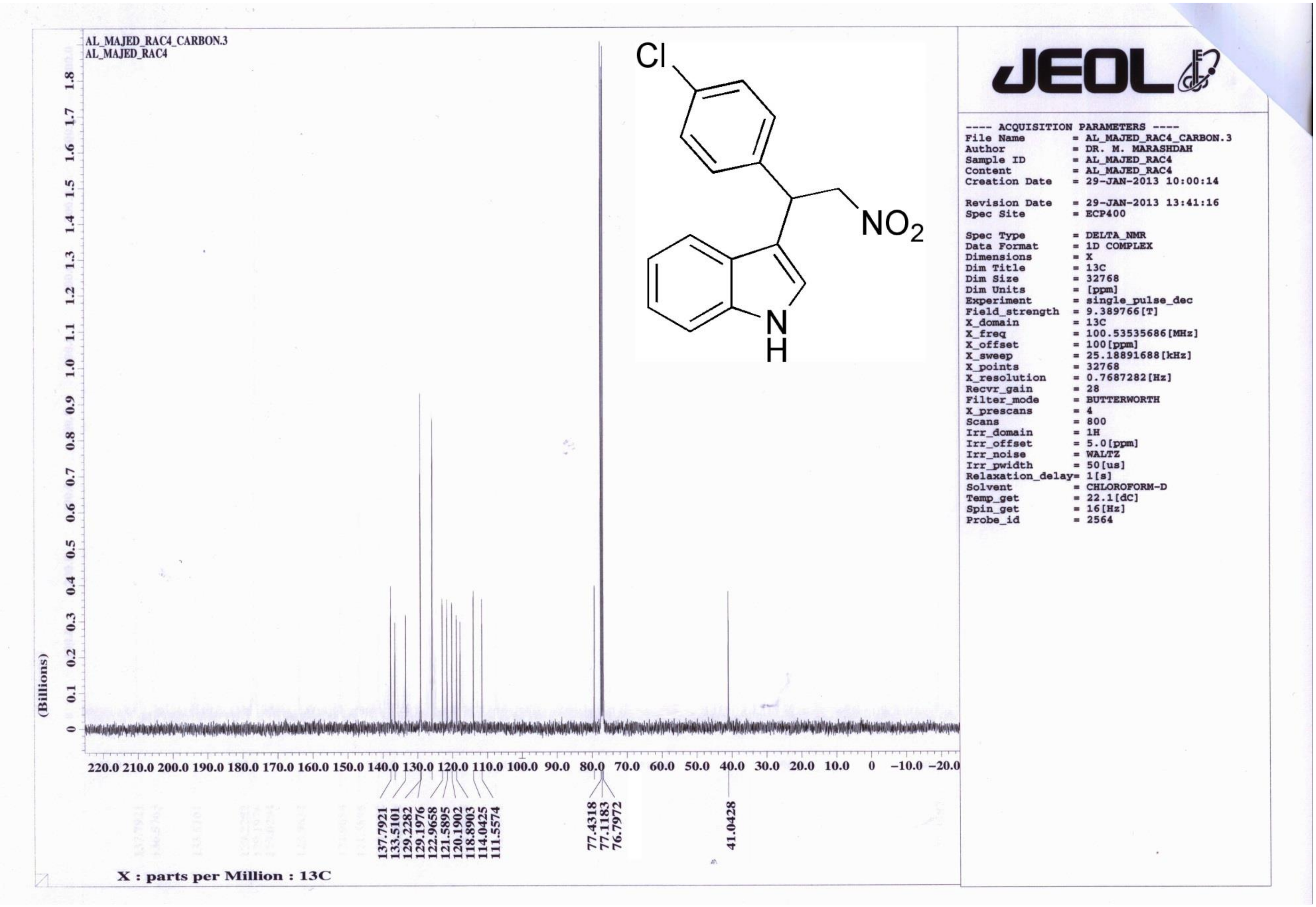

Compound 7e: ^{1}H-NMR

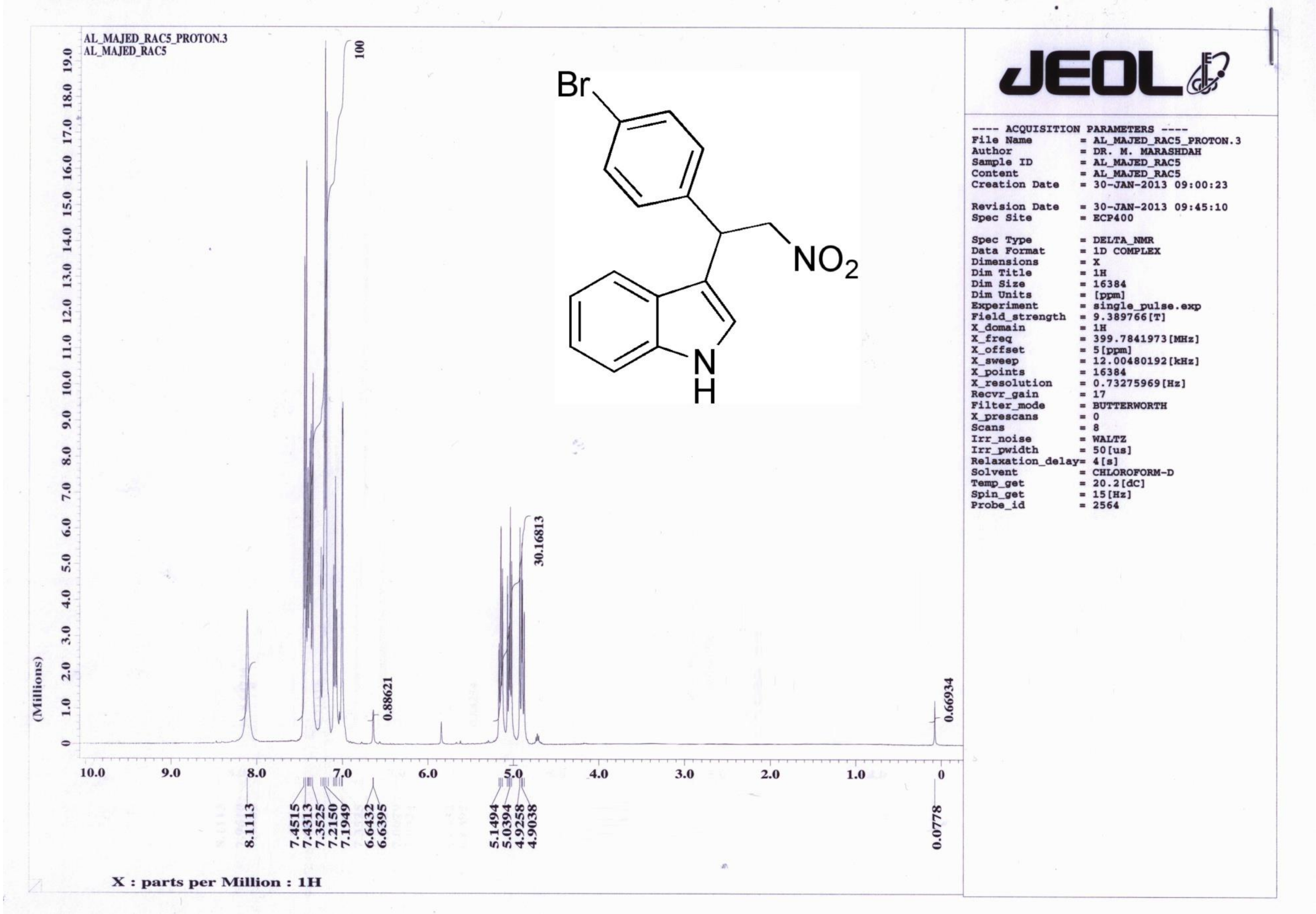

Compound 7e: ^{13}C-NMR

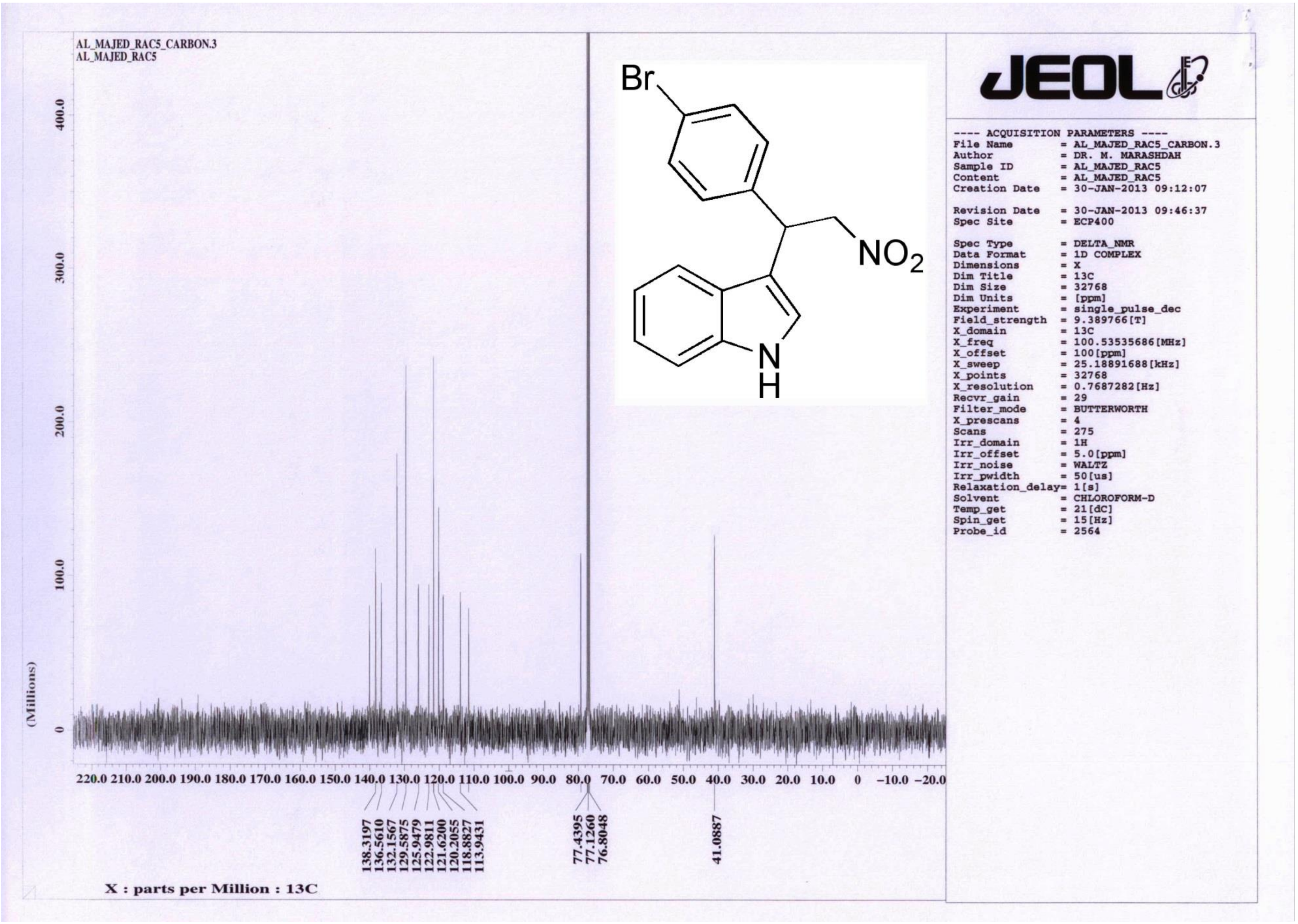

Compound 7f: ^{1}H-NMR

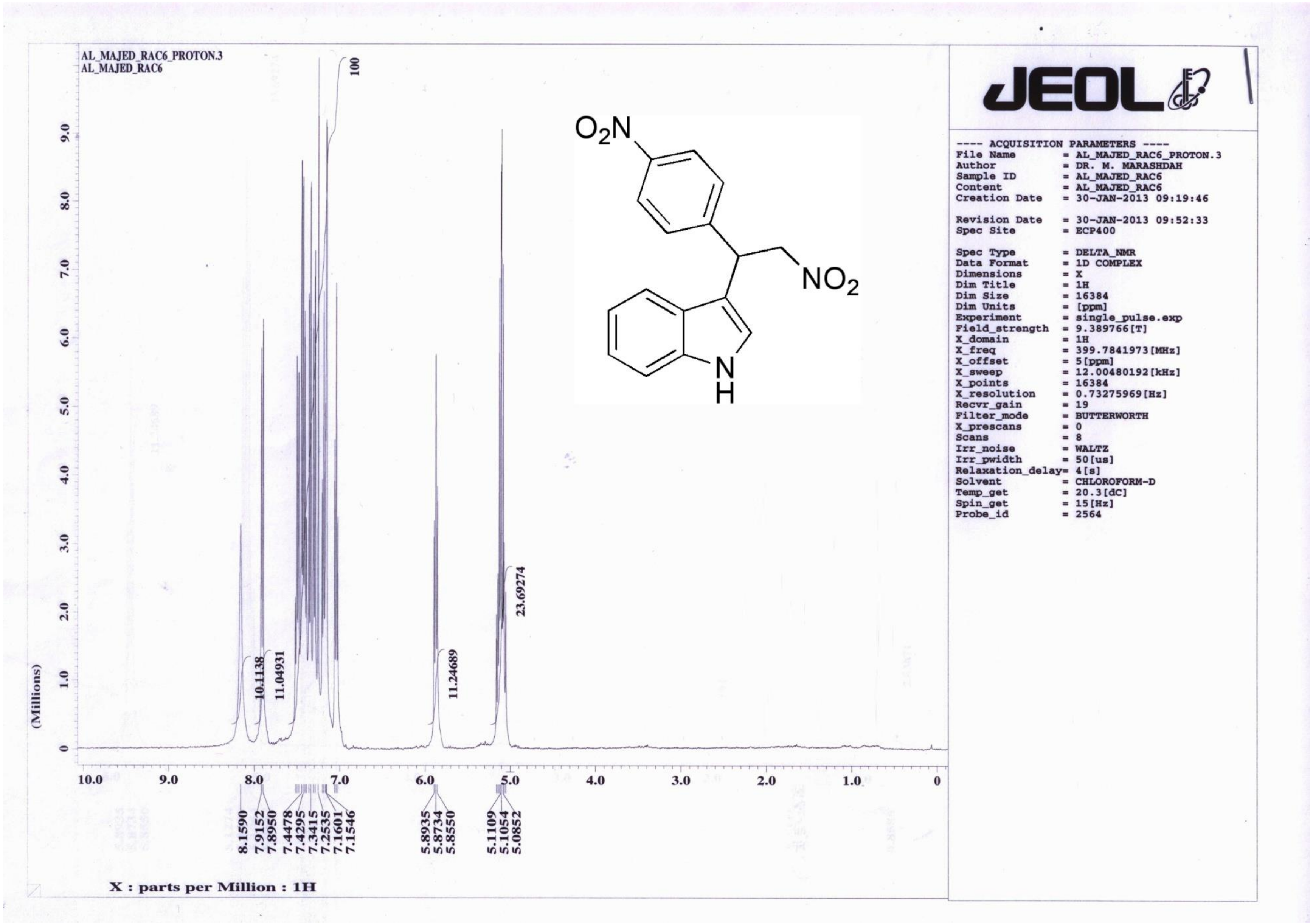

Compound 7f: ^{13}C-NMR

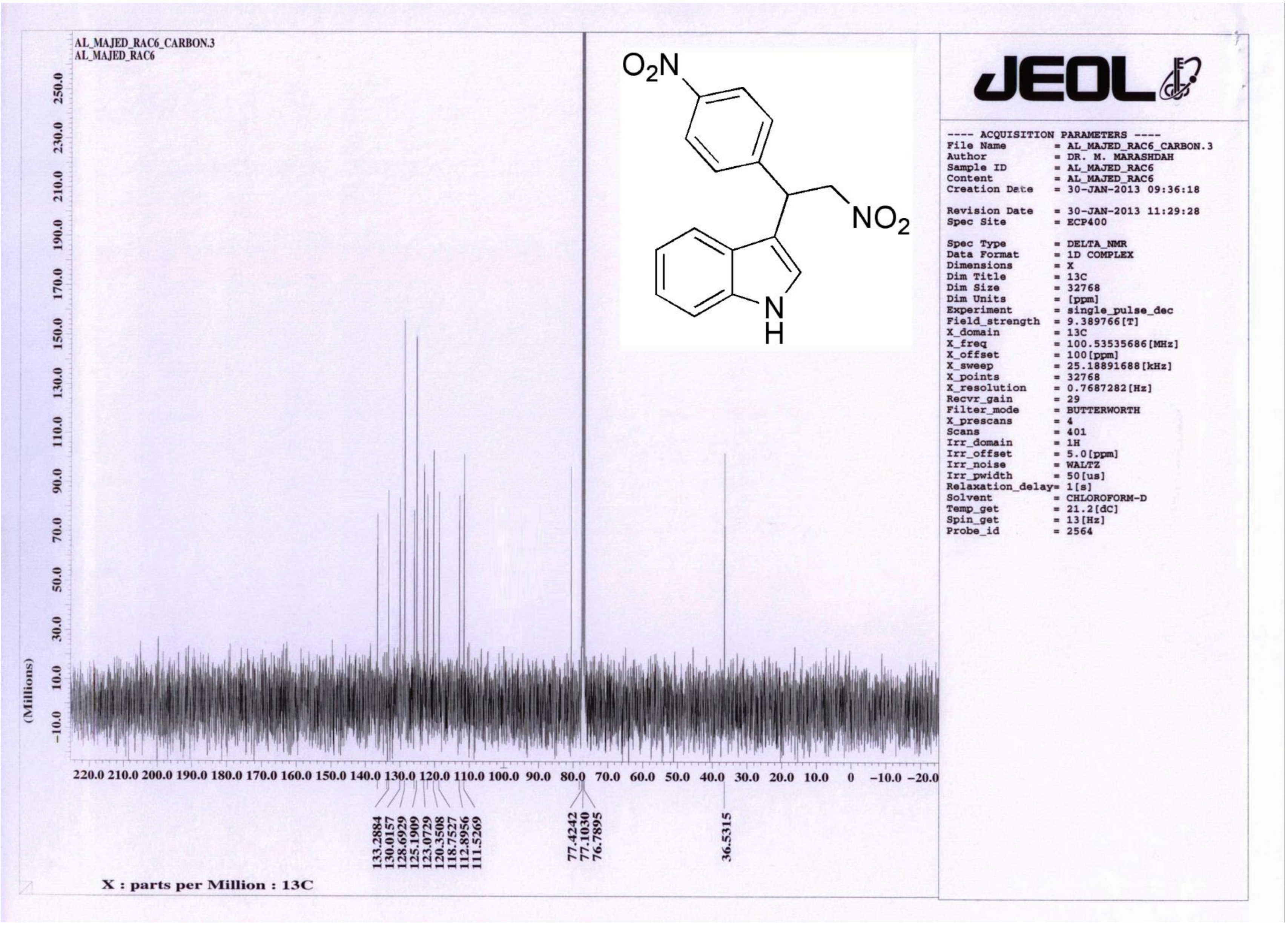

Compound 7g: ^{1}H-NMR

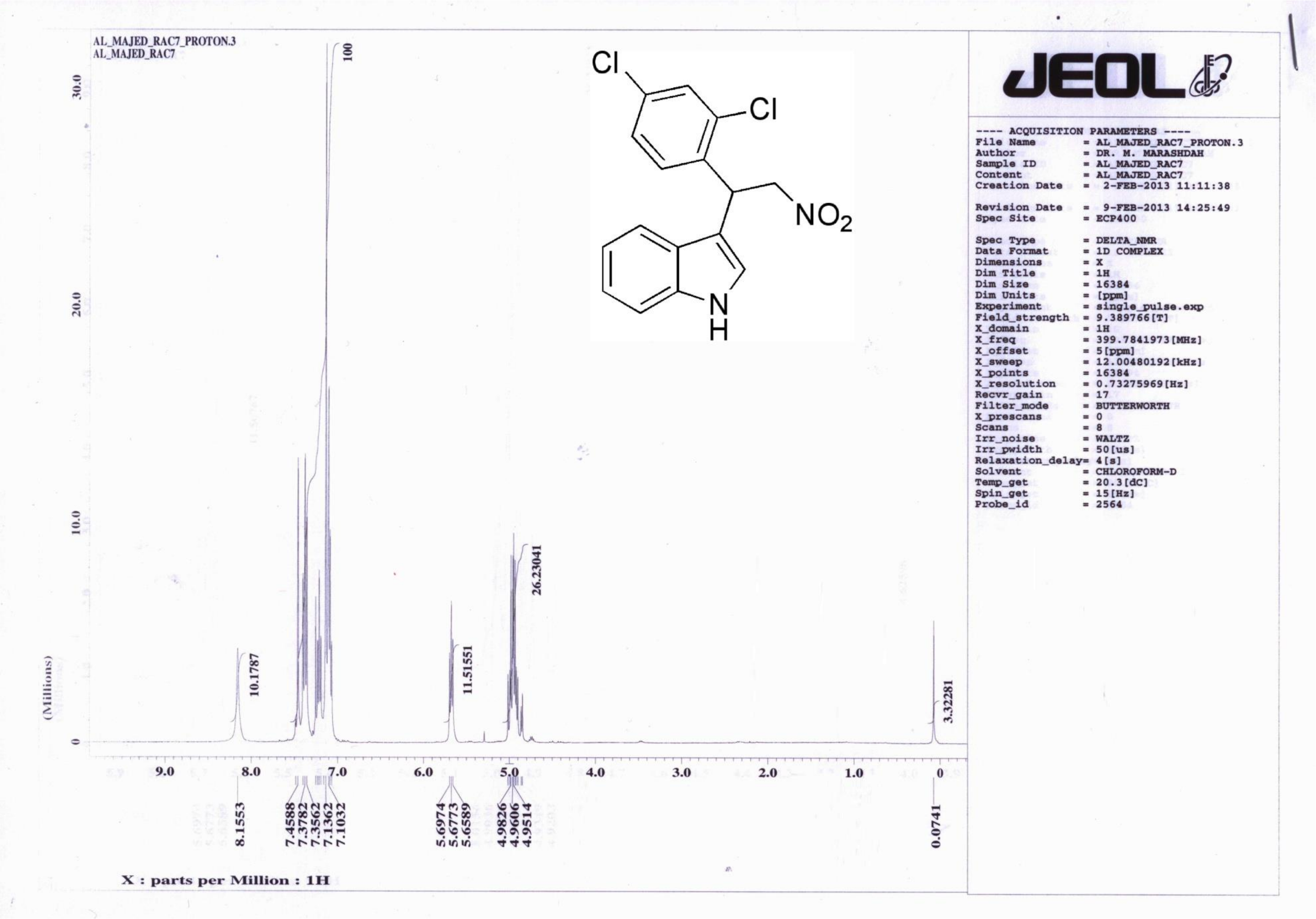

Compound 7g: ^{13}C-NMR

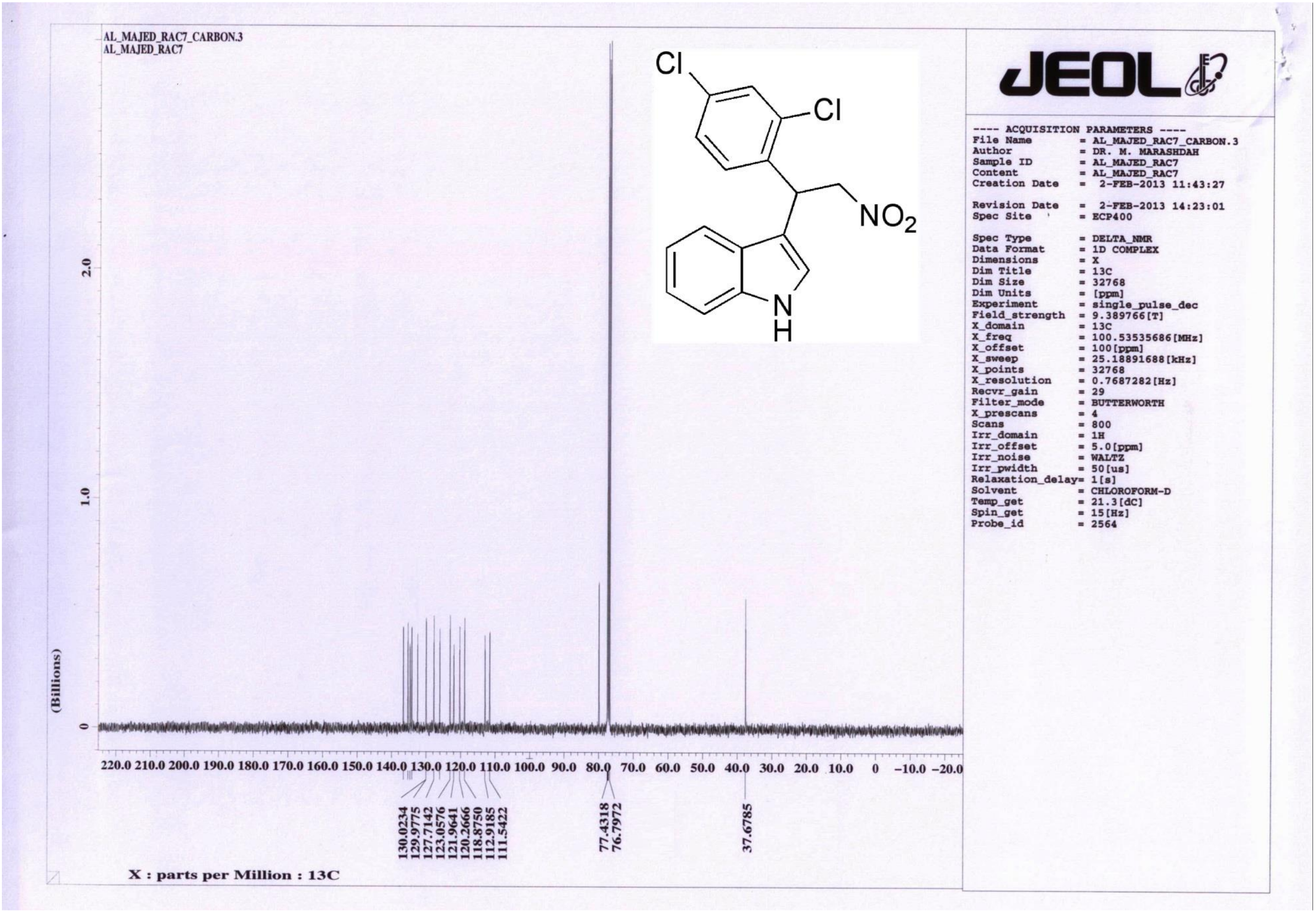

Compound 7h: ^{1}H-NMR

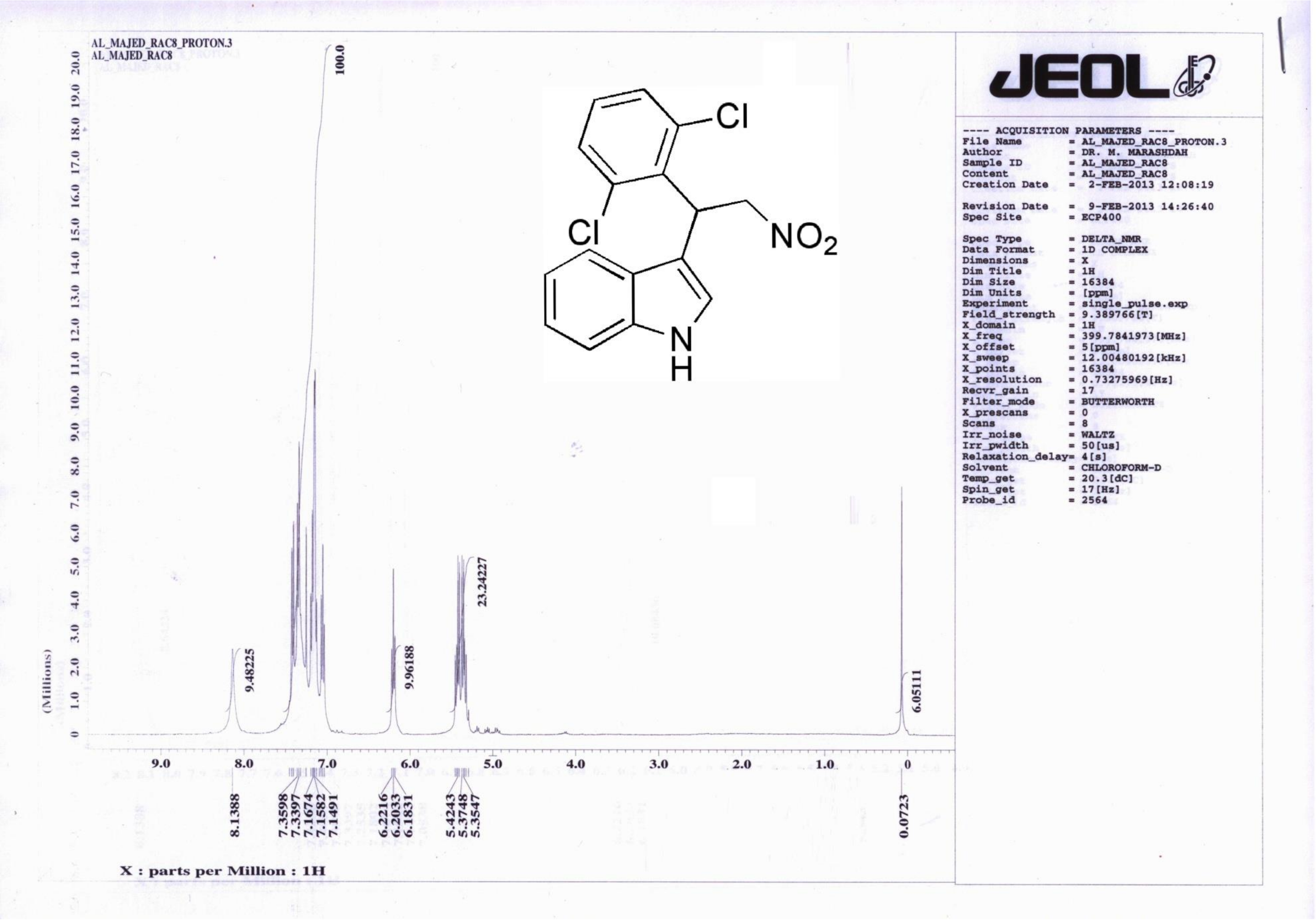

Compound 7h: ^{13}C-NMR

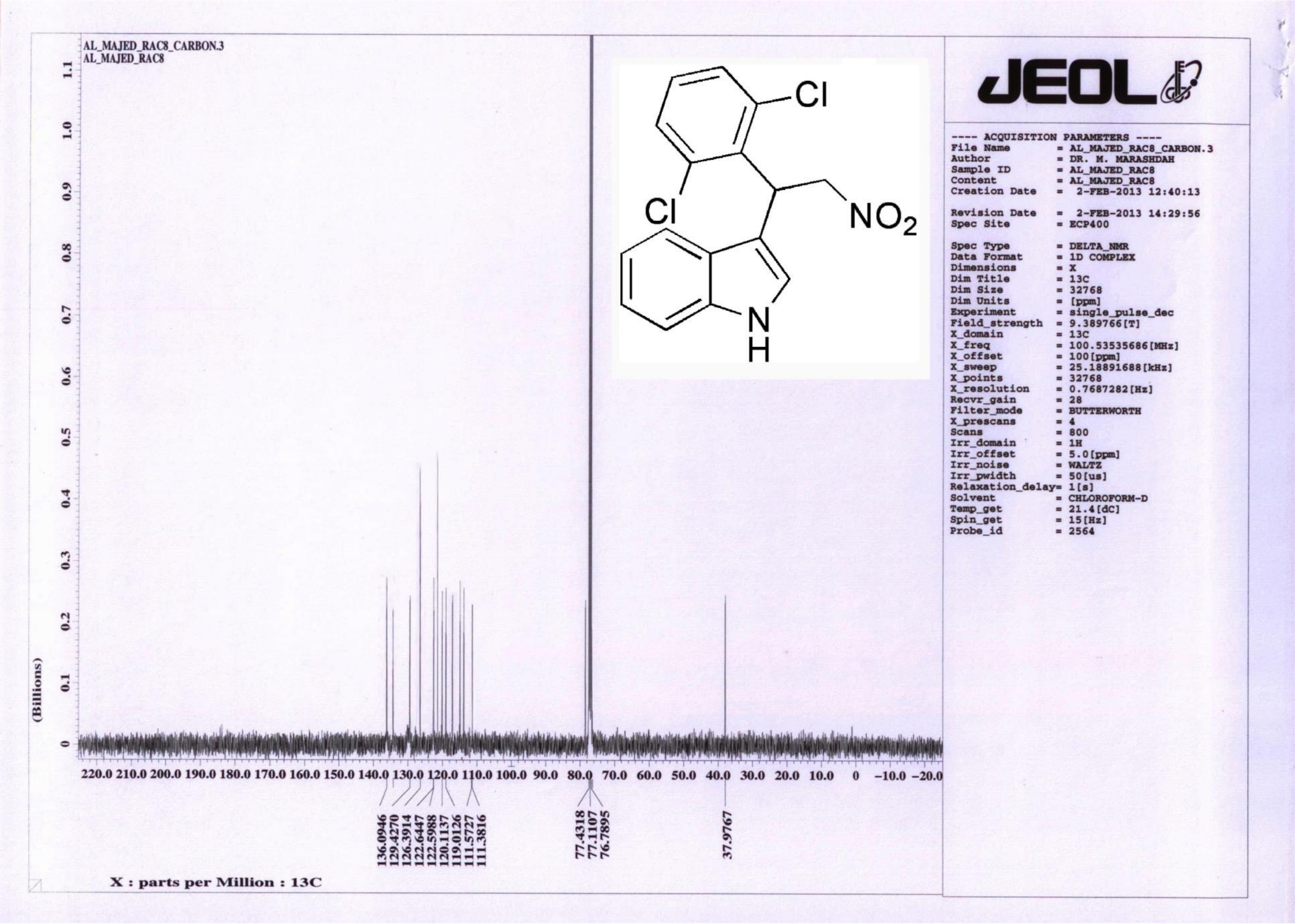

Compound 7i: ^{1}H-NMR

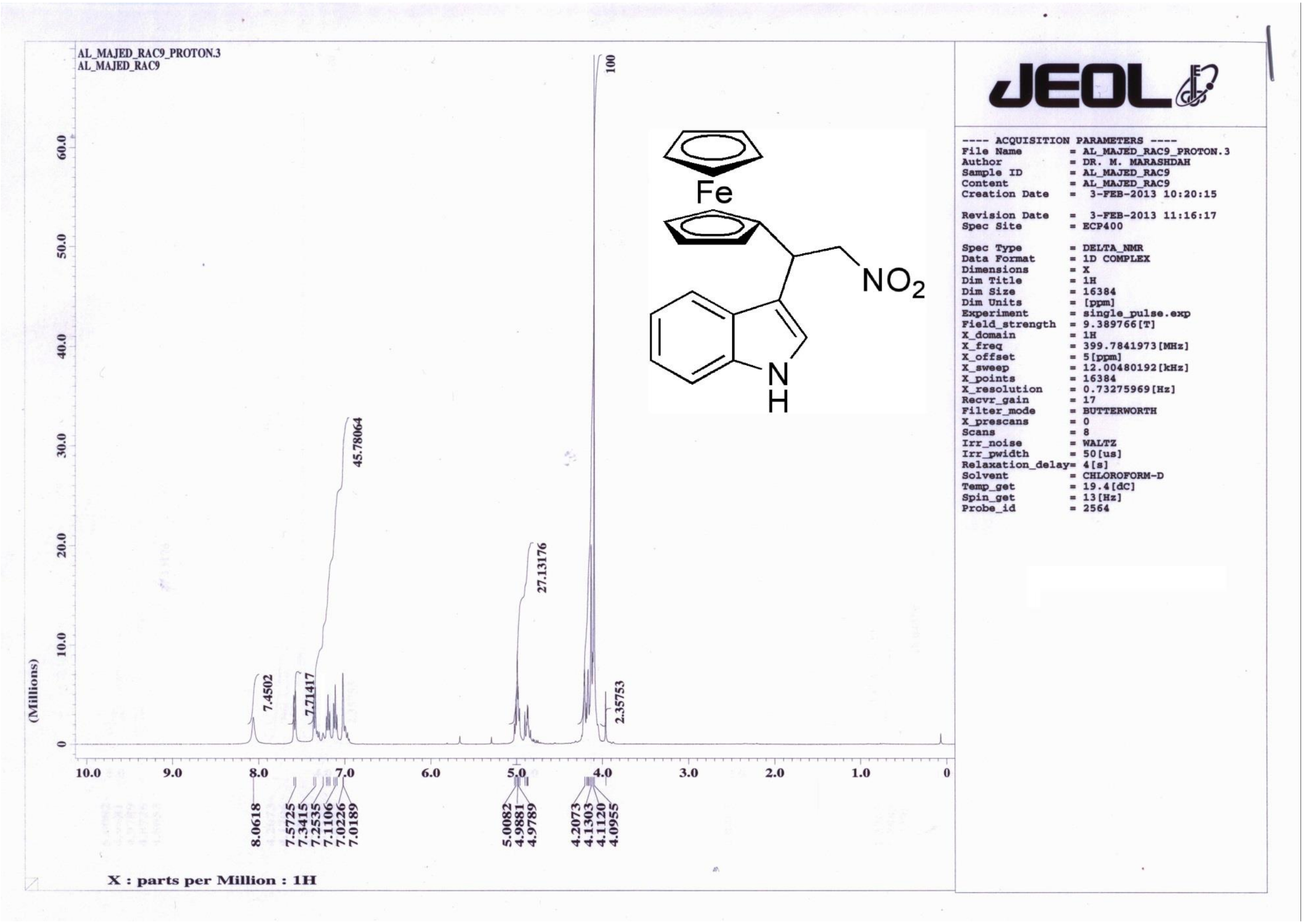

Compound 7i: ^{13}C-NMR

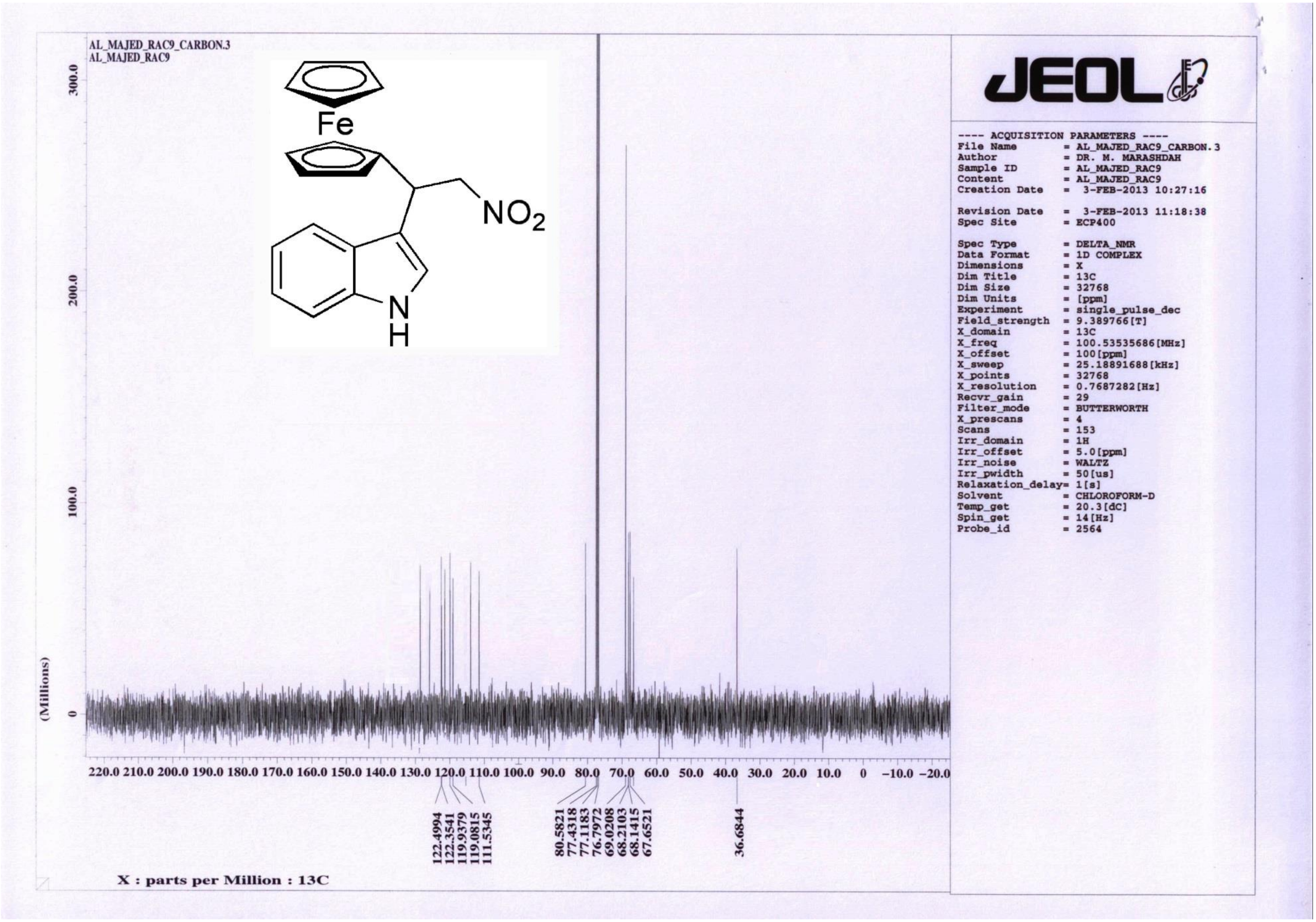

Compound 8a: ^{1}H-NMR

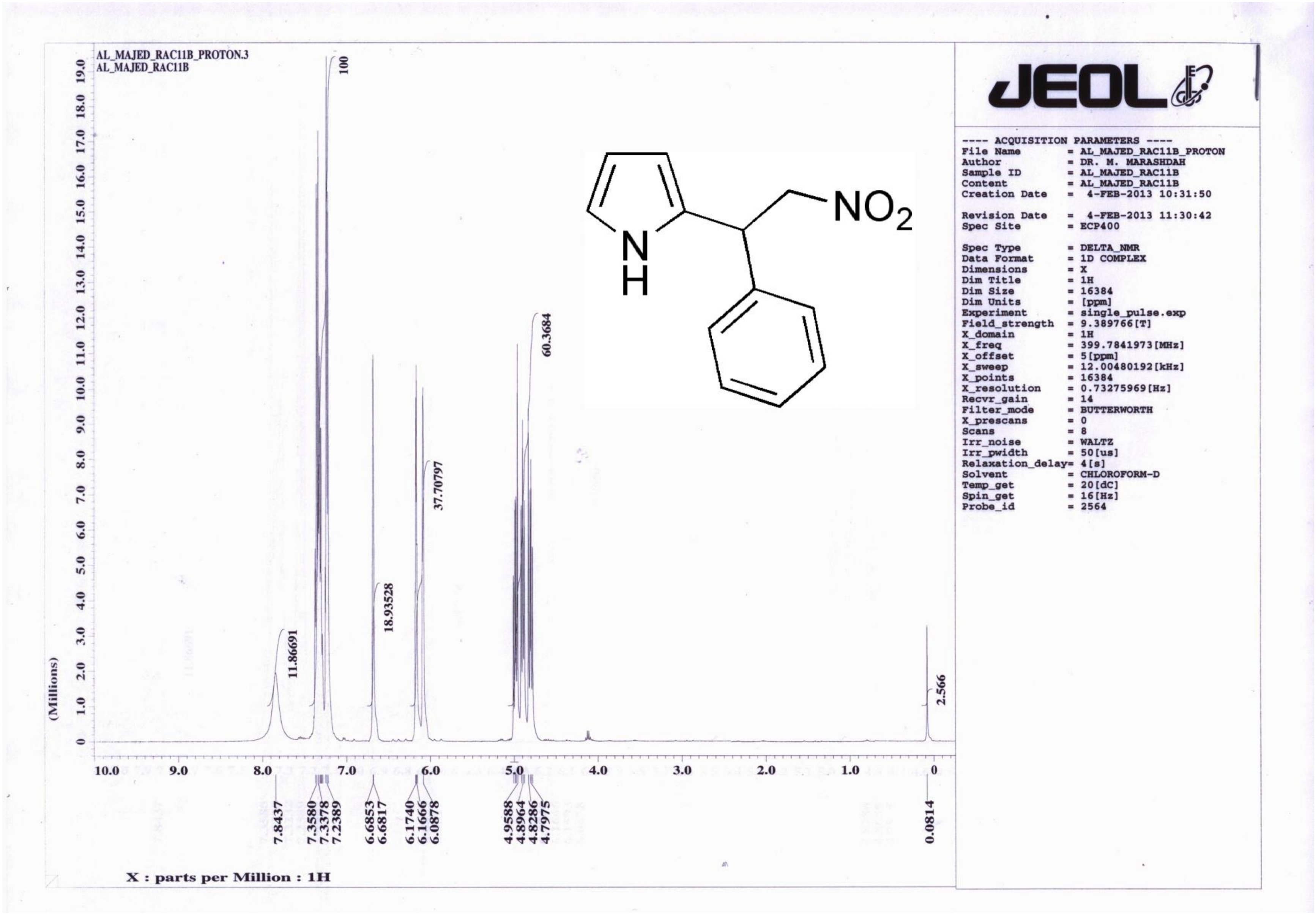

Compound 8a: ^{13}C-NMR

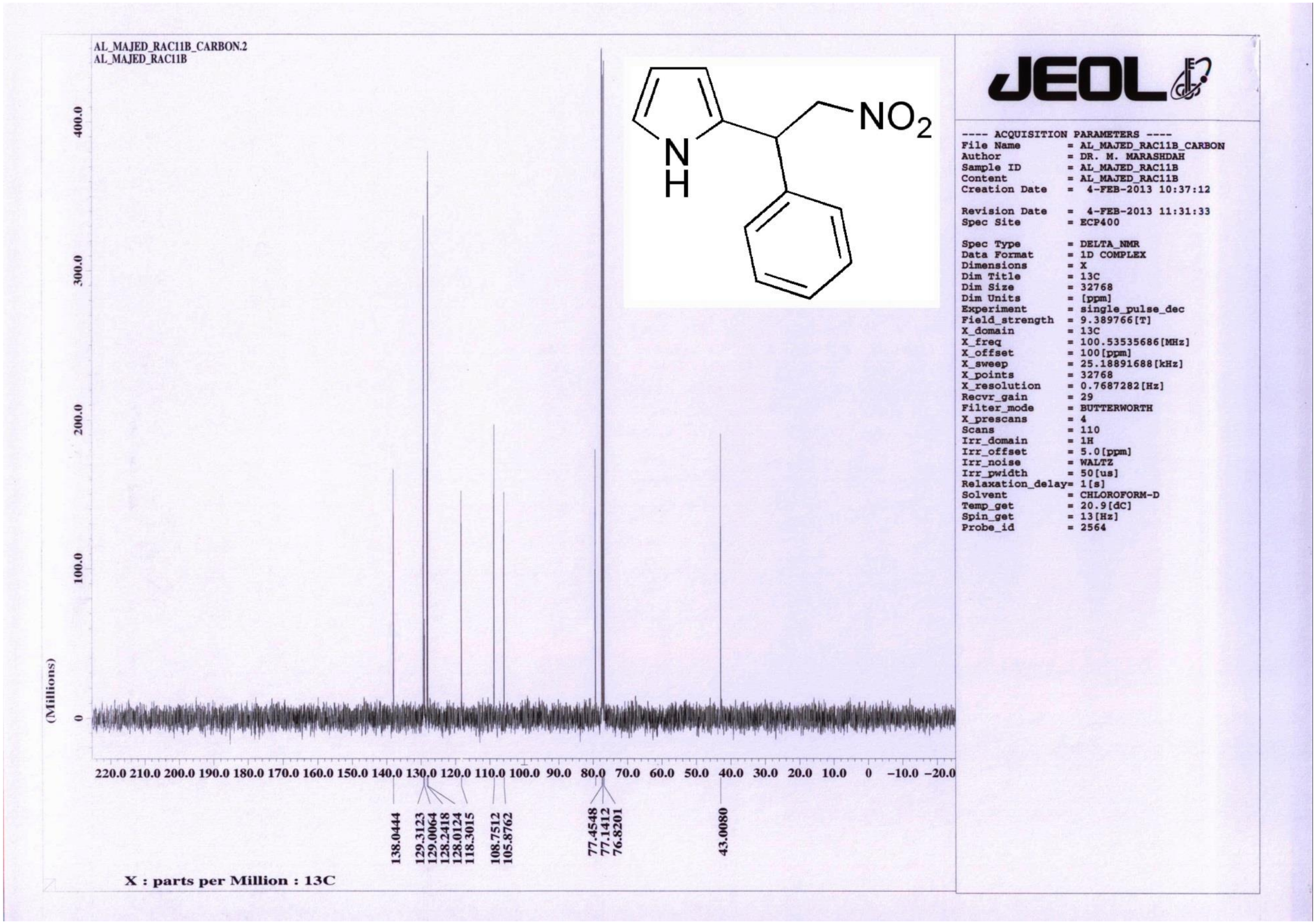

Compound 9a: ^{1}H-NMR

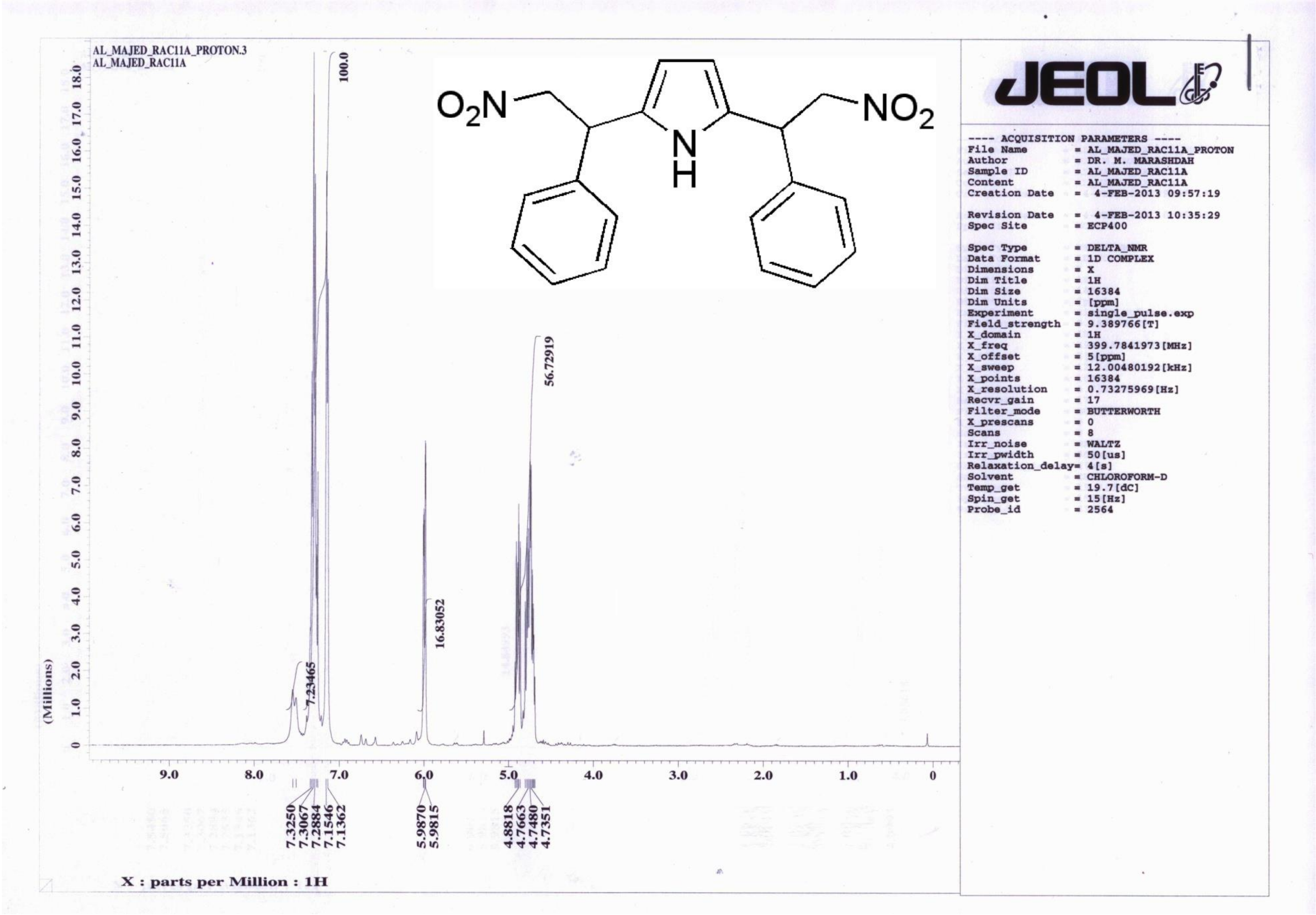

Compound 9a: ^{13}C-NMR

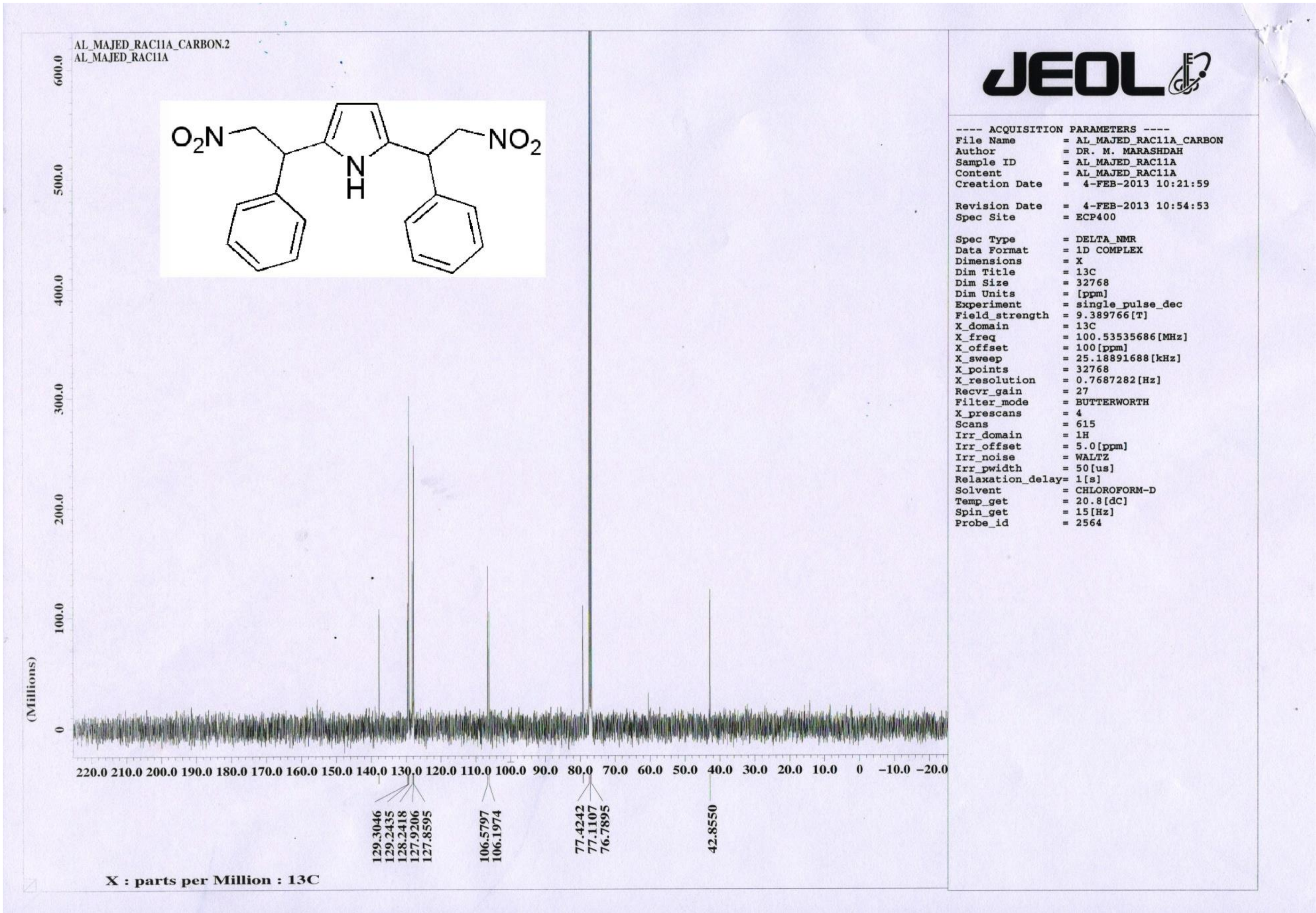

Compound 8b: ^{1}H-NMR

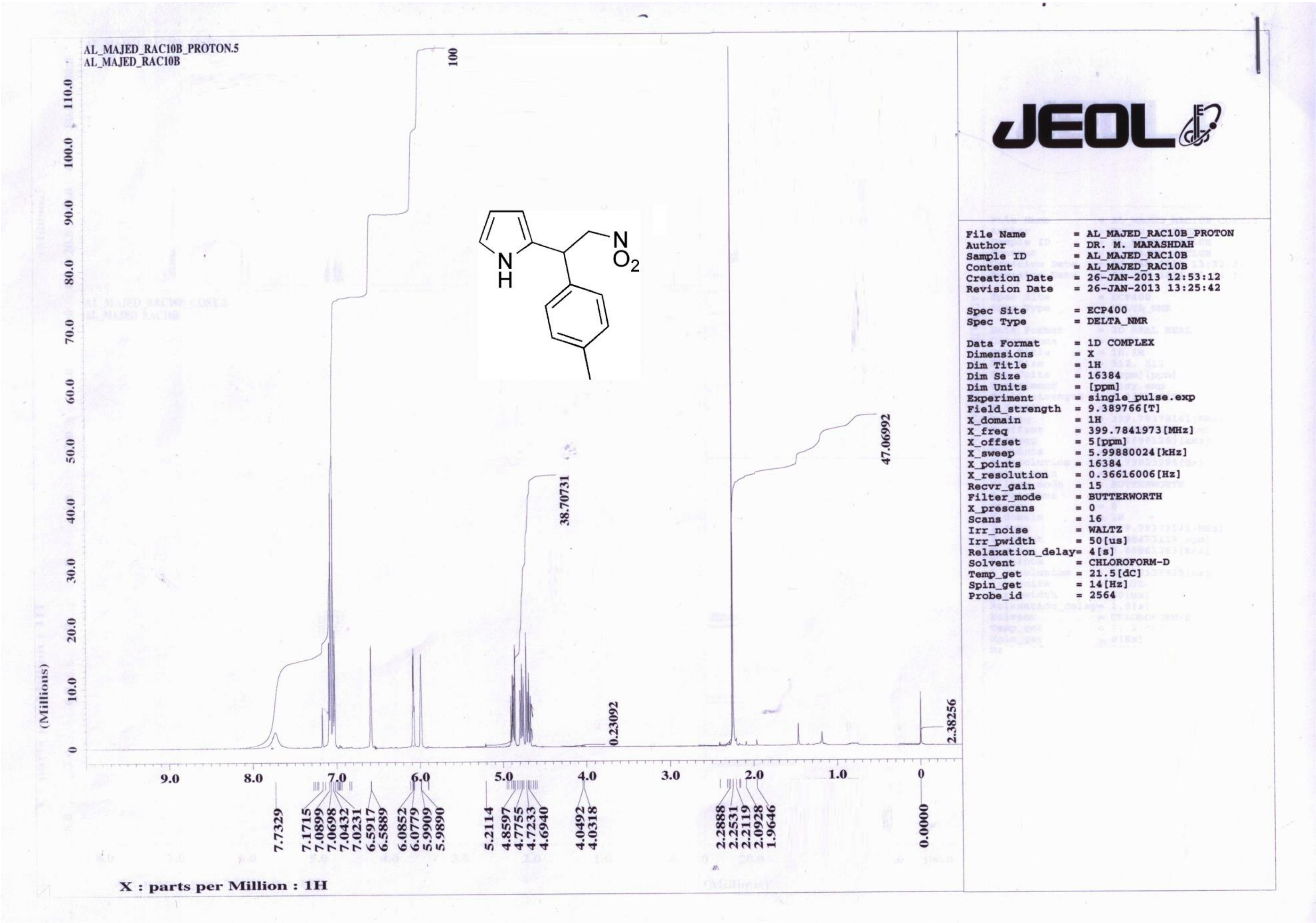

Compound 8b: ^{13}C-NMR

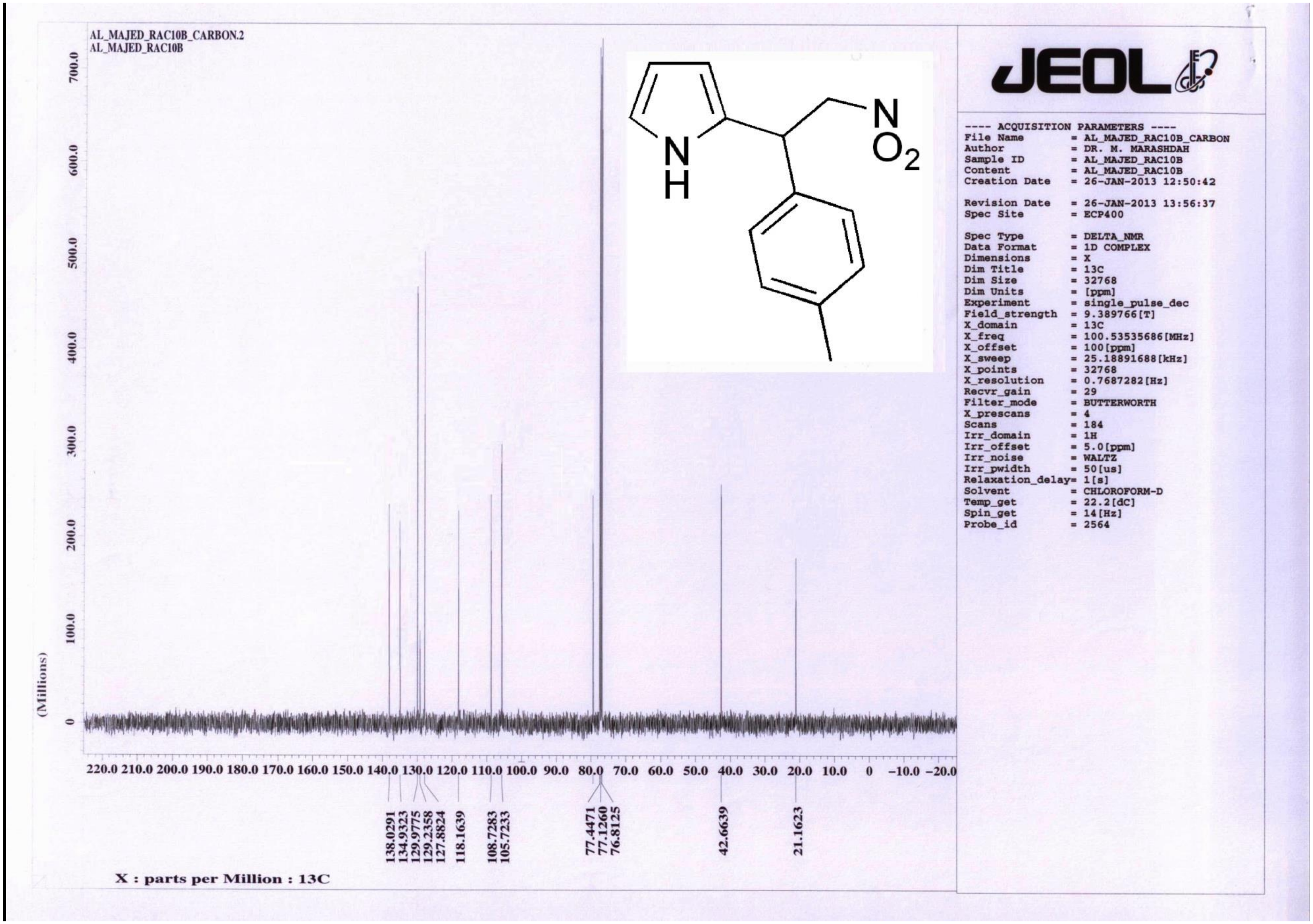

Compound 9b: ^{1}H-NMR

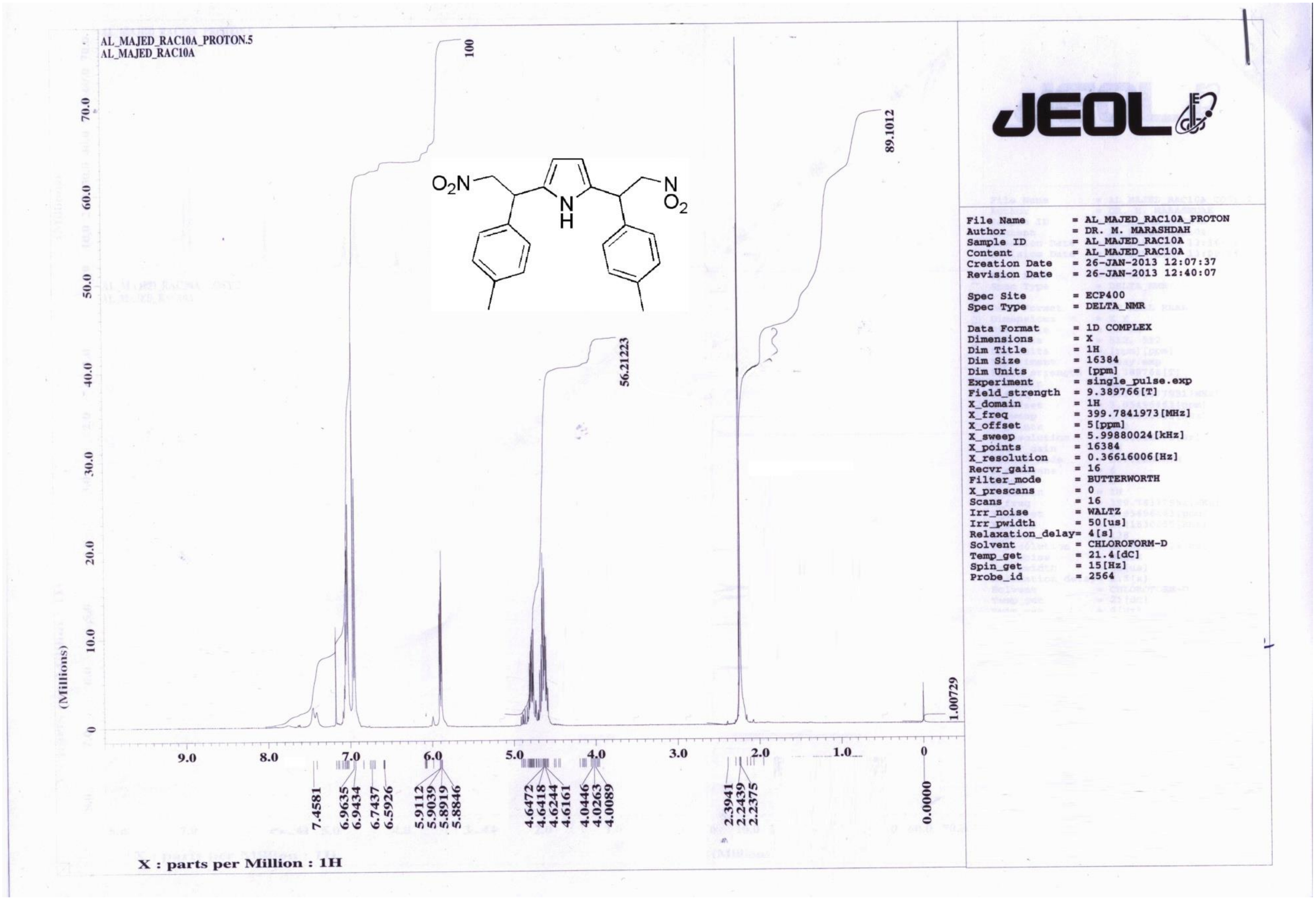

Compound 9b: ^{13}C-NMR

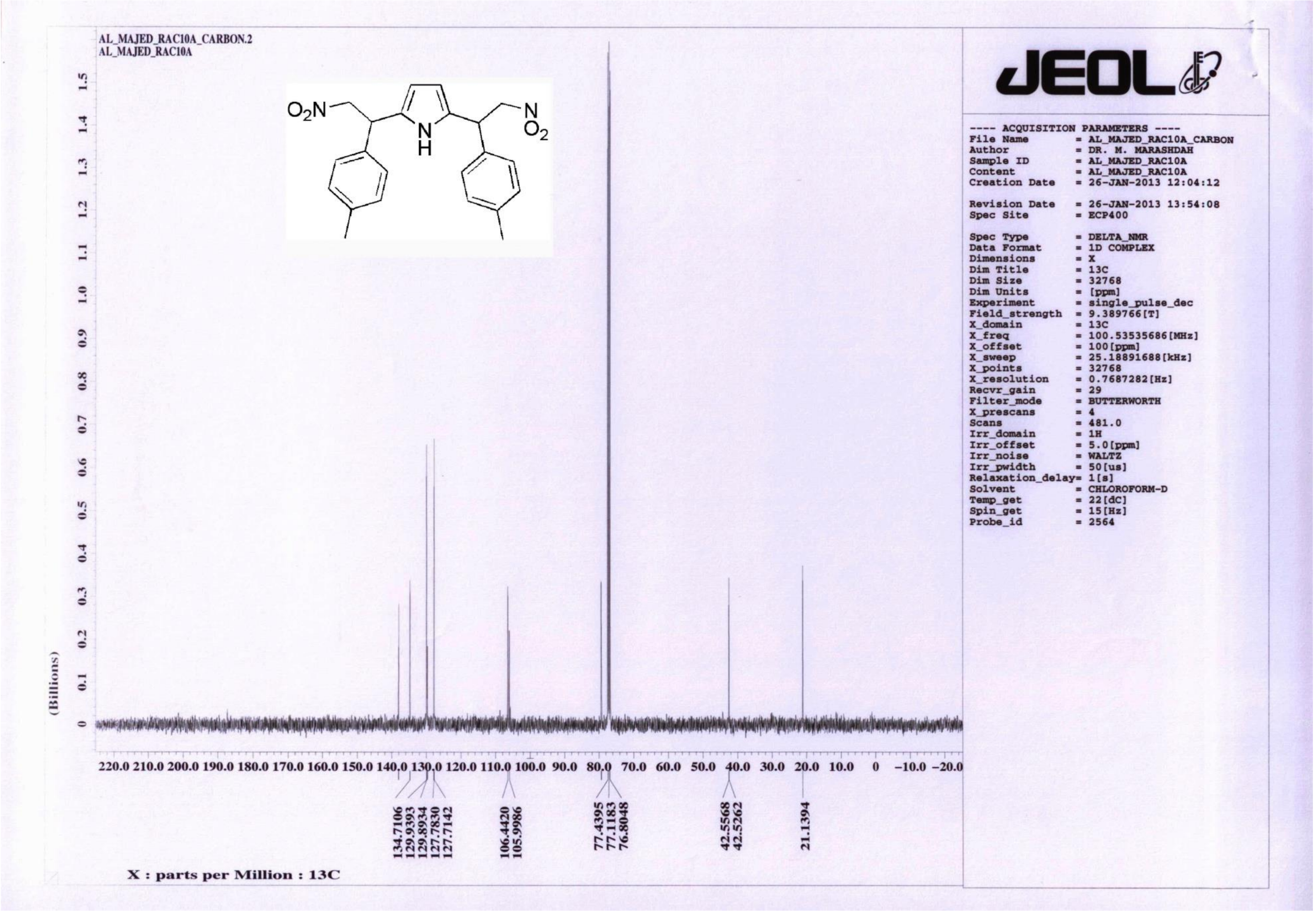

Compound 8c: ^{1}H-NMR

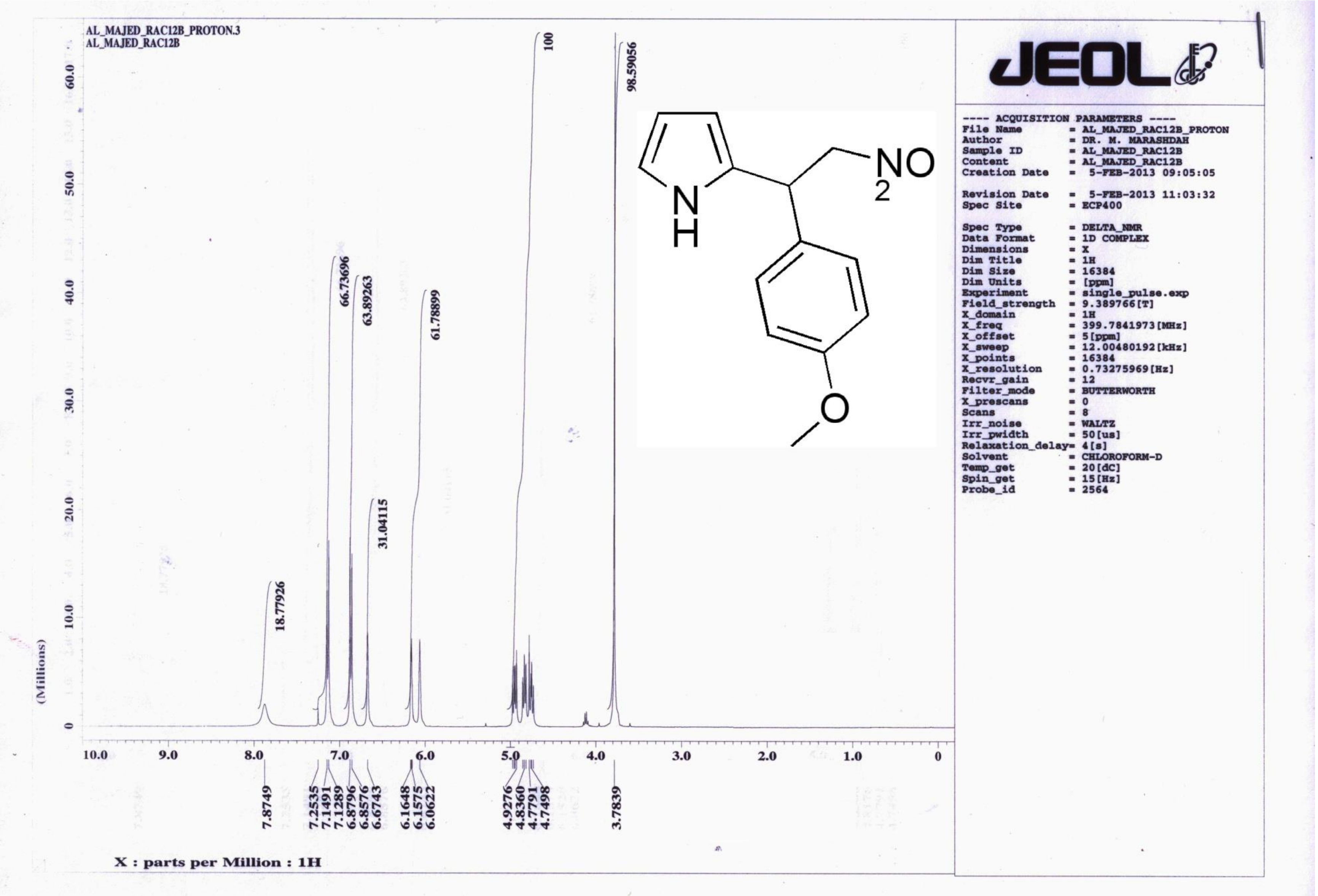

Compound 8c: ^{13}C-NMR

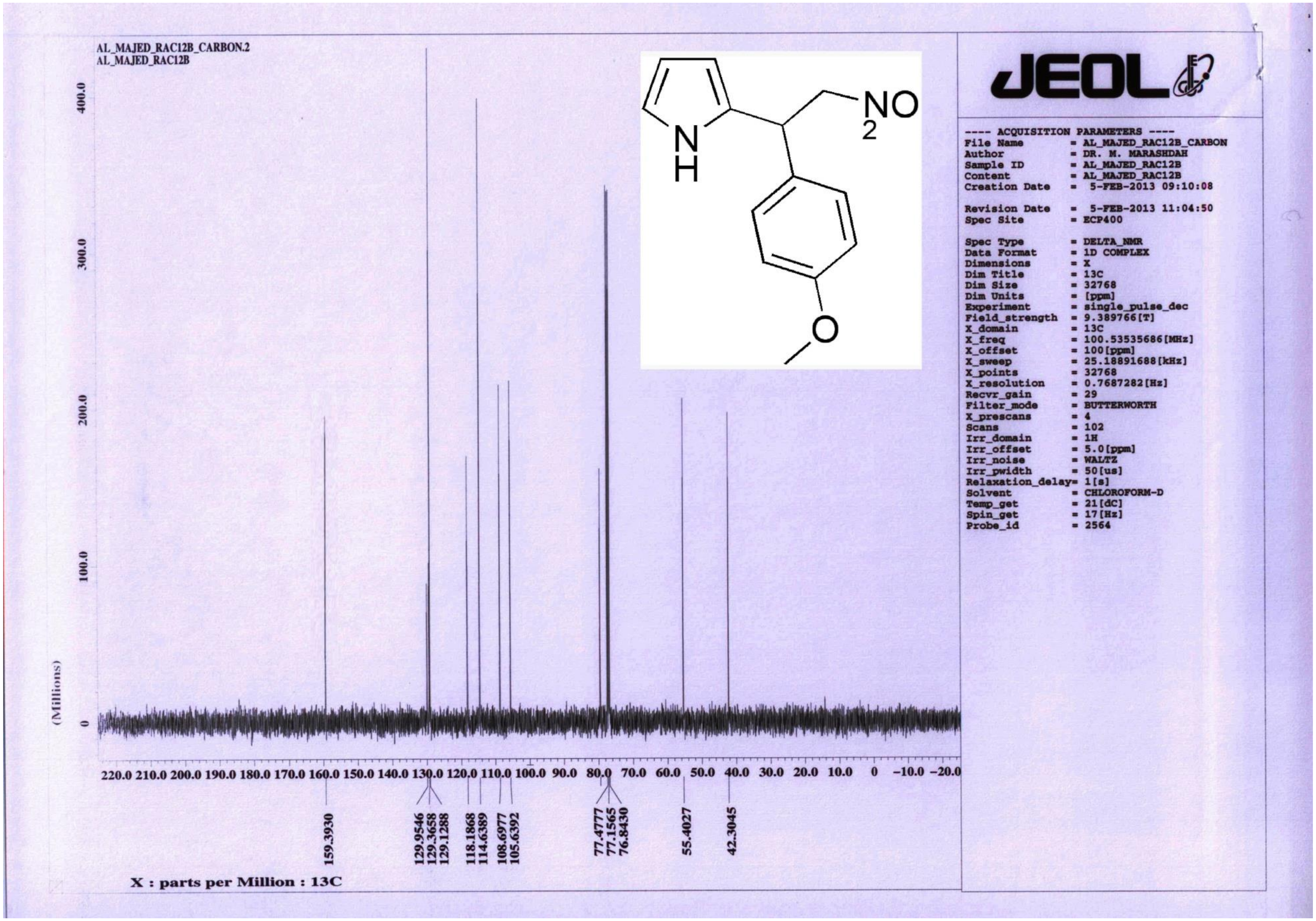

Compound 9c: ^{1}H-NMR

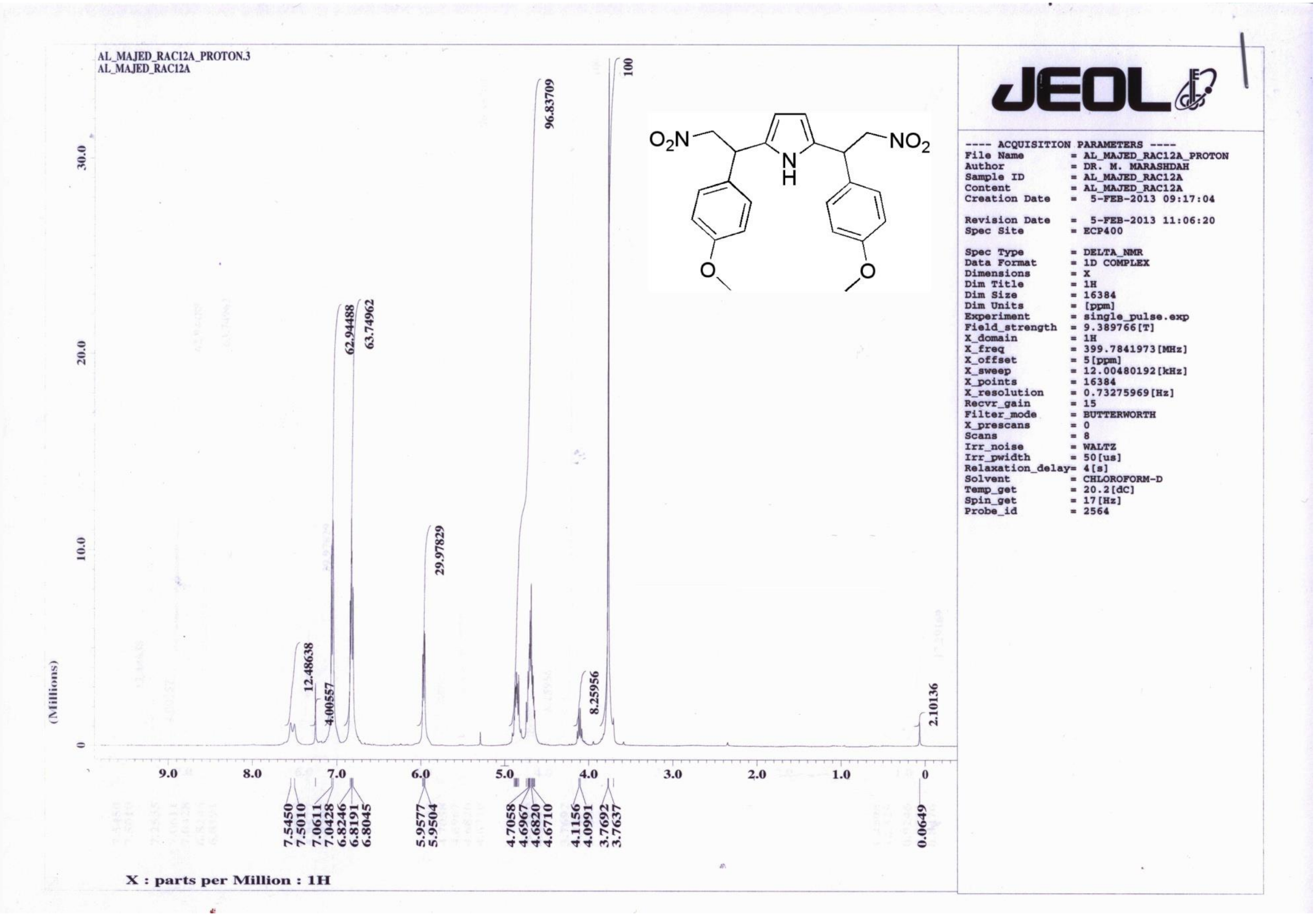

Compound 9c: ^{13}C-NMR

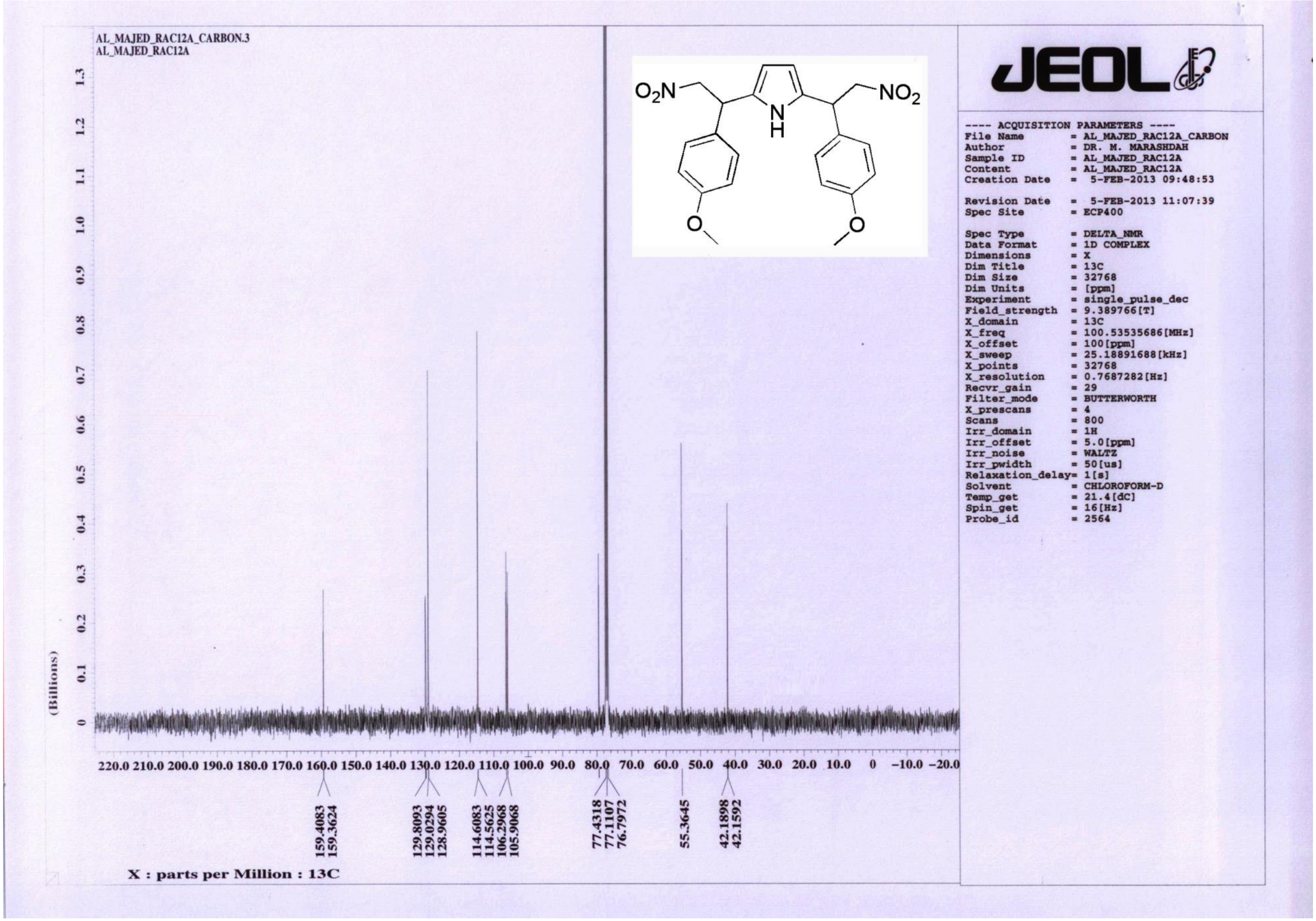

Compound 8d: ^{1}H-NMR

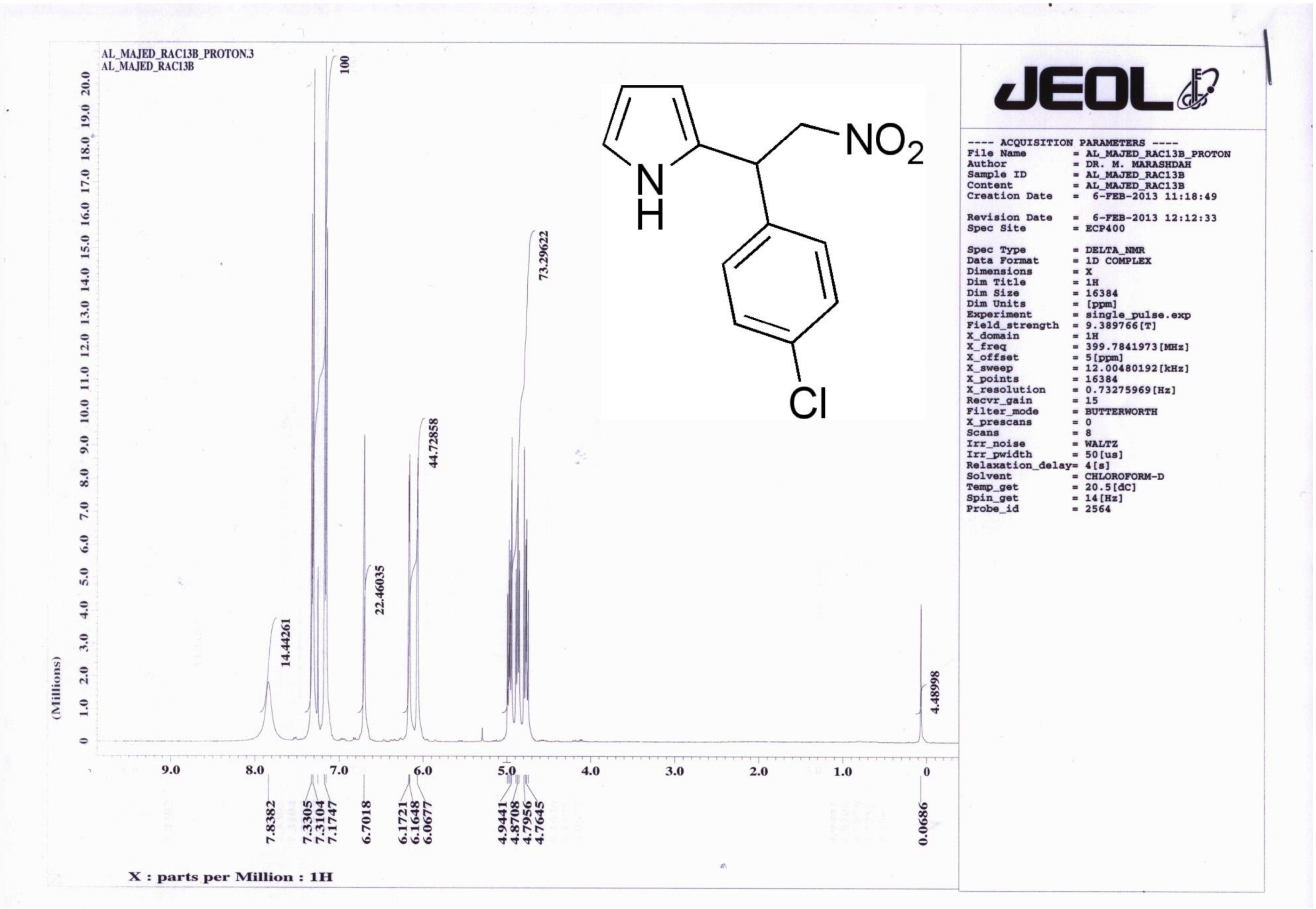

Compound 8d: ^{13}C-NMR

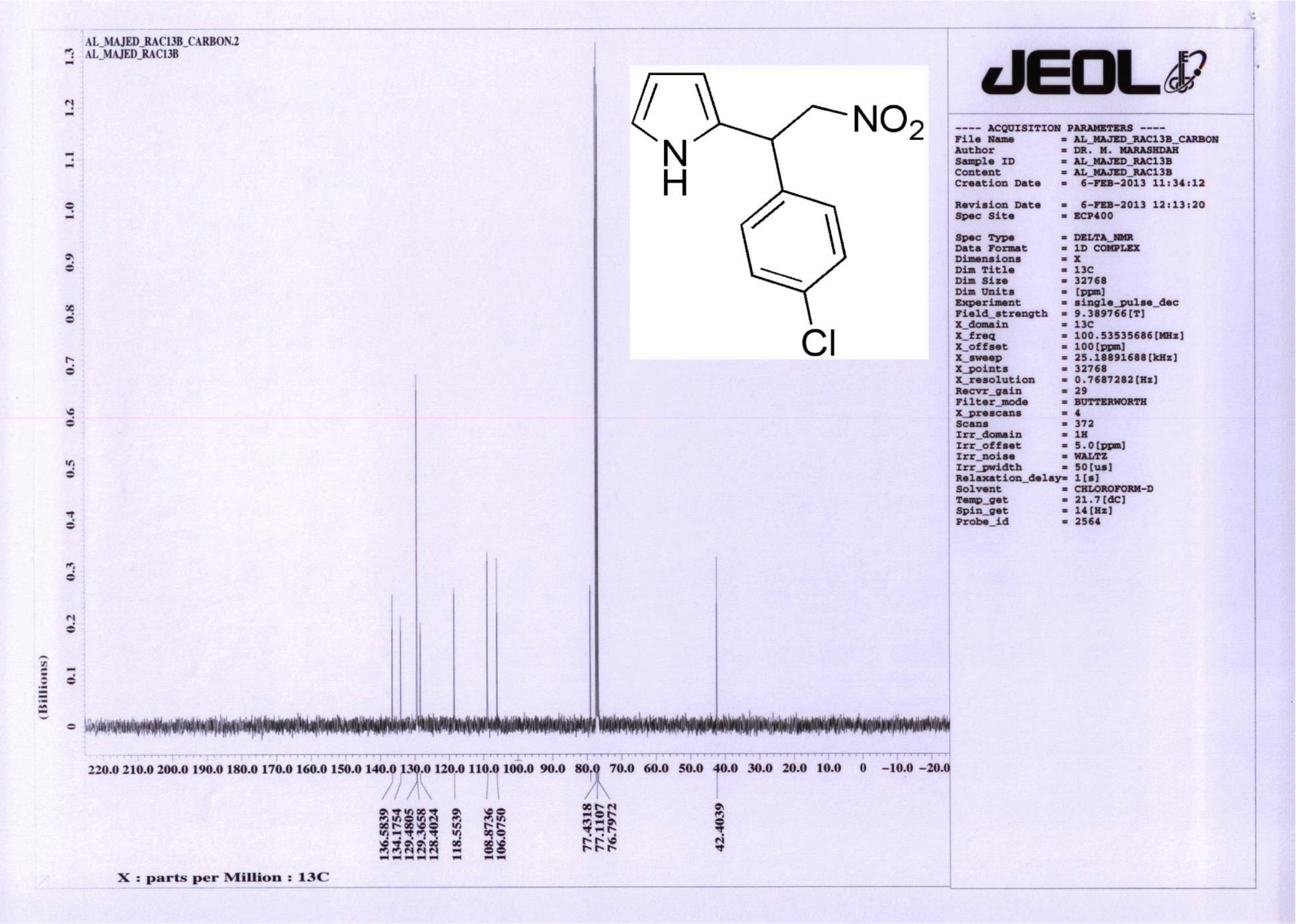

Compound 9d: ^{1}H-NMR

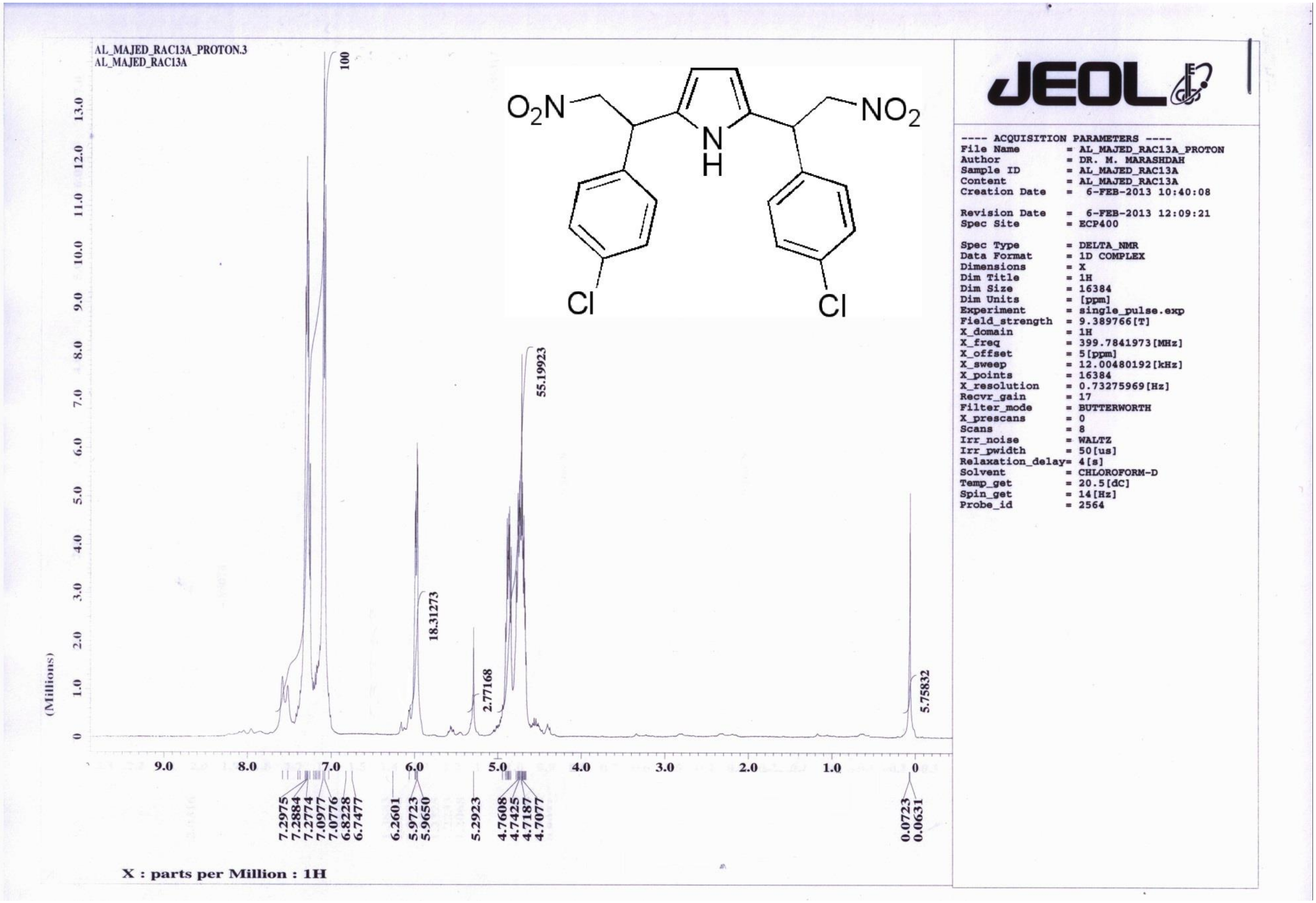

Compound 9d: ^{13}C-NMR

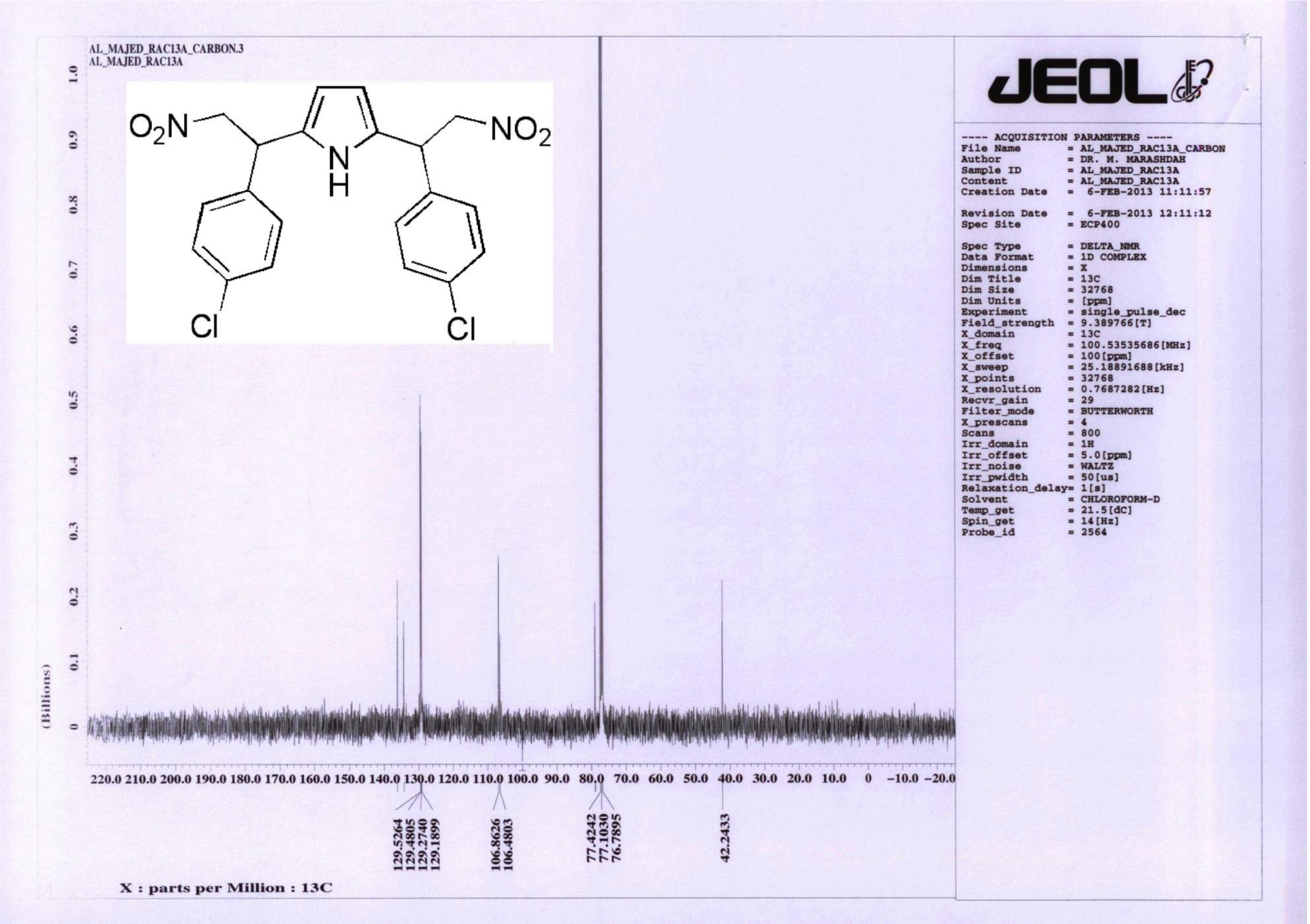

Compound 8e: ^{1}H-NMR

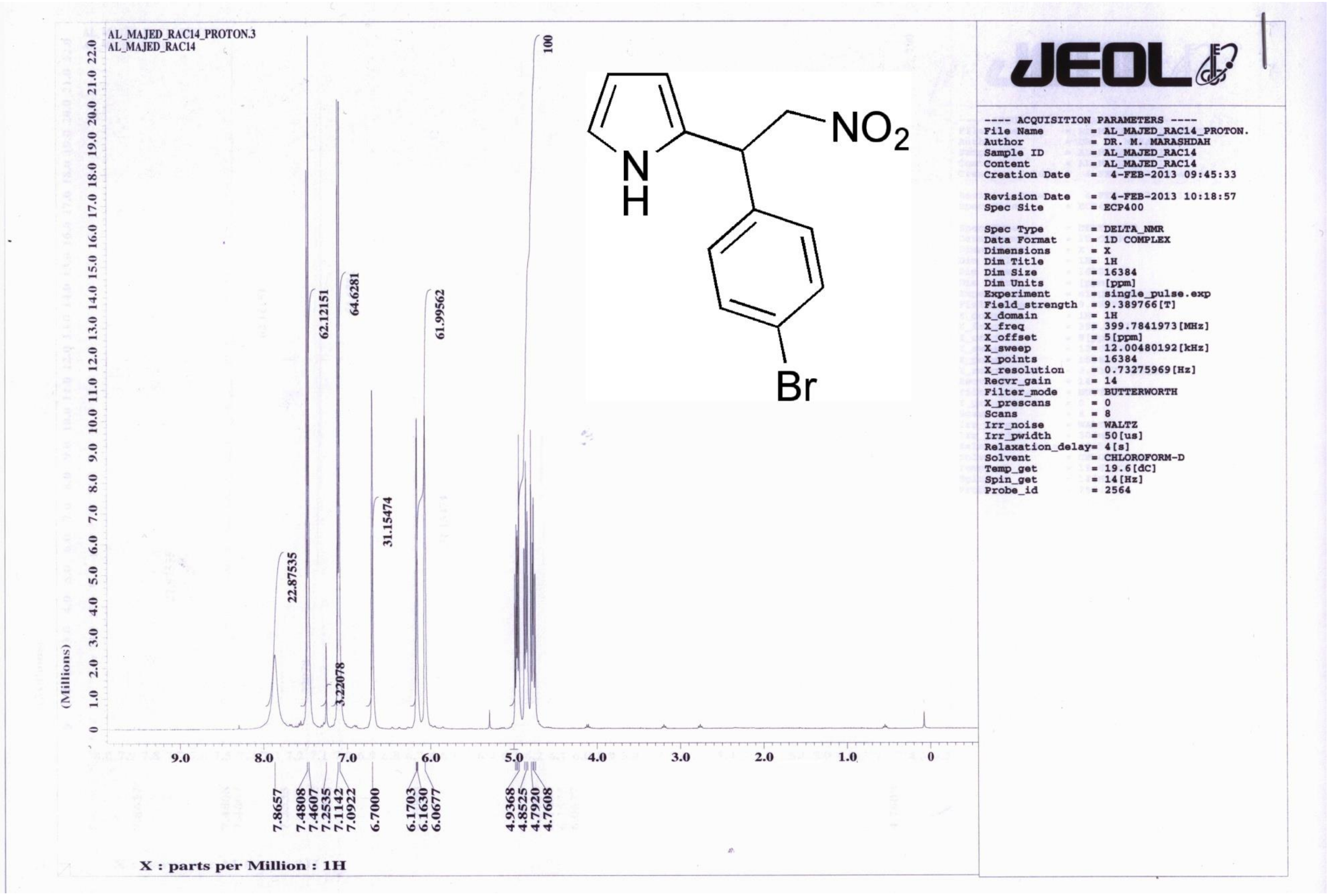

Compound 8e: ^{13}C-NMR

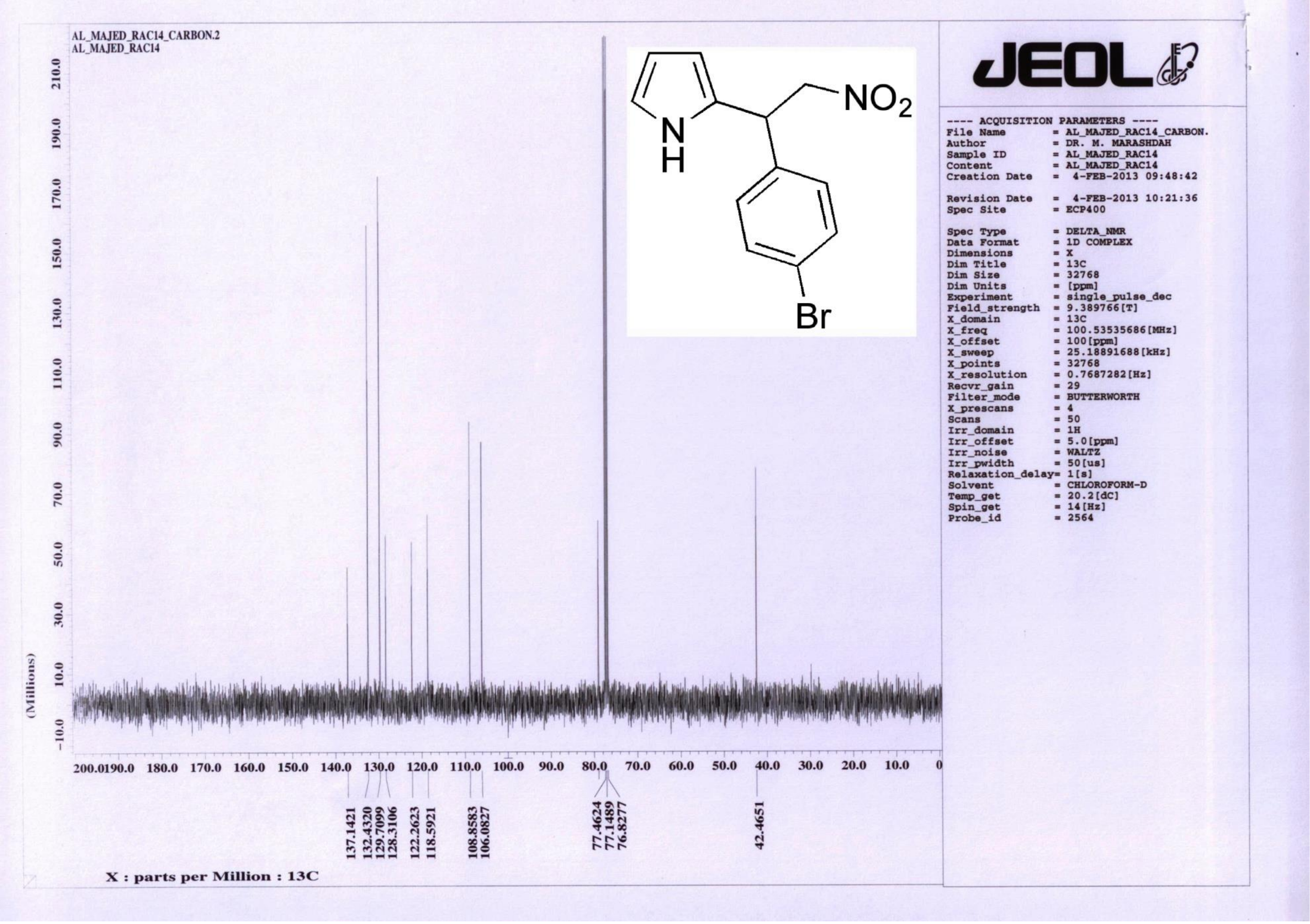

Compound 8f: ^{1}H-NMR

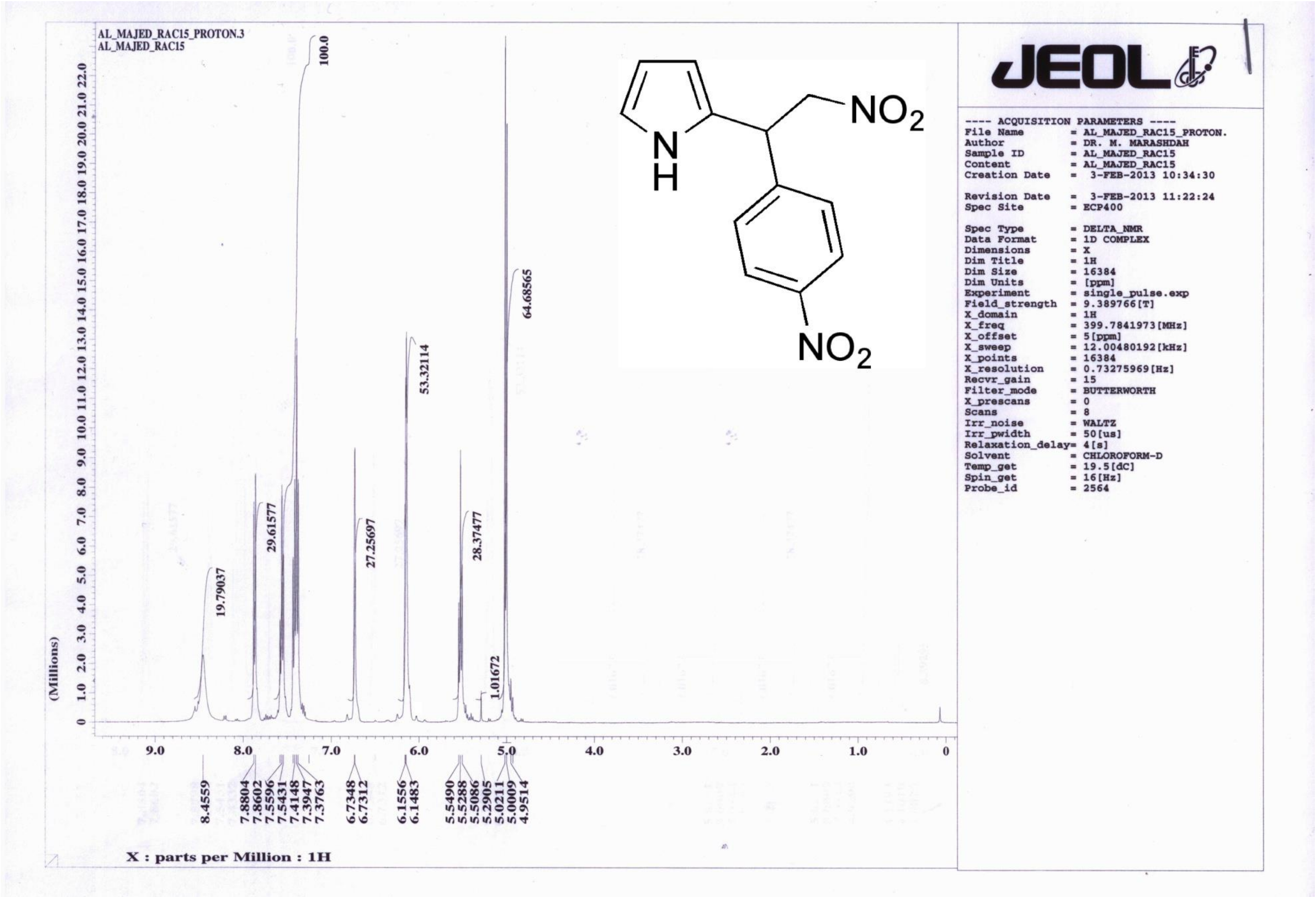

Compound 8f: ^{13}C-NMR

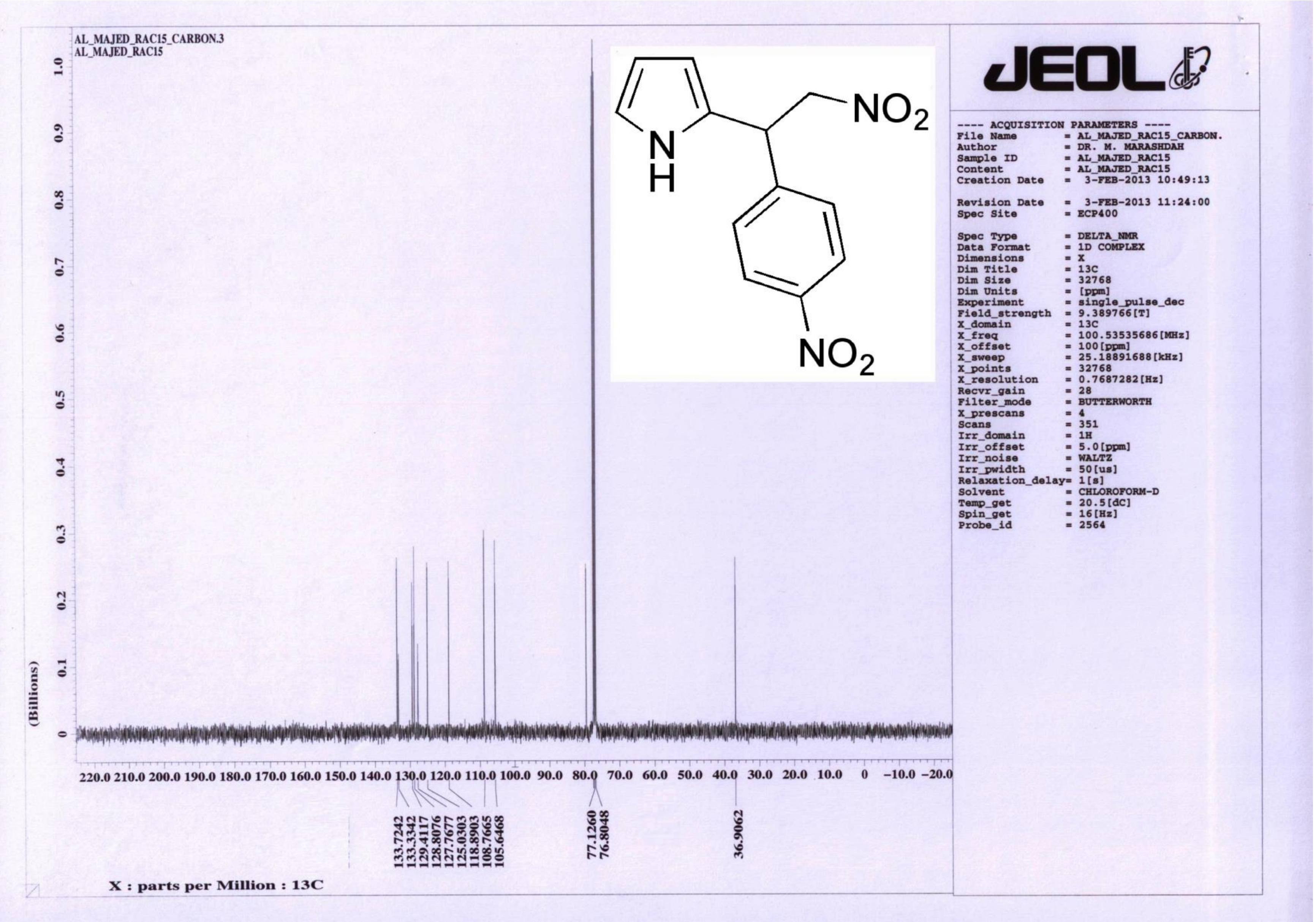

Compound 8g: ^{1}H-NMR

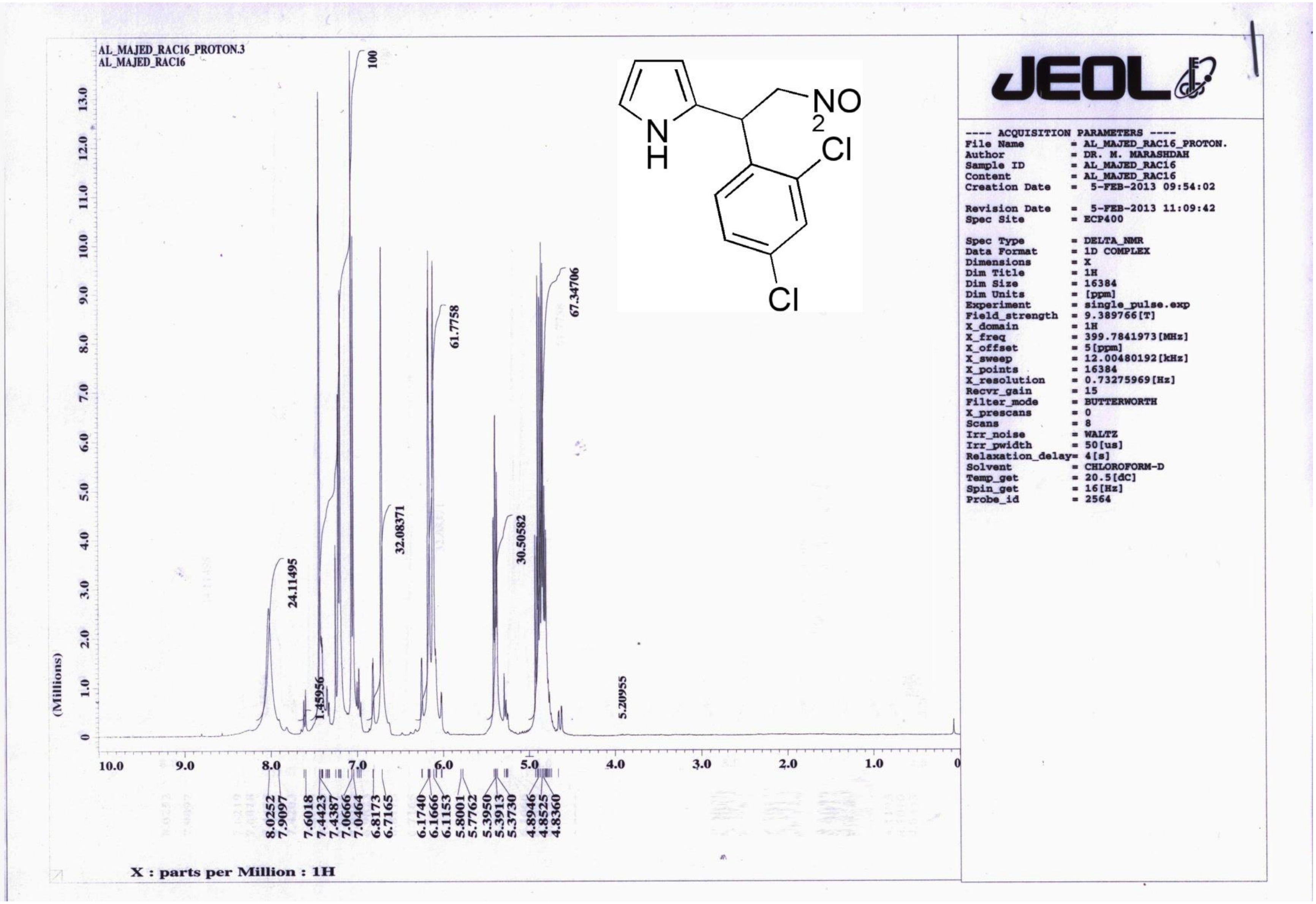

Compound 8g: ^{13}C-NMR

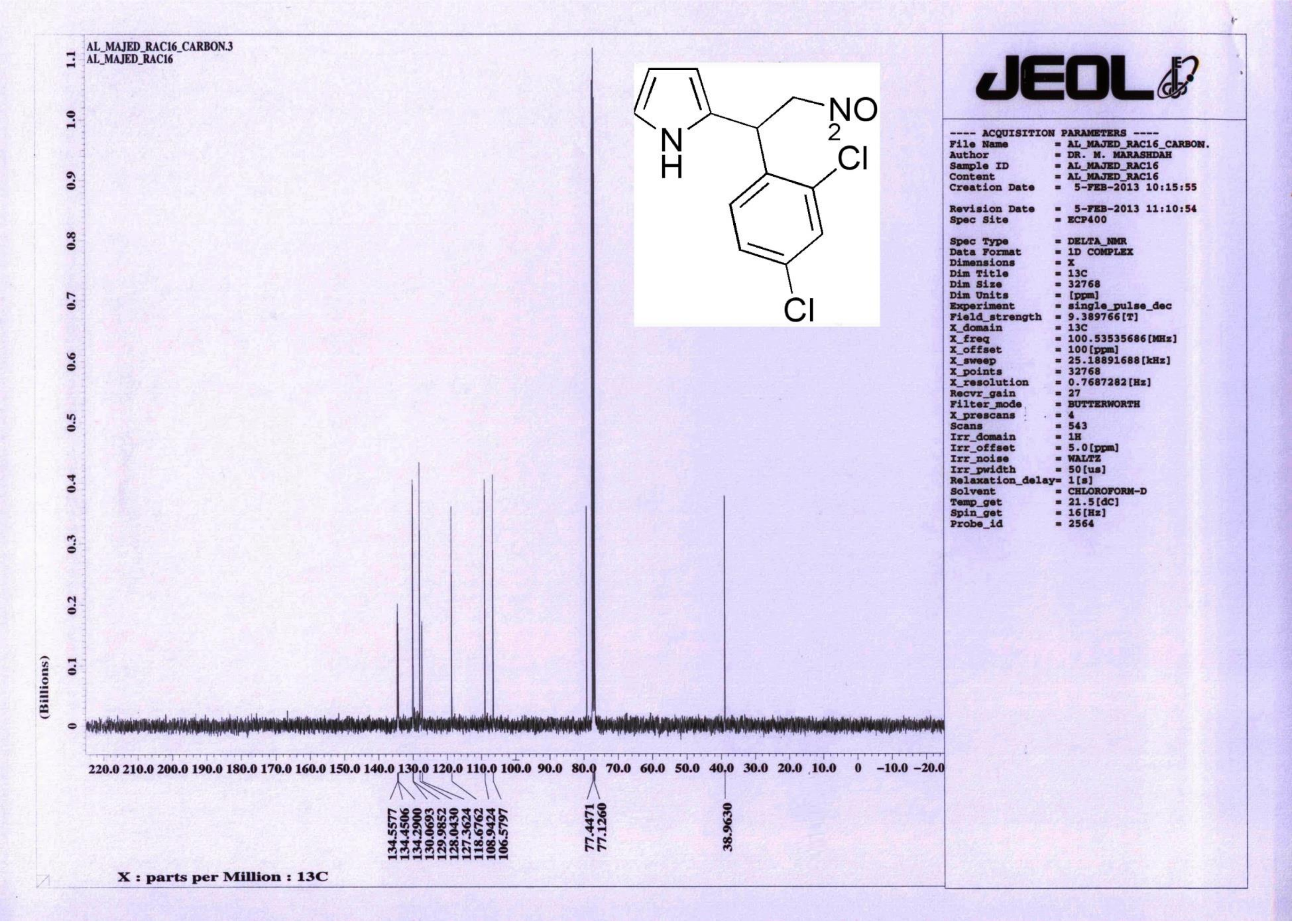

Compound 8h: ^{1}H-NMR

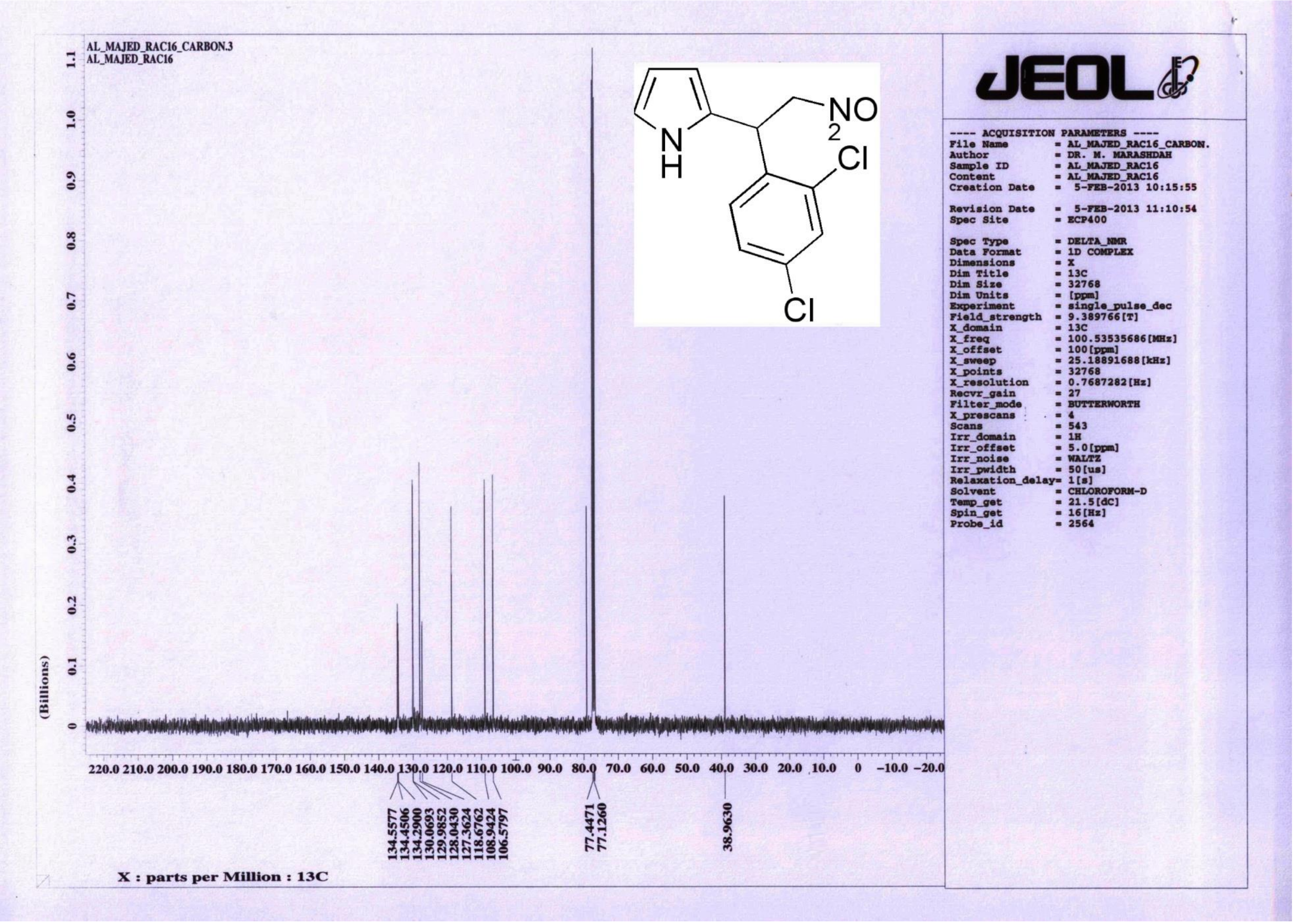

Compound 8h: ^{13}C-NMR

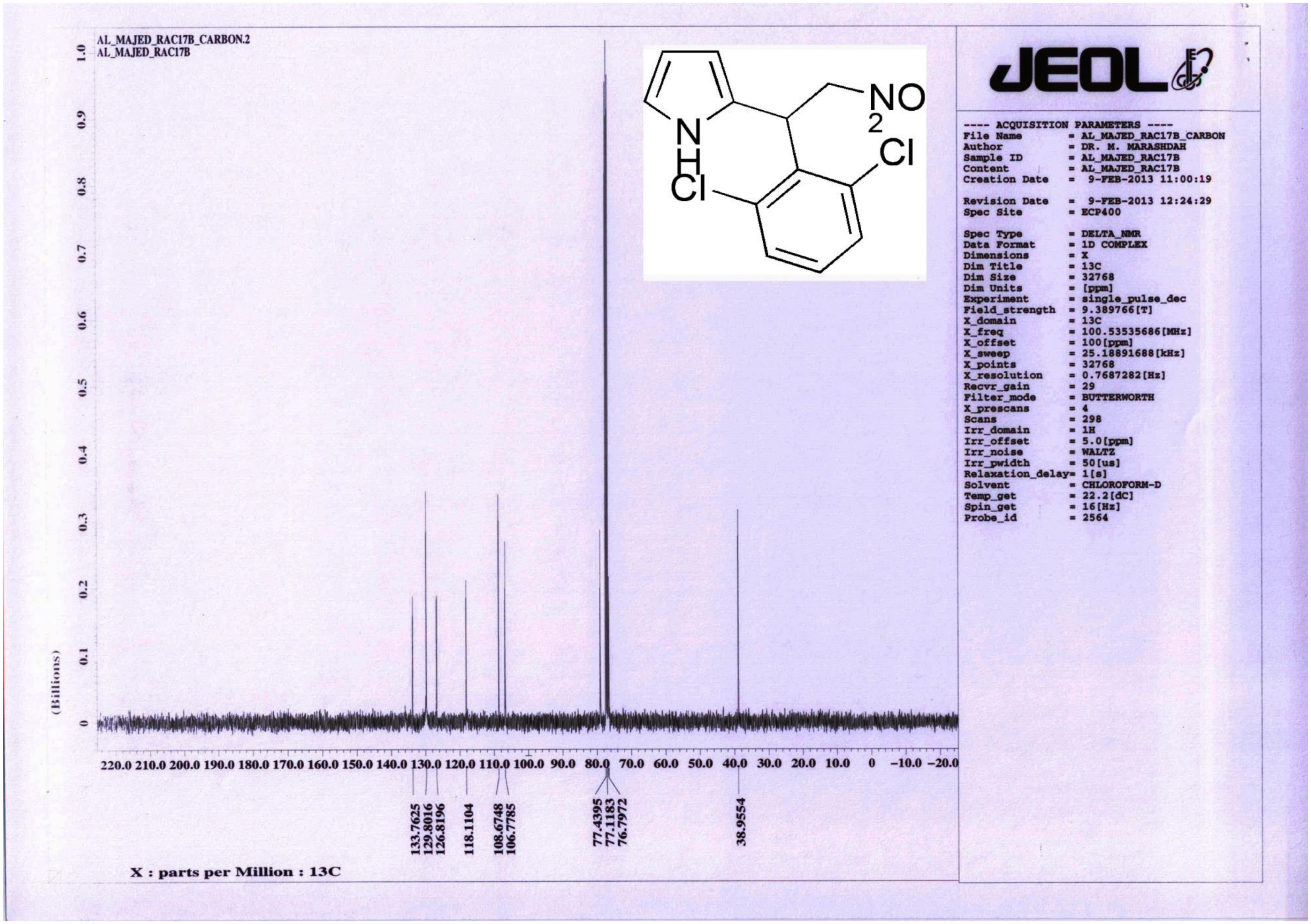

Compound 9h: ^{1}H-NMR

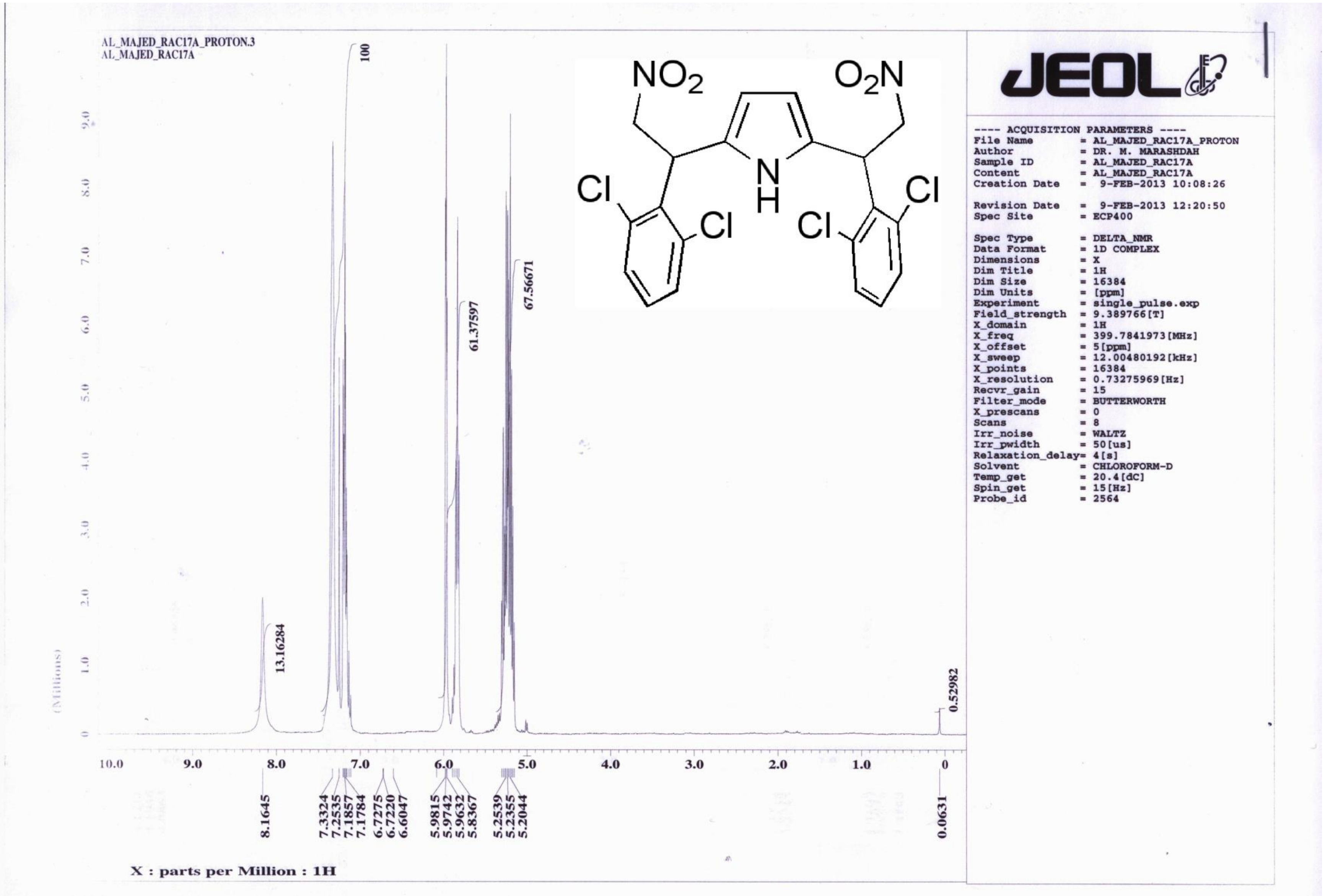

Compound 9h: ^{13}C-NMR

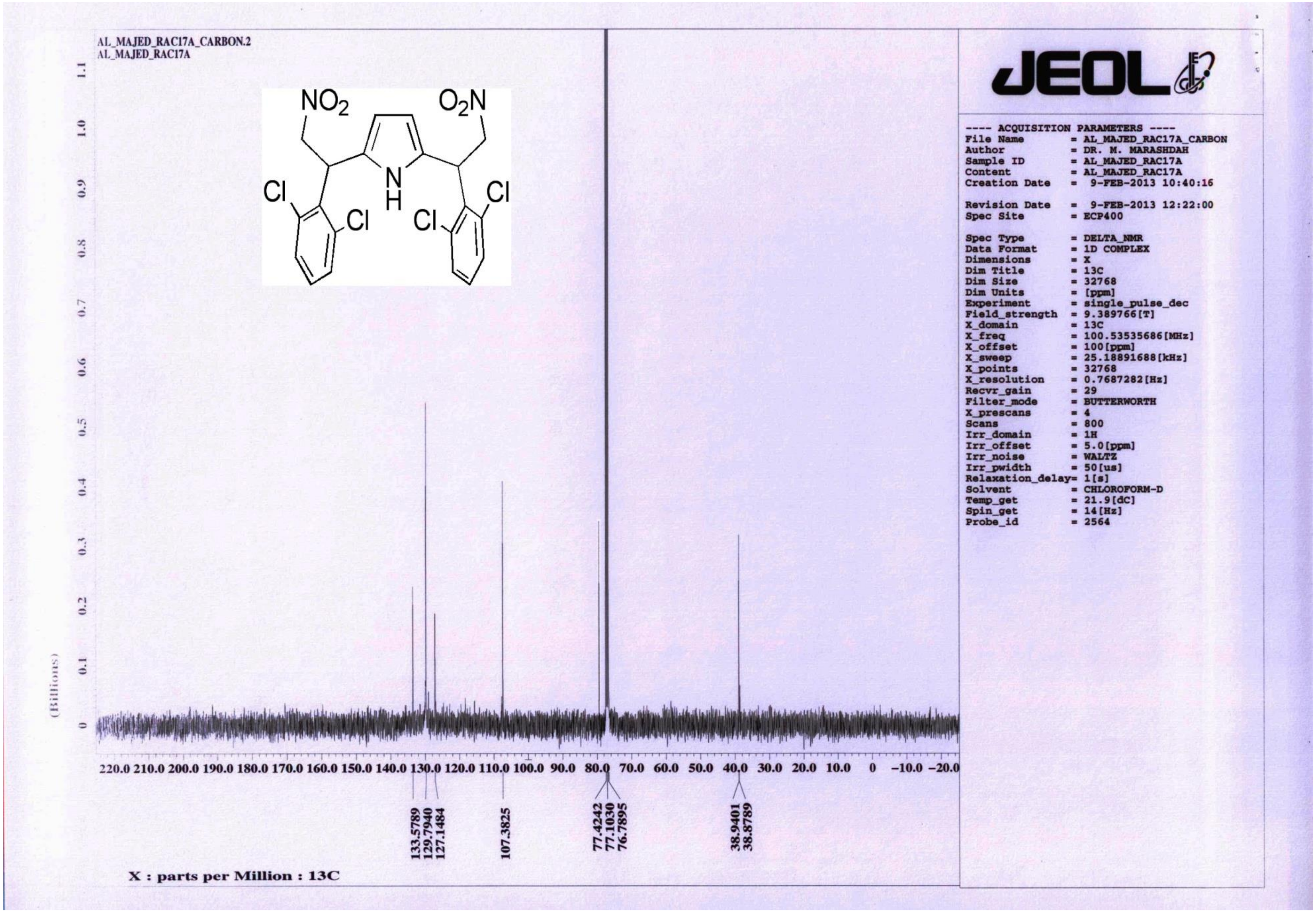

Compound 8i: ^{1}H-NMR

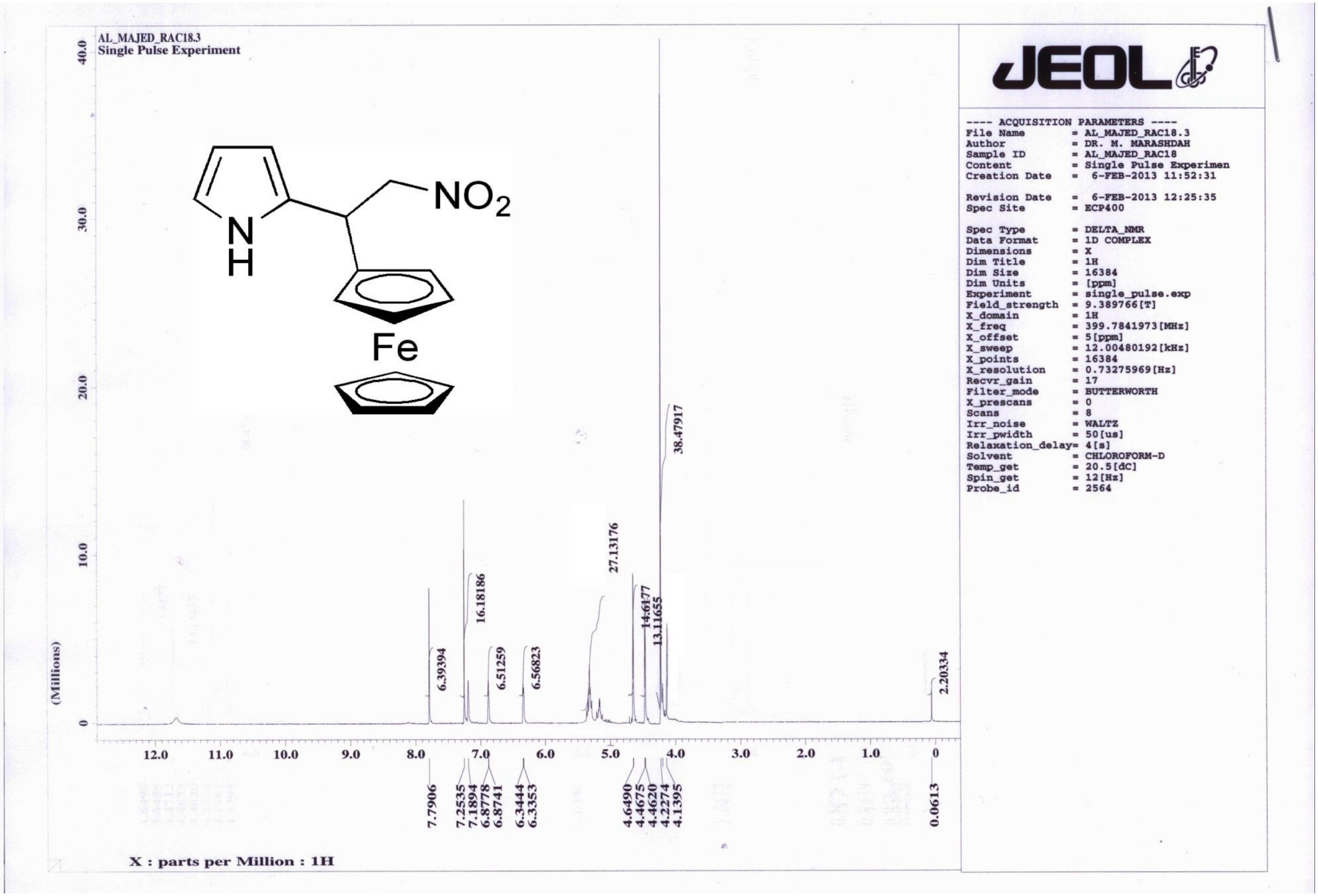

Compound 8i: ^{13}C-NMR

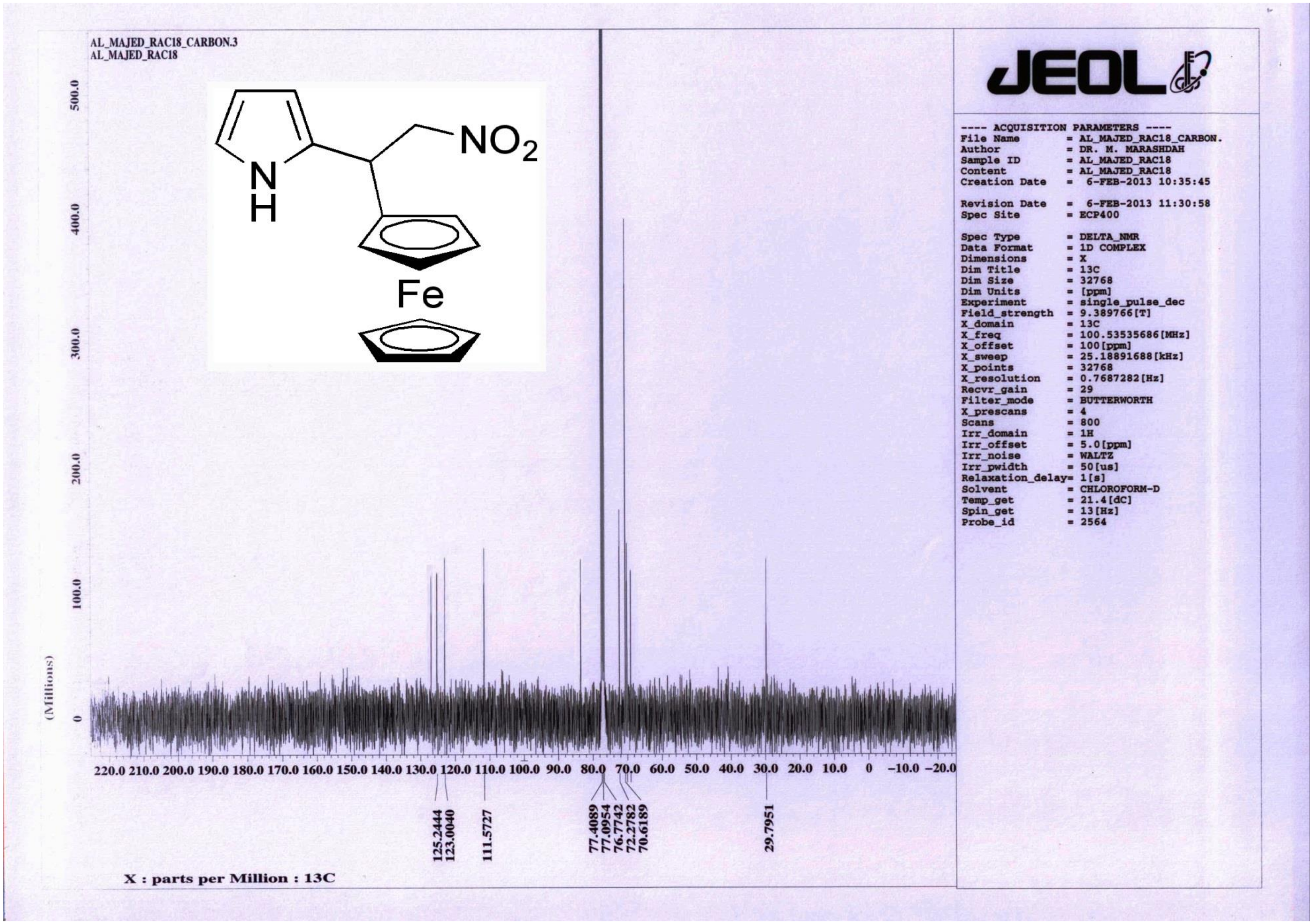

Supplement: Supplementary file 1 — Scan copies of 1H and 13C spectra of the Michael adducts 7a-i, 8a-I, 9a-d, & 9h [file 649197.f1.pdf]
